# Arsolyl-supported intermetallic dative bonding†

**DOI:** 10.1039/d2sc01200f

**Published:** 2022-05-04

**Authors:** Ryan M. Kirk, Anthony F. Hill

**Affiliations:** Research School of Chemistry, Australian National University Canberra A.C.T. Australia a,hill@anu.edu.au

## Abstract

The first examples of late transition metal η^5^-arsolyls 
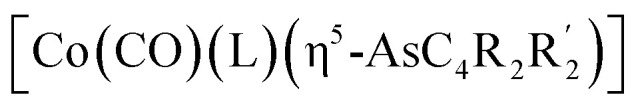
 (L = CO, P(OMe)_3_; R = Ph, Me, Et, SiMe_3_; R′ = Ph, H, Me, Et, Me) serve as ditopic donors to extraneous metal centres (M = Pt^II^, Au^I^, Hg^II^) through both conventional As → M and polar-covalent (dative) Co → M interactions.

## Abstract 2

Cobalt carbonyl reacts with arsoles to provide the first late transition metal η^5^-arsolyls. These serve as ditopic donors to extraneous metal centres (M = Pt^II^, Au^I^, Hg^II^) through both conventional AsM and polar-covalent (dative) CoM interactions.

## Graphical abstract

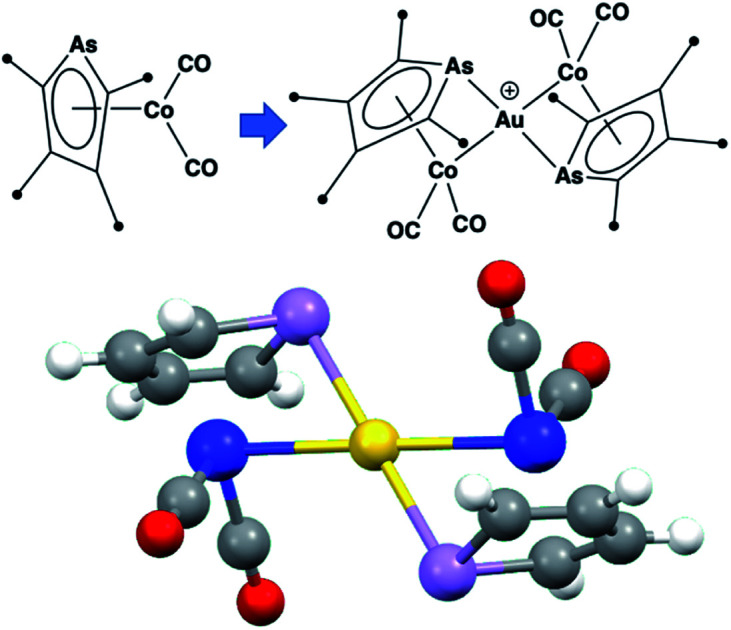

## Introduction

Transition metal complexes in which an arsolyl ligand ‘AsC_4_R_4_’ acts as a pentahapto cyclopentadienyl mimic are limited to [M(CO)_3_(η^5^-AsC_4_Ph_4_)] (M = Mn, Re) and a small number of structurally characterised ferrocene analogues [Fe(η^5^-AsC_4_Me_*n*_H_4−*n*_)_2_] (*n* = 0, 2, 4),^1^ all of which involve *d*^6^-pseudo-octahedral metal centres. This is in contrast to the chemistry of η^5^-phospholyls which is richly diverse and well-charted across the entire periodic system.^2^ Of late, the question of intermetallic polar-covalent (dative) bonding has received considerable attention,^3^ but finds its origin in the early observation that *d*^8^-[Co(CO)_2_(η-C_5_H_5_)] forms a Lewis base/acid adduct with HgCl_2_.^4^ While championing the concept of “Metal Bases *par excellence*”^5^ Werner placed particular emphasis on group 9 complexes of the form [ML_2_(η-C_5_H_5_)], recalling the archetypal Lewis basicity of [Co(CO)_2_(η-C_5_H_5_)]. Given that η^5^-phospholyls may on occasion display *P*-centred nucleophilicity,^6^ and that Mathey has established the viability of late transition metal η^5^-phospholyls, *e.g.*, [Co(CO)_2_(η^5^-PC_4_Ph_2_H_2_)],^7^ we have considered whether currently unknown arsolyl complexes of late transition metals with higher d-occupancies might also be viable. Specifically, we were intrigued to explore whether these might also serve as Lewis bases towards other metal centres and to what extent the arsenic donor, being typically less nucleophilic than in phosphorus congeners, might augment, support, or competitively compromise resulting metal–metal bonding. Accordingly, we report herein the isolation of the first late transition metal η^5^-arsolyl complexes and demonstrate their proclivity towards bridge-assisted metal–metal bond formation.

**Scheme 1:**
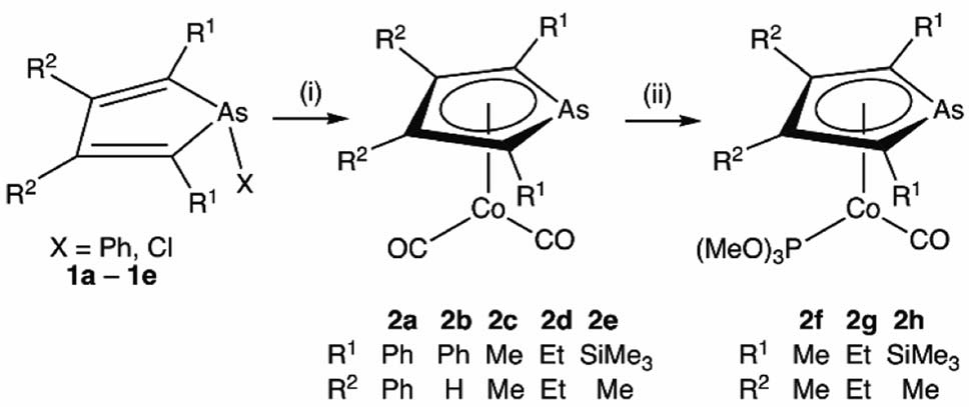
Synthesis of η^5^-arsolyl complexes of cobalt. (i) [Co_2_(CO)_8_], Δ, THF or *n*-hexane. (ii) P(OMe)_3_, 100 °C, toluene.

Heating [Co_2_(CO)_8_] and a selection of *As*-phenyl arsoles (1a–e, Scheme 1) in refluent THF or *n*-hexane provides the highly air-sensitive arsolyl complexes [Co(CO)_2_(η^5^-AsC_4_R_4_)] (2a–e) in modest yields following strictly anaerobic chromatography. Efforts to increase the isolated yields of 2a–e with extended reaction times or increased reaction temperatures were unsuccessful, however slightly increased yields were obtained by instead employing the more reactive As-chloro arsoles (see ESI†). The precise fate of the As-substituent during these reactions was not evident from the significant quantities of intractable materials also produced. The cleavage of an As–Ph bond from an arsole has on one previous occasion been observed in the reaction of [Mn_2_(CO)_10_] with PhAsC_4_Me_2_H_2_, albeit under rather more forcing conditions (150 °C, 4 hours).^1*c*^ Sub-optimal yields notwithstanding, the syntheses of 2a–e were highly reproducible, affording complexes 2a and 2b as orange solids or 2c–e as dark orange-red liquids at ambient temperature; the latter group underwent substitution with trimethylphosphite in toluene at 100 °C to provide the bright orange crystalline complexes [Co(CO){P(OMe)_3_}(η^5^-AsC_4_R_4_)] (2f–h) in high yield.

**Fig. 1:**
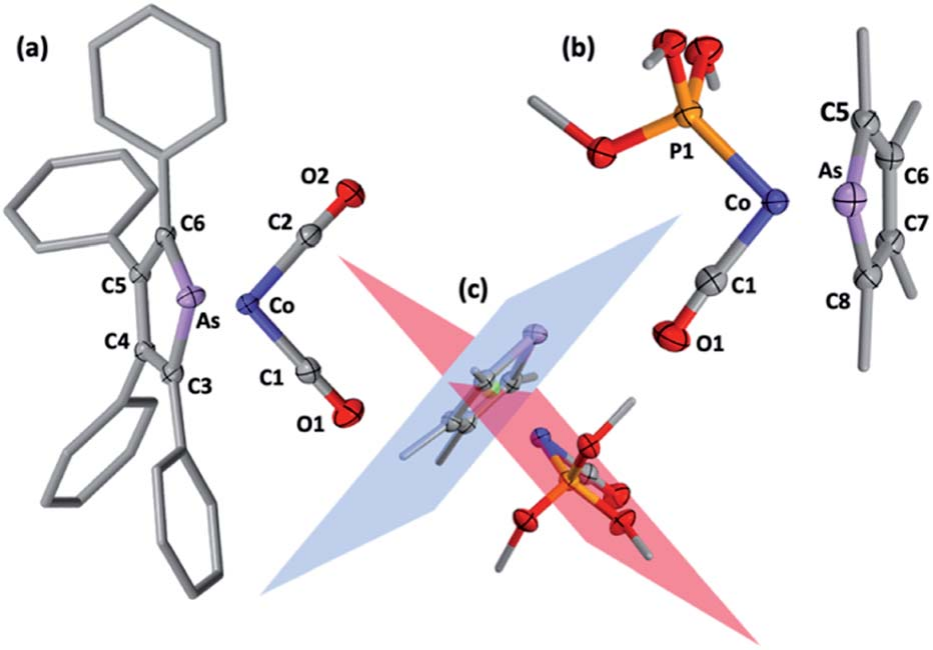
Molecular structures of (a) 2a and (b) 2f (50% displacement ellipsoids, arsenolyl ring and phosphite substituents simplified). Selected distances [Å] and angles [°]: 2a As1–Co1 2.427(4), As1–C3 1.900(2), C3–C4 1.443(3), C4–C5 1.427(3), C5–C6 1.439(3), C6–As1 1.915(2), Co1–C1 1.746(3), Co1–C2 1.747(3), C3–As1–C6 84.52(8), As1–C3–C4 114.32(1), C3–C4–C5 113.65(2), C4–C5–C6 112.84(2), C5–C6–As1 114.38(1), C1–Co1–C2 90.82(1); 2f As1–Co1 2.408(5), As1–C5 1.910(3), C5–C6 1.430(4), C6–C7 1.423(4), C7–C8 1.427(5), C8–As1 1.903(3), Co1–C1 1.719(3), Co1–P1 2.097(9), C5–As1–C8 84.12(1), As1–C5–C6 114.25(2), C5–C6–C7 113.30(3), C6–C7–C8 113.47(3), C7–C8–As1 114.56(2), C1–Co1–P1 93.49(1). (c) Intersection of C5–C6–C7–C8 (blue) and P1–Co–C1–C2 (red) planes for 2f at 89.7° with As, C5, C6, C7, C8 centroid in green.

Selected spectroscopic data for 2a–e and other germane cobalt(i) dicarbonyl complexes are presented in Table 1. We note that *ν*_CO_ frequencies for 2a–e fall between those for [Co(CO)_2_(η^5^-C_5_H_5_)] and [Co(CO)_2_(η^5^-C_5_Me_5_)], being comparable to those for η^5^-phospholyl cobalt dicarbonyl complexes reported by Mathey.^7^ The ^13^C{^1^H} NMR shifts for the CO ligands in 2a–e mirror the trends in *ν*_CO_ frequencies with a gradual shift to higher frequency and are not dissimilar to those for [Co(CO)_2_(η^5^-C_5_R_5_)] (R = H, 204.7; R = Me, 207.9 ppm) and [Co(CO)_2_(η^5^-PC_4_^*t*^Bu_2_H_2_)] (204.0 ppm).^7^ Comparison of ^13^C{^1^H} NMR data of the ring-carbon nuclei to those of the corresponding free arsoles 1a–e (ESI†) reveals a shift to low frequency of 20–30 ppm. The molecular structures of 2a, 2b, 2f, and 2h were crystallographically determined, with two representative examples, 2a and 2f, depicted in Fig. 1 (for 2b and 2h see ESI†).

**Table 1:** Selected spectroscopic data for complexes prepared in this work, and some previously reported complexes for comparison

Entry	Complex	^13^C *δ*_C-α_^a^	^13^C *δ*_C-β_^a^	^13^C *δ*_CO_^a^	*ν*_CO_ b (*sym*)	*ν*_CO_ b (*asym*)	*k*_CO_ c
2a	Co(CO)_2_(η^5^-AsC_4_Ph_4_)	137.2	135.1	203.8	2026	1978, 1970^d^	16.13
2b	Co(CO)_2_(η^5^-AsC_4_Ph_2_H_2_)	138.1	94.9	203.0	2032	1985, 1976^d^	16.23
2c	Co(CO)_2_(η^5^-AsC_4_Me_4_)	118.9^e^	113.1^e^	205.1^e^	2019	1967	16.02
2d	Co(CO)_2_(η^5^-AsC_4_Et_4_)	128.4^e^	118.8^e^	205.3^e^	2017	1965	15.99
2e	Co(CO)_2_{η^5^-AsC_4_(SiMe_3_)_2_Me_2_}	122.2^e^	119.8^e^	204.5^e^	2015	1962	15.95
2f	Co(CO){P(OMe)_3_}(η^5^-AsC_4_Me_4_)	114.3	109.8	206.8	1947		15.39
2g	Co(CO){P(OMe)_3_}(η^5^-AsC_4_Et_4_)	124.4	115.3	207.3	1945		15.36
2h	Co(CO){P(OMe)_3_}{η^5^-AsC_4_(SiMe_3_)_2_Me_2_}	118.7	116.1	205.5	1941		15.30
	Co(CO)_2_(η^5^-C_5_H_5_)^11^	84.5^e^		205.6^e^	2033^f^	1972^f^	16.17
	Co(CO)_2_(η^5^-C_5_Me_5_)^11^	96.7^e^		207.9^e^	2011	1949	15.81
	Co(CO)_2_(η^5^-C_5_Ph_5_)^8^	No data reported			2000	1945	15.69
	Co(CO)_2_(η^5^-C_5_{CH_2_Ph}_5_)^8^	No data reported			2020	1960	15.97
	Co(CO)_2_(η^5^-PC_4_Ph_2_H_2_)^9^	No data reported			2030	1980	16.21
	Co(CO)_2_(η^5^-PC_4_^*t*^Bu_2_H_2_)^10^	136.2	91.9	204.0	2023	1968	16.06
	Co(CO){P(OMe)_3_}(η^5^-PC_4_^*t*^Bu_2_H_2_)^10^	134.7	90.5	208.0	No data reported		—

a C_6_D_6_ solution unless otherwise stated, ppm downfield from SiMe_4_, 25 °C; the labels α and β refer to ring-carbon positions with respect to the heteroatom (where applicable).

b *n*-Hexane solution unless otherwise stated, cm^−1^, 25 °C.

c Cotton–Kraihanzel force constant in N cm^−1^.

d Resolution of the doubly degenerate E vibrational mode is observed in *n*-hexane for these complexes *cf*., *ν*_CO_ (CH_2_Cl_2_): 2a 2022, 1963 cm^−1^; 2b 2027, 1972 cm^−1^.

e CDCl_3_ solution, 25 °C.

f Cyclohexane solution.

The structural models for 2a, 2b, 2f and 2h all confirm the targeted η^5^-arsolyl coordination. In 2a and 2f the ligands are almost symmetrically disposed with respect to the vertical plane which bisects the η^5^-arsolyl ring, whereas for 2b and 2h these are rotated to a position slightly offset from the arsenic-ring centroid vector (ESI†). Consistent with the difference in the covalent radii of carbon (0.76 Å) and arsenic (1.19 Å), the latter is in each case very slightly displaced (3–5°) from the mean plane defined by the heterocycle carbon atoms, though less than found in the free arsoles (1a: 10.2°; 1b: 7.10°).^10^ The geometry of the metal and η^5^-arsolyl rings in 2a, 2b, 2f and 2h are of a distorted pentagonal pyramid. The C_α_–As–C_α_ angles at the arsenic vertices are significantly contracted (84–87°) from that of an idealised pentagon (108°) while the remaining C–C–C angles are in the range 111–114°, and comparable to those found in [Co(CO)_2_(η^5^-C_5_R_5_)] (R = Me, Ph, CH_2_Ph).^8^

**Fig. 2:**
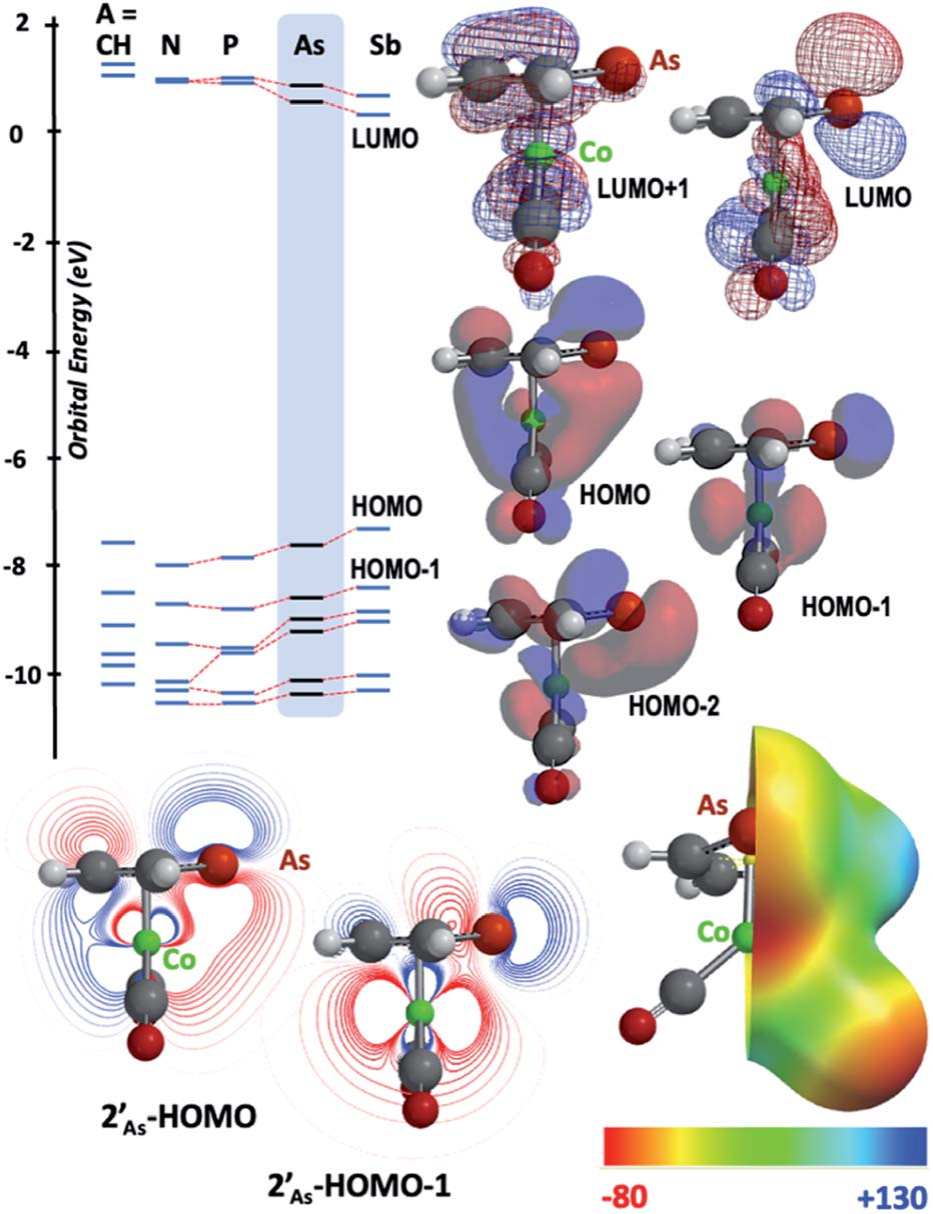
Frontier orbitals of interest for [Co(CO)_2_(η^5^-AC_4_H_4_)] (
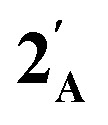
: A = CH, N, P, As, Sb; DFT:ωB97X-D/6-31G*/LANL2Dζ). Inset = electrostatic potential map for [Co(CO)_2_(η^5^-AsC_4_H_4_)] 
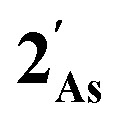
 indicating site for charge-controlled electrophilic attack. Condensed Fukui functions (natural populations) for electrophilic attack: *f*^−^(Co) = 0.113, *f*^−^(As) = 0.251.

The results of computational interrogation of the model ‘parent’ compound [Co(CO)_2_(η^5^-AsC_4_H_4_)] 
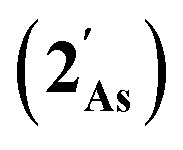
 (DFT:ωB97X-D/6-31G*/LANL2Dζ; ESI†) are summarised in Fig. 2 alongside those for [Co(CO)_2_(η^5^-C_5_H_5_)] 
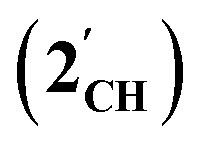
, and the hypothetical pnictogenyl analogues [Co(CO)_2_(η^5^-AC_4_H_4_)] 
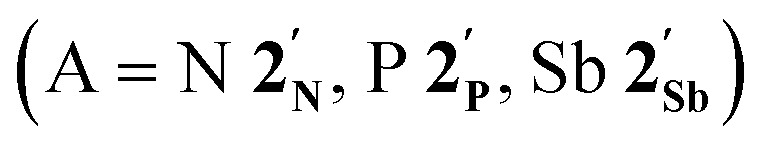
. The HOMO−1 is in all cases substantially derived from the 
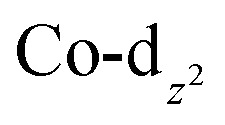
 orbital and readily corroborates the known nucleophilic behaviour of 
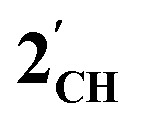
. For 
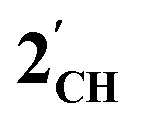
 this is, however, effectively the only orbital that is geometrically disposed to allow the complex to function as a Lewis base since the HOMO is involved with cyclopentadienyl binding. This is also the case with the HOMO of the pnictogenolyl examples however the orbital substantially protrudes radially from the ring. The HOMO−1 involves substantial contribution from the pnictogen orbital such that both the HOMO and HOMO−1 (and also HOMO−2) contribute to a prominent region of electron density localised over these atoms which is reflected in the electrostatic potential map for 
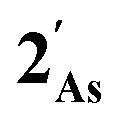
 and the condensed Fukui functions for both arsenic and cobalt (Fig. 2 inset). Furthermore, on descending group 15, the pnictogen A-p_*z*_ orbital increasingly contributes and this is accompanied by a monotonic increase in the energy the HOMO, HOMO−1 and HOMO−2 orbitals which should manifest as an increase in the basicity of not only the metal but also the pnictogen. This is intriguingly counterintuitive in that the basicity, nucleophilicity and strength of pnictogen coordination generally decreases for simple pnictanes AR_3_ traversing from P to Sb.^12^ Compared to phospholyl and arsolyl complexes, η^5^-stibolyl complexes are rarer still, being limited to three ferrocene analogues, but clearly worthy of further study, not least because of the onset of secondary bonding for the heavier pnictogens.^13^

**Scheme 2:**
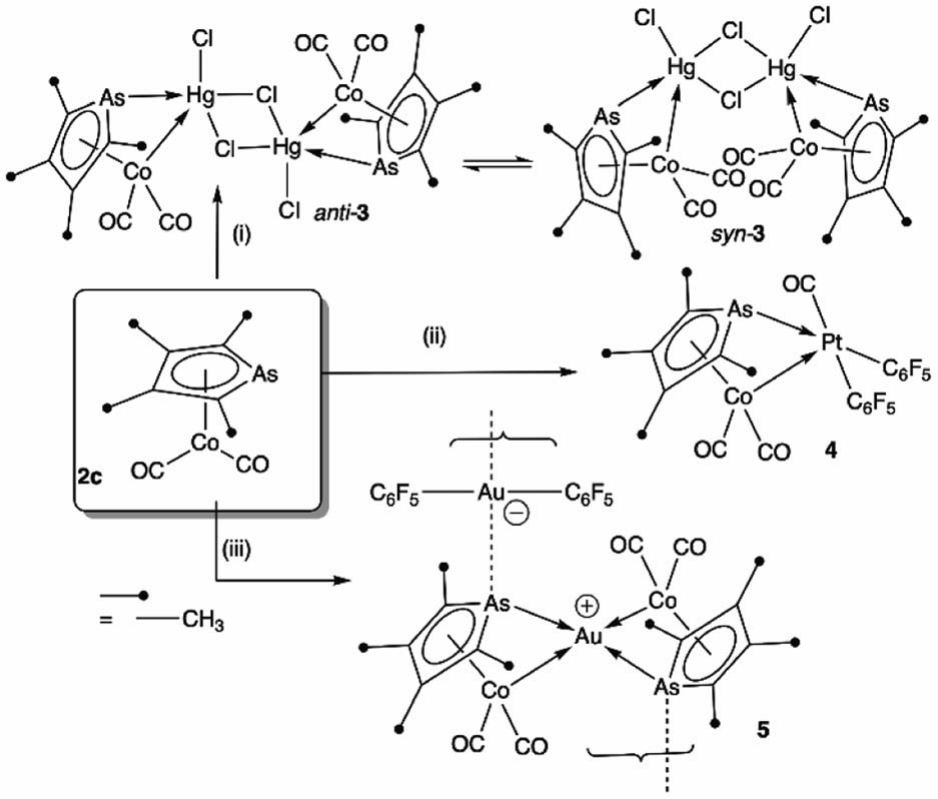
Bridge-assisted formation of dative bonds from cobalt to gold(i), mercury(ii) and platinum(ii). (i) HgCl_2_. (ii) **c*is*-[Pt(C_6_F_5_)_2_(hex)] (hex = 1,5-hexadiene). (iii) [Au(C_6_F_5_)(THT)] (THT = tetrahydrothiophene).

**Fig. 3:**
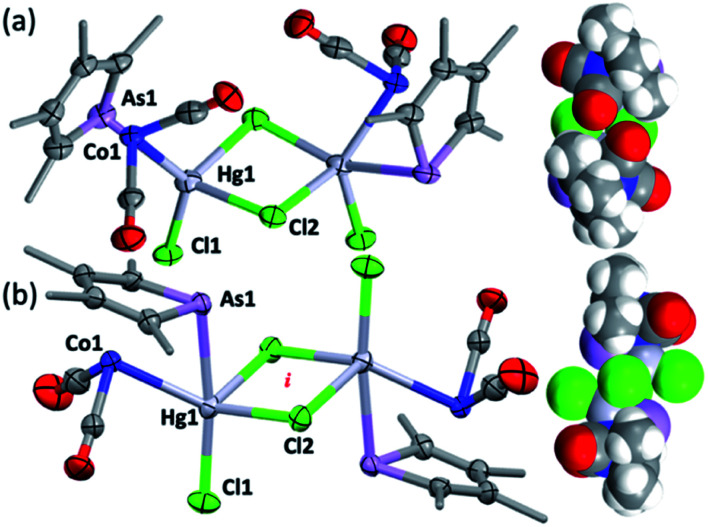
The molecular structures of (a) *syn*-3 and (b) *anti*-3 (methyl groups simplified, 50% displacement ellipsoids). Selected distances [Å] and angles [°]: (a) *syn*-3 As1–Hg1 2.7268(9), Co1–Hg1 2.620(1), Hg1–Cl1 2.491(2), Hg1–Cl2 2.598(2), Hg1–Cl2′ 2.708(2), As1–Hg1–Co1 55.86(3), Hg1–Cl2–Hg1′ 91.87(6), Cl2–Hg1–Cl2′ 87.62(6), Cl1–Hg1–Cl2 100.71(6). (b) *Anti*-3 As1–Hg1 2.6334(6), Co1–Hg1 2.6702(9), Hg1–Cl1 2.390(1), Hg1–Cl2 2.778(1), Hg1–Cl2′ 2.777(1) As1–Hg1–Co1 56.27(2), Hg1–Cl2–Hg1′ 84.37(4), Cl2–Hg1–Cl2′ 95.63(4), Cl1–Hg1–Cl2, 100.59(5). *i* = crystallographic inversion centre.

To explore the possibility of metal–metal bond-formation, the representative 2c was chosen, commencing with mercuric chloride by analogy with the prototypical and monomeric adduct [Co(HgCl_2_)(CO)_2_(η-C_5_H_5_)].^4^ The reaction of 2c with HgCl_2_ in acetone rapidly results in precipitation of the poorly soluble yellow dimer [2c·HgCl(μ-Cl)]_2_ (3) in high yield (Scheme 2 and Fig. 3).

The dimeric formulation follows from HR-ESI-MS data, which are devoid of ions due to dissociated 2c, in addition to crystallographic analyses of two isomers that differ in the μ:σ-η^5^-arsolyl rings adopting mutually *syn* or *anti* positions with respect to the rhomboidal Hg_2_(μ-Cl)_2_ core. Thus, yellow needles of *anti*-3 (major) and orange prisms of *syn*-3 (minor) slowly crystallise together from solutions of 3 in acetone stored at −30 °C.

One half of each of the dimeric structures of both *anti*-3 and *syn*-3 in the solid state is crystallographically unique due to the centre of the Hg_2_(μ-Cl)_2_ unit coinciding with either an inversion centre (*anti*-3 in *P*2_1_/*n*) or twofold rotation axis (*syn-*3 in *C*2/*c*). The coordination polyhedra of the Hg^II^ atoms are strikingly different in each isomer: *anti*-3 features severely distorted trigonal bipyramidal mercury with the arsenic and cobalt atoms assuming pseudo-axial and -equatorial positions, respectively (*τ*_5_ = 0.89), whereas for *syn*-3 the more sterically congested mercury geometry more closely approaches a square-based pyramid (*τ*_5_ = 0.62) with the non-bridging chlorides occupying the eclipsing apices. The As–Hg bond distances are somewhat shorter by *ca.* 0.1 Å in *anti*-3 (2.633(6) Å) than *syn*-3 (2.727(9) Å), while the Co–Hg bond distances of 2.670(9) Å (*anti*) and 2.620(1) Å (*syn*) are essentially equivalent within crystallographic precision limits. The latter pair are somewhat longer than the sum of covalent radii for the individual elements (2.58 Å) and the separation (2.578(4) Å) observed for [Co(HgCl_2_)(CO)_2_(η^5^-C_5_H_5_)],^4^ by virtue of the increased coordination number at mercury. The slight elongation of the Co → Hg interaction here is almost certainly a geometric compromise to accommodate the Hg^II^ centre within the disparate coordination spheres of the As^III^ and Co^I^ donors, rather than indicating any noteworthy electronic phenomena beyond non-directional spodium bonding.^14^ From a valence-bond perspective, 2c may be considered to serve as a neutral, 4-electron bidentate ligand with a somewhat narrow bite-angle (*ca.* 55–56°). Solution infrared data for each isomer (after manual separation of crystals) resulted in spectra identical to that of the bulk sample of 3 obtained above, *i.e.*, it appears that upon dissolution in acetonitrile, *syn*-3 isomerises to *anti*-3 and the latter conformation is the natural condition of the adduct. Given both isomers have identical QSAR volumes (536 Å^3^), the polarity of *syn-*3 (dipole = 23.5 D *cf.* 0 for *anti-*3) possibly plays a role in its crystal formation.

**Fig. 4:**
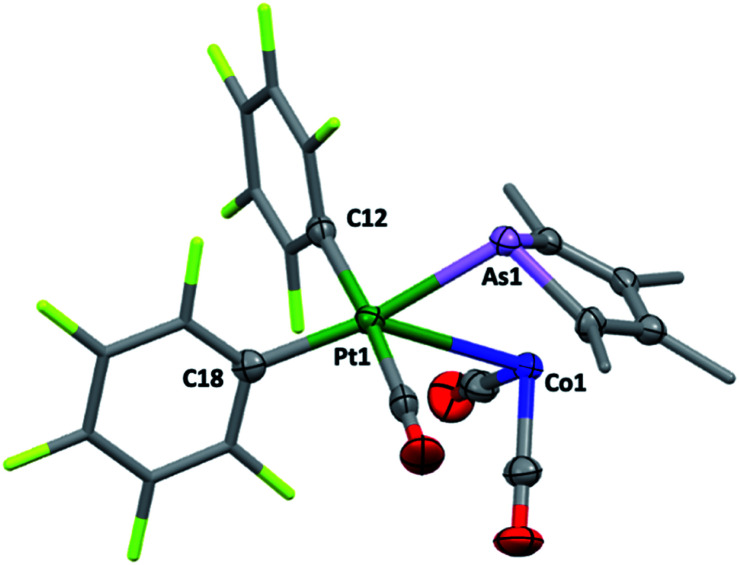
The molecular structure of 4 (methyl and pentafluorophenyl groups simplified, 50% displacement ellipsoids, Pt *TBPY*-5-12-*C* enantiomer in non-centrosymmetric *P*2_1_2_1_2_1_ space group). Selected distances [Å] and angles [°]: As1–Pt1 2.5185(8), Co1–Pt1 2.996(1), Pt1–C12 2.053(8), Pt–C18 2.077(7), As1–Pt1–Co1 53.04(3), As1–Pt1–C18 169.58(2), Co1–Pt1–C18 137.24(2).

Though their Lewis acidity is well-documented, neither [Hg(CF_3_)_2_] nor [Hg(C_6_F_5_)_2_] provided any evidence of detectable adduct formation with 2c. Coordination of 2c to divalent platinum(ii) could however be demonstrated in its reaction with *cis*-[Pt(C_6_F_5_)_2_(hex)] (hex = η^2^:η^2^-1,5-hexadiene) in CH_2_Cl_2_ to provide after anaerobic chromatography a single isolable orange compound in modest yield. The complex with three *ν*_CO_ absorptions at 2084, 2053 and 2014 cm^−1^ and the observation of six resonances in the ^19^F NMR (ESI†) confirmed the presence of two chemically inequivalent C_6_F_5_ groups inconsistent with a simple ‘2c·Pt(C_6_F_5_)_2_’ adduct. The X-ray diffraction analysis (Fig. 4) confirmed it to be [2c·*cis*-Pt(CO)(C_6_F_5_)_2_] (4) arising from CO sequestration.

The structure of 4, like *anti*-3 and *syn*-3, comprises 2c acting as a bidentate ligand (chelate bite = *ca.* 53°) to a five-coordinate ‘Pt(CO)(C_6_F_5_)_2_’ centre. Putting aside the Co → Pt interaction, the platinum adopts a square-planar geometry (inset, Fig. 4). The Pt(ii) centre is slightly displaced from the ligand donor mean plane (*trans* angles 169.58(2)°, 174.58(3)°) towards the dative Co → Pt interaction which is remarkably long (Co1–Pt1: 2.966(1) Å), falling between the sums of covalent (2.62 Å) and van der Waals (*ca.* 4.01 Å) radii for these elements. The As1–Pt1 bond length of 2.519(8) Å is also significantly elongated relative to more conventional platinum-bound arsines *e.g.*, [PtCl_*x*_(C_6_F_5_)_2−*x*_(L)_*n*_] (*n* = 1, 2; *x* = 0,1; L = CH_2_(AsPh_2_)_2_, C_2_H_4_(AsPh_2_)_2_: 2.34–2.43 Å).^15^ Despite being apparently rather weak, it is this interaction which completes the square coordination plane.

**Fig. 5:**
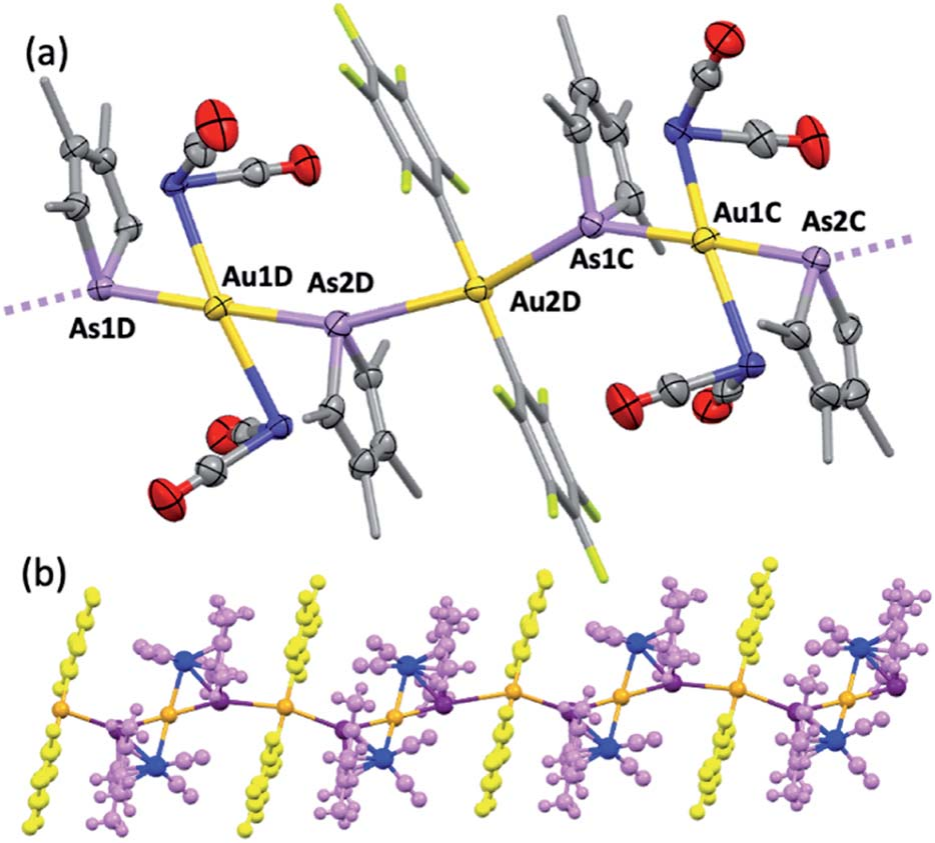
The molecular structure of 5 (methyl and pentafluorophenyl groups simplified and hydrogen atoms omitted for clarity, four crystallographically independent molecules in the unit cell, 50% displacement ellipsoids). Selected distances [Å]: As1–Au1 2.5215(6), As2–Au1 2.5205(6), As1–Au2 3.1660(6), Co1–Au1 2.6793(9), Co2–Au1 2.6731(8). Selected angles [deg]: As1–Au1–Co1 57.89(2), As2–Au1–Co2 57.67(2), As1–Au1–As2 178.99(2), Co1–Au1–Co2 167.70(3). (b) View orthogonal to the *b*-axis showing extended chain of (⋯Au⋯As)_∞_ interactions (3.0746(6)–3.2122(6) Å).

Further exemplifying the bidentate nature of 2c, combination with one equivalent of [Au(C_6_F_5_)(THT)] (THT = tetrahydrothiophene) leads to the isolation of a yellow solid of deceptively simple composition “2c·Au(C_6_F_5_)” that is actually the salt [(2c)_2_Au][Au(C_6_F_5_)_2_] (5, Fig. 5). The crystal structure of 5 reveals bidentate coordination of two 2c units to a Au^+^ cation with near to coplanar coordination of the Co and As donors (Au sits 0.22 Å above the Co_2_As_2_ mean plane). The 2c ‘ligands’ are asymmetrically disposed about the Au^+^ cation, being transposed and offset by *ca.* 22° when viewed along the As1–Au1–As2 vector. The As–Au distances are equivalent (mean 2.525 Å), with slightly longer Co–Au distances (mean 2.650 Å). The unit cell of 5 contains four crystallographically independent [(2c)_2_·Au][Au(C_6_F_5_)_2_] pairs (ESI†) with extended packing of loosely parallel columns of alternating [(2c)_2_·Au]^+^ cations and [Au(C_6_F_5_)_2_]^−^ anions. The nature of their arrangement differs slightly between pairs, with notably short As⋯[Au(C_6_F_5_)_2_]^−^ interactions (between the arsolyl arsenic and [Au(C_6_F_5_)_2_]^−^ gold atoms). These distances are, however, not equal, and capriciously vary between 3.0746(6) Å (shortest) and 3.2122(6) Å (longest).

Computational interrogation (ωB97X-X/6-31G*/LANL2Dζ) of the model complexes [CoPt(μ-AsC_4_H_4_)(CO)_3_(CF_3_)_2_] (4′) and [Co_2_Au(μ-AsC_4_H_4_)_2_(CO)_4_]^+^ (5′) (detailed in the ESI†) returns core geometries close to those of 4 and the cation of 5. This analysis reveals molecular orbitals of interest (see ESI Fig. S12–S14†) that account for the geometrical features of note. For 4′, the HOMO and HOMO−7 comprise significant overlap of the 
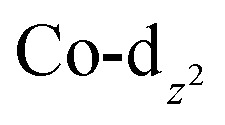
 orbital with platinum, supporting a Co → Pt description (Löwdin bond orders As–Pt/Co–Pt = 0.75/0.41). For 5′ not only does the HOMO−16 adhere to the view of dative Co → Au^+^ bonding (Löwdin bond orders As–Au/Co–Au = 0.78/0.56), but an orbital (HOMO−20) has a topology suggestive of the arsenolyl serving as a π-acceptor from gold, a feature also present in the MO scheme for 4′. Furthermore, the LUMO which has substantial arsenic character would appear to account for the association of the [Au(C_6_F_5_)_2_]^−^ anion at this point which underpins the extended polymeric assembly, though it is unlikely that this persists in solution.

## Conclusions

The first (eight) examples of late transition metal η^5^-arsolyl complexes have been obtained with one example being then employed to explore the possibility of both the metal and arsenic serving as donors to Pt^II^, Au^I^ and Hg^II^ centres. Although the individual interactions might appear weak, when both act in concert novel bridge-assisted heterometallic assemblies arise with intriguing features.

## Data availability

Crystallographic data for structurally characterised compounds have been deposited at the Cambridge Crystallographic Data Centre under CCDC 2130783–2130790 and can be obtained from https://www.ccdc.cam.ac.uk. Spectroscopic data for all new compounds are provided in the ESI accompanying this paper (https://doi.org/10.1039/d2sc01200f).

## Author contributions

RMK was responsible for the conceptualisation and execution of the experimental research, the acquisition and critical analysis of the characterisational data and compilation of the original draft. AFH was responsible for funding acquisition, project administration, validation and refinements to the manuscript.

## Conflicts of interest

There are no conflicts to declare.

## Supplementary Material

SC-013-D2SC01200F-s001

SC-013-D2SC01200F-s002
