# Supplementary material for: Arsolyl-supported intermetallic dative bonding
Source: Chem Sci. 2022 May 4;13(23):6830–5. doi: 10.1039/d2sc01200f (PMC9200119; doi:10.1039/d2sc01200f)
Supplement: SC-013-D2SC01200F-s001 [file SC-013-D2SC01200F-s001.pdf]

Electronic Supplementary Material (ESI) for Chemical Science.
This journal is © The Royal Society of Chemistry 2022

Arsolyl-Supported Intermetallic Dative Bonding

Ryan M. Kirk and Anthony F. Hill*

Abstract: The first examples of late transition metal η[5]-arsolyls serve as ditopic donors to extraneous metal centres (M = Pt^{II}, Au^{I}, Hg^{II}) through both conventional As→M and polar-covalent (dative) Co→M interactions.

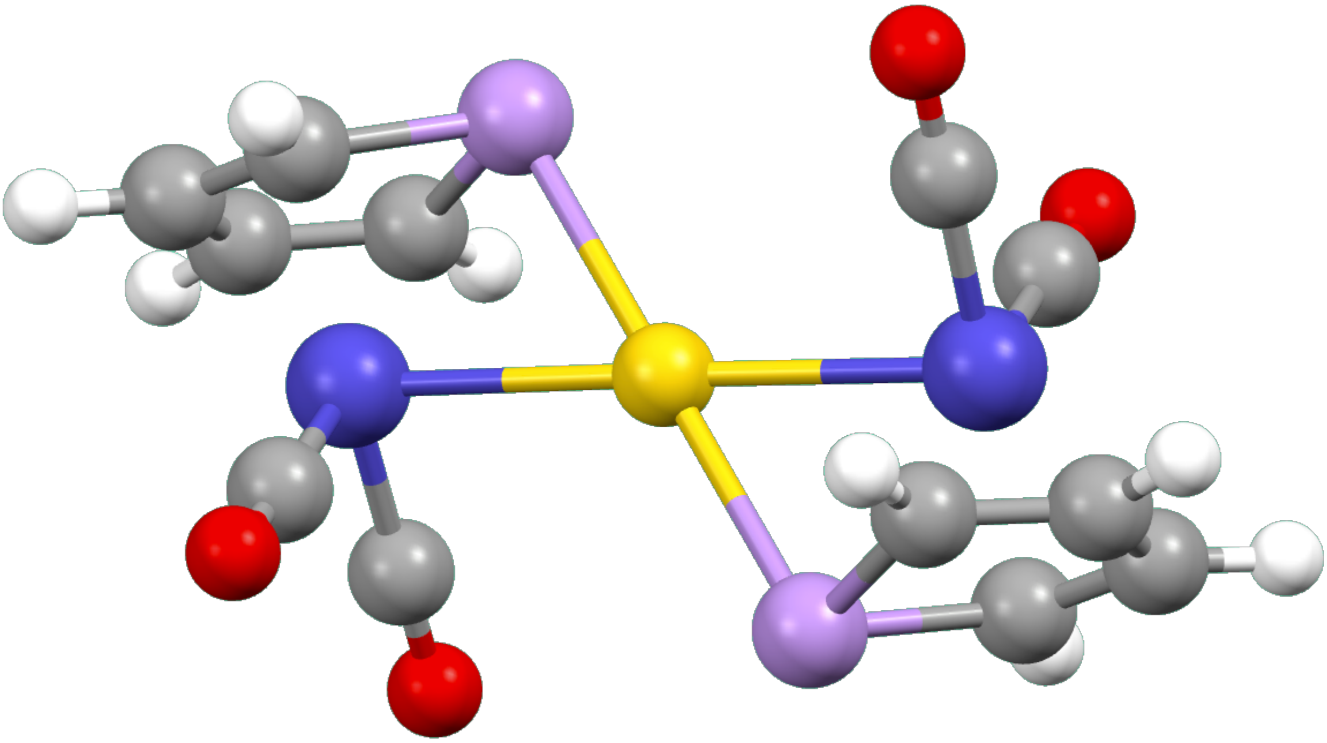

Table of Contents

1. General Considerations and Instrumentation
2. Computational Details
3. Additional Tables and Figures
4. Synthetic Procedures and Spectroscopic and Crystallographic Data
5. Selected Spectra
6. Author Contributions
7. References

Experimental Procedures

1. General Considerations

NMR data were collected on a Bruker Avance 400 (^{1}H at 400.1 MHz, ^{19}F at 376.5 MHz, ^{31}P at 161.9 MHz) or Bruker Avance 700 (^{13}C at 176.1 MHz, ^{195}Pt at 149.8 MHz) spectrometers at the temperature indicated; chemical shifts (δ) are given in ppm with coupling constants in Hz and are referenced to the residual protio-solvent impurity signal (for ^{1}H), the solvent signal itself (for ^{13}C) or an external reference ($CFCl_3$ for ^{19}F, 85% H_3PO_4 for ^{31}P, 1.2 M Na_2PtCl_6 (δ_{Pt} = 0) for ^{195}Pt). Spectra provided generally correspond to samples obtained directly from chromatography and occasionally contain residual solvent signals. Phosphorus-31 resonances were somewhat broadened due to coupling to quadrupolar (I = $^7/_2$) ^{59}Co nuclei. The multiplicities of NMR resonances are denoted by the abbreviations s (singlet), d (doublet), t (triplet), m (multiplet), br (broad) and combinations thereof for more highly coupled systems; we thank Dr Doug Lawes for assistance and helpful discussion. Solution infrared spectra were collected using a PerkinElmer Spectrum One FT-IR spectrometer with 1 mm cell pathlength and KBr windows. Elemental microanalytical data were provided by Elemental Microanalysis Service at Macquarie University. High-resolution electron ionisation (EI) and electrospray ionisation (ESI, +ve ion) mass spectrometry were performed by Mrs Anitha Jeyasingham at the ANU Research School of Chemistry mass spectrometry service with acetonitrile matrices. X-ray crystallography was performed on an Agilent Technologies Supernova EosS2-CCD diffractometer and specific details for each crystalline specimen are given below; we thank Dr Michael Gardiner for assistance and helpful discussion.

Unless otherwise stated, all operations were performed under an atmosphere of commercially purified nitrogen or argon using standard Schlenk techniques, or inside an argon-filled glovebox ([O_2] < 5 ppm, [H_2O] < 1 ppm); all compounds described herein are air-sensitive though some (unless otherwise stated) appear immune to adventitious moisture. Caution should be exercised when handling the organoarsenic compounds described herein – their toxicological properties are presently unknown, and they should be regarded as extremely toxic materials. The hazardous toxicological properties of $PhAsCl_2$, $AsCl_3$, Bu_2SnCl_2 and $HgCl_2$ are well known, and they too should be handled with respect by trained persons equipped with appropriate safety equipment. Benzene (including C_6D_6) is a potent carcinogen and should only be used in a well-ventilated fumehood in quantities as small as is reasonably possible. Residues containing heavy metals or metalloids should be disposed of appropriately with consideration given to the ecological impact of these elements. Care should also be exercised when storing solvents over alkali metal surfaces for their extreme flammability. HPLC-grade solvents were obtained from Merck; THF, benzene, toluene and Et_2O were distilled from sodium-benzophenone ketyl under nitrogen and stored over sodium (THF, Et_2O) or potassium (benzene, toluene) mirrors. C_6D_6 (Cambridge Isotope Laboratories) was degassed with argon and stored over potassium mirror. $CDCl_3$ and CD_2Cl_2 (Cambridge Isotope Laboratories) were distilled from anhydrous $CaSO_4$ under argon and stored over 4 Å molecular sieves. *n*-hexane and *n*-pentane (except for air-free chromatography, *vide infra*) were distilled from CaH_2 under nitrogen and stored over potassium mirrors. Acetonitrile, CH_2Cl_2 and $SiMe_4$ were distilled from CaH_2 under nitrogen and stored over 4 Å molecular sieves. Acetone was distilled from anhydrous $CaSO_4$ under nitrogen and stored over 4 Å molecular sieves.

The arsoles **1a** ($PhAsC_4Ph_4$), **1b** ($PhAsC_4Ph_2H_2$), **1c** ($PhAsC_4Me_4$), 1d ($PhAsC_4Et_4$) and 1e ($ClAsC_4(SiMe_3)_2Me_2$) were synthesised from the corresponding zirconacyclopentadiene in THF (**1a**, **1b**, with catalytic CuCl) or hydrocarbon (**2c**, **2d**, **2e**) solution; spectra were in agreement with those reported.[1] Arsoles **1a** and **1b** are air-stable crystalline yellow solids whilst **1c**, **1d** and **1e** are air-sensitive pale-yellow liquids. The air- and moisture-sensitive *As*-chloro analogues of **1c** and **1d** were obtained by use of $AsCl_3$ instead of $PhAsCl_2$. *Note*: $ClAsC_4Me_4$ gradually decomposes at ambient temperature and should ideally be used the same day as it is prepared. It is thus advisable to carry out addition of $AsCl_3$ to $Cp_2ZrC_4Me_4$ in hydrocarbon solution at -40 °C and perform workup in a timely manner. This decomposition was not observed for $ClAsC_4Et_4$ and $ClAsC_4(SiMe_3)_2Me_2$ and these could be stored at -30 °C for several weeks without noticeable diminution in quality. The known *As*-chloro analogue of **1a**[2] was prepared by a new method from $Bu_2SnC_4Ph_4$ (itself prepared from the zirconacyclopentadiene and Bu_2SnCl_2 with catalytic CuCl in THF, isolated as a reasonably air-, moisture-, and alumina-stable viscous yellow liquid) and stoichiometric $AsCl_3$ in *n*-hexane solution over 48 h from which the product precipitates in good yield as a yellow solid – is it indefinitely stable under inert atmosphere at ambient temperature though it is rather air- and moisture-sensitive. Spectroscopic data for $ClAsC_4Ph_4$: NMR ($CDCl_3$, 25 °C), ^{1}H (400 MHz) δ 7.19—7.15 (m, 10H, C_6H_5), 7.10—7.04 (m, 6H, C_6H_5), 6.88—6.85 (m, 4H, C_6H_5); ^{13}C{^{1}H} (176 MHz) δ 152.0 (C_α), 149.5 (C_β), 139.2 (C_6H_5), 137.1 (C_6H_5), 129.9 (C_6H_5), 129.4 (C_6H_5), 128.4 (C_6H_5), 128.0 (C_6H_5), 127.5 (C_6H_5), 127.4 (C_6H_5) – some aromatic ^{13}C resonances appear to be isochronous hence fewer signals are observed than expected; HR-MS (EI, MeCN) found *m*/*z* 466.0462 (calc. for $C_{28}H_{20}{}^{75}As_1{}^{35}Cl_1$ $[M]^+$: 466.0469). The crystal structure of $ClAsC_4Ph_4$ has been determined and will be reported separately in a forthcoming article. $Au(C_6F_5)$(THT) and $Pt(C_6F_5)_2(\eta^4\text{-}C_6H_{10})$ were prepared from AuCl(THT) and $PtCl_2(\eta^4\text{-}C_6H_{10})$ by addition of Li[C_6F_5] in Et_2O (slight excess, prepared *in-situ* from C_6F_5Br and *n*-BuLi) at -78 °C and purified by flash chromatography (neutral alumina, Et_2O) and re-crystallisation from Et_2O/*n*-hexane as white (Au) or cream (Pt) solids; both are air- and moisture-stable (^{19}F{^{1}H} (376 MHz),

$CDCl_3$, 25 °C: Au(C_6F_5)(THT) δ -116.1 (m, 2F), -159.6 (m, 1F), -162.4 (m, 2F); Pt(C_6F_5)$_2$(η^4-C_6H_{10}) δ -120.4 (m, 2F), -160.2 (m, 1F), -165.3 (m, 2F)). $PhAsCl_2$ was prepared by addition of PhMgCl (2 M in Et_2O) to $AsCl_3$ (caution – vesicant and poison) in Et_2O at 0 °C and purified by short-path vacuum distillation, isolated as an air-sensitive colourless liquid (caution – vesicant and poison). Other reagents were obtained from commercial suppliers and used without further purification: Cp_2ZrCl_2 and $Co_2(CO)_8$ (Strem); phenylacetylene, 2-butyne and 3-hexyne (Alfa Aesar); diphenylacetylene, 1-(trimethylsilyl)prop-1-yne, anhydrous $HgCl_2$, $AsCl_3$, Bu_2SnCl_2 and $P(OMe)_3$ (Sigma Aldrich).

General procedure for anaerobic chromatography: Silica gel (230—400 mesh; Merck), neutral alumina (activity I, 70—230 mesh; Merck) and Florisil® (magnesium silicate, 60-100 mesh; Merck) were permanently stored in a 100 °C oven; *n*-pentane for chromatography need not be stringently anhydrous for the work below, and permanent storage over 4 Å molecular sieves or sodium wire was sufficient – it should, however, be thoroughly degassed with nitrogen prior to use. Anaerobic chromatography was carried out in either 40 x 2.5 cm or 30 x 1 cm water-jacketed (water temp. typically *ca.* 8—10 °C) Schlenk columns with 250 mL or 100 mL reservoir bulbs, respectively. Columns were charged with the desired quantity of oven-dried chromatography material and the entire apparatus held under vacuum for 30 minutes (twice), before being flushed with a gentle flow of nitrogen for 15—30 minutes. Degassed *n*-pentane is then admitted *via* cannula and allowed to run down the column under a positive pressure of nitrogen until the solid phase is sufficiently packed (gentle and careful external agitation of the apparatus may be required to help material settle). Following this, the mixture to be chromatographed is added *via* syringe to the column. Alternatively, the mixture to be chromatographed may be adsorbed onto a small amount of oven-dried silica gel/alumina/Florisil® by evaporation of solvent, and this material can be transferred to the top of the column under gentle counter-flow of nitrogen. The procedure commences under a slight positive pressure of nitrogen with the external water jacket in current. Eluent mixtures are prepared in a separate 100 mL or 250 mL Schlenk flask under nitrogen by means of volumetric syringes and proportions given herein are therefore only approximate. Collection of bands/fractions from the column is achieved by exchanging receiving Schlenk flasks under stream of nitrogen by careful manipulation of the apparatus – the receiving flask is connected to a three-way tap leading to a supply of nitrogen and exiting to an oil bubbler. Caution – the solid phase for chromatography is likely contaminated with arsenic and should be disposed of appropriately.

2. Computational Details

Computational studies were performed by using the *SPARTAN20*® suite of programs.[3] Geometry optimisation (gas phase) was performed at the DFT level of theory using the exchange functional (ωB97X-D) of Head-Gordon.[4] The Los Alamos effective core potential type basis set (LANL2Dζ) of Hay and Wadt[5] was used for Hg, Pt and Au; the Pople 6-31G* basis sets[6] were used for all other atoms. Geometry optimisations were performed at the ωB97X-D/6-31G*/LANL2Dζ level and frequency calculations were performed to confirm that the optimized structures were minima and to identify vibrational modes of interest.

The singular vibrational scaling factor for the ωBP97X-D/6-31G* combination is given as 0.949 by the National Institute of Standards and Technology (NIST). Thermodynamic properties provided are based on 0.942 as implemented in *Spartan20*. For complexes of the form encountered here, comparison of data in the νCO region of the infrared spectrum with experimentally determined data, a dual scaling factor (ν > 1800 cm^{-1}: 0.9382) based on measured and calculated frequencies for the A' mode of $Co(CO)_2(\eta$-$C_5H_5)$ is more prudent.

2.1 $Co(CO)_2(\eta^5$-$C_5H_4)$ ($2'_{CH}$)

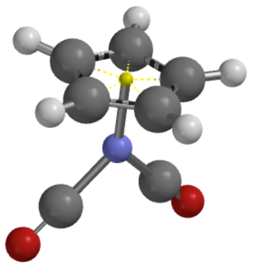

Figure S1. Optimised geometry for **$2'_{CH}$** (ωB97X-D/6-31G*/LANL2Dζ)

Table S1. Cartesian Coordinates for $Co(CO)_2(\eta^5$-$AsC_4H_4)$ (**$2'_{As}$**)

Atom	x	y	z
Co	0.006427	-0.003356	0.828018
H	2.280452	-0.309540	-1.007189
C	1.209109	-0.156541	-0.871347
C	0.534560	1.133077	-0.895257
H	0.412767	-2.280170	-1.082649
C	-0.893015	0.851358	-0.850291
H	-1.684917	1.592318	-0.967668
C	-1.094414	-0.600434	-0.907309
H	-2.053891	-1.097393	-1.058739
C	0.202238	-1.222057	-0.919938
C	-1.128456	0.540352	2.045034
O	-1.897730	0.917878	2.831517
C	1.159815	-0.558812	2.022195
O	1.947431	-0.930486	2.793191
H	0.999102	2.104206	-1.064948

Thermodynamic properties (298.15 K, ωB97X-D/6-31G*/LANL2Dζ)

Zero Point Energy : 254.64 kJmol^{-1} (ZPE)
Temperature Correction: 24.54 kJmol^{-1}
(vibration + gas law +rotation + translation)
Enthalpy Correction: 279.19 kJmol^{-1}
(ZPE + temperature correction)
Enthalpy: -1802.615551 au
(Electronic Energy + Enthalpy Correction)
Entropy : 376.46 Jmol^{-1}K^{-1}

Gibbs Energy: -1802.658301 au (Enthalpy - T*Entropy)
Cv:147.38 Jmol^{-1}K^{-1}

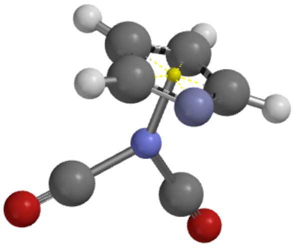

2.2 Co(CO)$_2$(η^5-NC$_4$H$_4$) (2'$_N$)

Figure S2. Optimised geometry for **2'$_N$** (ωB97X-D/6-31G*/LANL2Dζ)

Table S2. Cartesian Coordinates for Co(CO)$_2$(η^5-AsC$_4$H$_4$) (**2'$_N$**)

Atom	x	y	z
Co	-0.108494	0.000000	0.724589
H	-0.971279	-2.088287	-0.924218
C	-0.573783	-1.081874	-0.933800
C	0.803822	-0.702385	-1.021902
H	1.654871	-1.366071	-1.070855
C	0.803822	0.702385	-1.021901
H	1.654871	1.366071	-1.070854
C	-0.573783	1.081873	-0.933800
H	-0.971279	2.088286	-0.924218
N	-1.411079	-0.000000	-0.941710
C	-0.050474	-1.271161	1.904052
O	-0.000862	-2.130848	2.663554
C	-0.050474	1.271161	1.904052
O	-0.000862	2.130848	2.663553

Thermodynamic properties (298.15 K, ωB97X-D/6-31G*/LANL2Dζ)
Zero Point Energy: 225.86 kJmol^{-1} (ZPE)
Temperature Correction: 24.13 kJmol^{-1}
(vibration + gas law + rotation + translation)
Enthalpy Correction: 249.100 kJmol^{-1}
(ZPE + temperature correction)
Enthalpy: -1818.654433 au
(Electronic Energy + Enthalpy Correction)
Entropy: 374.75 Jmol^{-1}K^{-1}
Gibbs Energy: -1818.696990 au (Enthalpy - T*Entropy)
Cv: 142.31 Jmol^{-1}K^{-1}

2.3 Co(CO)$_2$(η^5-PC$_4$H$_4$) (2'$_P$)

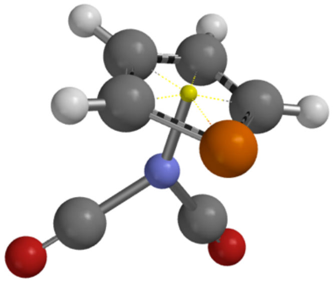

Figure S3. Optimised geometry for **2'$_P$** (ωB97X-D/6-31G*/LANL2Dζ)

Table S3. Cartesian Coordinates for Co(CO)$_2$(η^5-AsC$_4$H$_4$) (**2'$_{As}$**)

Atom	x	y	z
Co	-0.203955	-0.000000	0.678107
H	-0.687785	-2.289088	-0.888868
C	-0.494966	-1.223579	-0.945928
C	0.833261	-0.703799	-0.972040
H	1.725777	-1.317129	-0.956005
C	0.833261	0.703799	-0.972039
H	1.725777	1.317128	-0.956005
C	-0.494966	1.223579	-0.945928
H	-0.687785	2.289088	-0.888868
P	-1.779859	0.000000	-1.044497
C	-0.166645	-1.281756	1.855949
O	-0.144862	-2.152290	2.603444
C	-0.166646	1.281756	1.855949
O	-0.144863	2.152290	2.603444

Thermodynamic properties (298.15 K, ωB97X-D/6-31G*/LANL2Dζ)
Zero Point Energy: 216.36 kJmol^{-1} (ZPE)
Temperature Correction: 25.38 kJmol^{-1}
(vibration + gas law + rotation + translation)
Enthalpy Correction: 241 .74 kJmol^{-1}
(ZPE + temperature correction)
Enthalpy: -2105.272601 au
(Electronic Energy +Enthalpy Correction)
Entropy: 384.19 Jmol^{-1}K^{-1}
Gibbs Energy: -2105.316229 au (Enthalpy - T*Entropy)
Cv: 151.04 Jmol^{-1}K^{-1}

2.4 $Co(CO)_2(\eta^5\text{-}AsC_4H_4)$ (2'$_{As}$)

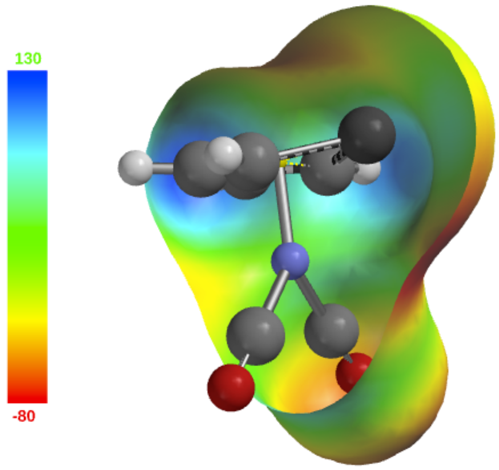

Figure S4. Optimised geometry for **2'**$_{As}$ (ωB97X-D/6-31G*/LANL2Dζ) with electrostatic potential surface.

Table S4. Cartesian Coordinates for $Co(CO)_2(\eta^5\text{-}AsC_4H_4)$ (**2'**$_{As}$)

Atom	x	y	z
Co	-0.312453	-0.000000	0.634260
H	-0.509732	-2.327633	-0.951731
C	-0.346643	-1.256387	-1.001630
C	0.955365	-0.705103	-0.843373
H	1.844237	-1.306180	-0.691193
C	0.955365	0.705103	-0.843373
H	1.844237	1.306180	-0.691193
C	-0.346642	1.256387	-1.001630
H	-0.509732	2.327633	-0.951731
As	-1.731476	0.000000	-1.332927
C	-0.425676	-1.280204	1.808756
O	-0.495720	-2.156279	2.547009
C	-0.425676	1.280204	1.808756
O	-0.495721	2.156279	2.547009

Thermodynamic properties (298.15 K, ωB97X-D/6-31G*/LANL2Dζ)
Zero Point Energy: 213.44 kJmol^{-1} (ZPE)
Temperature Correction: 26.03 kJmol^{-1}
(vibration + gas law + rotation + translation)
Enthalpy Correction: 239.47 kJmol^{-1}
(ZPE + temperature correction)
Enthalpy: -3999.596750 au
(Electronic Energy + Enthalpy Correction)
Entropy: 394.13 Jmol^{-1}K^{-1}
Gibbs Energy: -3999.641507 au (Enthalpy - T*Entropy)
Cv: 154.78 Jmol^{-1}K^{-1}

Table S5. Atomic charges and condensed Fukui indices for $Co(CO)_2(\eta^5\text{-}AsC_4H_4)$ (**2'**$_{As}$) and $[Co(CO)_2(\eta^5\text{-}AsC_4H_4)]^+$ [**2'**$_{As}$]$^+$

Atom	Mulliken	Natural	f^-(Mulliken)	f^- (Natural)
**2'As**				
Co	0.076	0.806		
As	0.142	0.579		
**[2'As]**$^+$				
Co	0.216	0.919	0.140	0.113
As	0.366	0.830	0.224	0.251

2.5 $Co(CO)_2(\eta^5\text{-}SbC_4H_4)$ (2'$_{Sb}$)

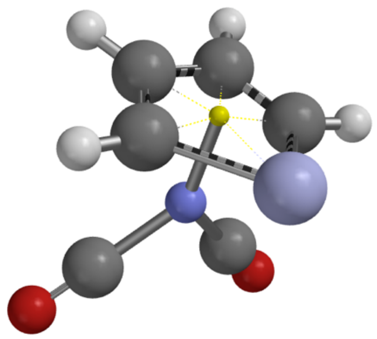

Figure S5. Optimised geometry for **2'**$_{Sb}$ (ωB97X-D/6-31G*/LANL2Dζ)

Table S6. Cartesian Coordinates for $Co(CO)_2(\eta^5\text{-}SbC_4H_4)$ (**2'**$_{Sb}$)

Atom	x	y	z
Co	-0.303012	0.000892	0.623747
H	-0.417529	-2.383759	-0.900860
C	-0.325480	-1.305736	-0.988406
C	0.954492	-0.705879	-0.853919
H	1.859496	-1.282840	-0.695149
C	0.953880	0.707765	-0.854387
H	1.858850	1.284606	-0.694972
C	-0.326598	1.305080	-0.991299
H	-0.422831	2.382845	-0.905798
Sb	-1.918670	-0.001351	-1.399199
C	-0.417470	-1.285372	1.794989
O	-0.497208	-2.159073	2.535243
C	-0.413396	1.285509	1.797281
O	-0.489592	2.157375	2.540010

Thermodynamic properties (298.15 K, ωB97X-D/6-31G*/LANL2Dz)
Zero Point Energy: 211.22 kJmol^{-1} (ZPE)
Temperature Correction: 26.51kJmol^{-1}
(vibration + gas law + rotation + translation)
Enthalpy Correction: 237.73 kJmol^{-1}
(ZPE + temperature correction)
Enthalpy: -1769.337270 au
(Electronic Energy + Enthalpy Correction)
Entropy: 401.29 Jmol^{-1}K^{-1}
Gibbs Energy: -1769.382840 au (Enthalpy - T*Entropy)
Cv: 157.18 Jmol^{-1}K^{-1}

Table S6. Calculated Natural Atomic Charges and Infrared Data[a] for $[Co(CO)_2(\eta^5\text{-}AC_4H_4)]^+$ **2'**$_A$

A =	Charge (A)	Charge (Co)	ν_{CO}(A')	ν_{CO}(A'')	k_{CO}
CH[b]			*2033*	*1972*	*16.17*
CH	-0.374[c]	0.821	2033	1989	16.31
N	-0.517	0.816	2047	2003	16.54
P	0.530	0.807	2048	2009	16.59
As	0.579	0.806	2043	2007	16.54
Sb	0.766	0.796	2041	2007	16.52

[a]ωB97X-D/6-31G*/gas phase; scaling factor 0.9382. [b]Experimental data from cyclohexane solution.[7b] [c]Charge on carbon (charge on H = 0.275).

Observations

(i) The *net* donicity towards cobalt of the η^5-AC_4H_4 ligand is somewhat invariant across the series, as inferred from both the natural charges on cobalt and the ν_{CO} values

(ii) Within the rings, the pyrrolyl ligand is anomalous in that the high electronegativity of nitrogen polarises the C–N bonds resulting in a large negative natural charge on nitrogen and a decrease in the negative charge on the α-carbons (-0.158).

(iii) The significant increase in charge on moving from A = As to A = Sb is noteworthy (Bi is not implemented at this level of theory) making such compounds of interest for isolation and further studies.

Thermodynamic properties (298.15 K, ωB97X-D/6-31G*/LANL2Dz):

Zero Point Energy: 491.30kJmol^{-1} (ZPE)
Temperature Correction: 40.96 kJmol^{-1}
(vibration + gas law + rotation + translation)
Enthalpy Correction: 532.25 kJmol^{-1}
(ZPE + temperature correction)
Enthalpy: -4156.718029 au
(Electronic Energy + Enthalpy Correction)
Entropy: 495.64Jmol^{-1}K^{-1}
Gibbs Energy: -4156.774313 au
(Enthalpy - T*Entropy)
Cv: 258.58Jmol^{-1}K^{-1}

2.6 Co(CO)$_2$(η^5-AsC$_4$Me$_4$) (2c)

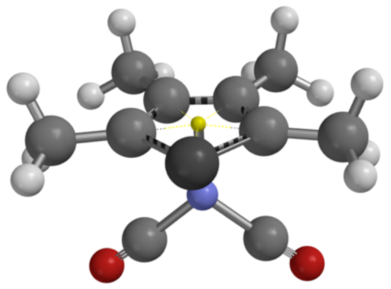

Figure S6. Optimised geometry for **2c** (ωB97X-D/6-31G*/LANL2Dζ)

Table S7. Cartesian Coordinates for Co(CO)$_2$(η^5-AsC$_4$Me$_4$) (**2c**)

Atom	x	y	z
Co	-0.663811	-0.000146	1.094550
C	-0.827312	-1.271121	-0.534552
C	0.486981	-0.711134	-0.477098
C	0.486603	0.711508	-0.477719
C	-0.827615	1.272033	-0.533521
As	-2.219568	0.000483	-0.771228
C	-0.677199	-1.289701	2.260238
O	-0.668757	-2.180739	2.986893
C	-0.677369	1.287597	2.262118
O	-0.669191	2.177699	2.989893
C	-1.078420	2.758028	-0.539355
H	-0.520555	3.282956	0.243474
H	-2.138164	2.981865	-0.390344
H	-0.776911	3.184626	-1.503020
**Atom**	**x**	**y**	**z**
C	1.733156	1.552432	-0.465722
H	2.553262	1.063954	0.065690
H	1.559635	2.518994	0.013584
H	2.065266	1.747178	-1.492769
C	1.733605	-1.552240	-0.461647
H	2.552128	-1.064187	0.072502
H	2.068406	-1.746920	-1.487835
H	1.559086	-2.518937	0.017046
C	-1.078391	-2.757151	-0.539817
H	-0.521926	-3.281733	0.244246
H	-0.775726	-3.184556	-1.502726
H	-2.138414	-2.980777	-0.392362

2.7 [{Co(CO)$_2$(η^5-AsC$_4$H$_4$)}Pt(CO)(CF$_3$)$_2$] (4')

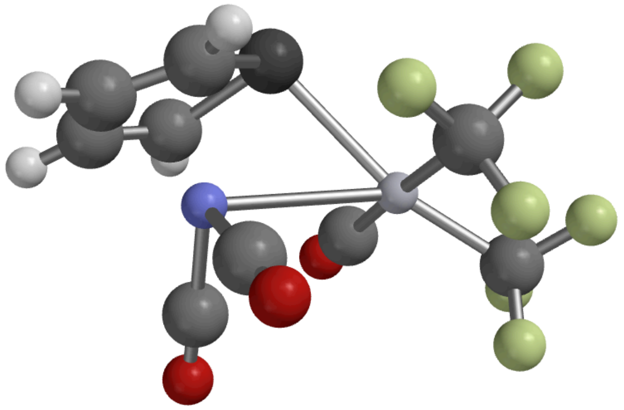

Figure S7. Optimised geometry for **4'** (ωB97X-D/6-31G*/LANL2Dζ)

Table S8. Cartesian Coordinates for [{Co(CO)$_2$(η^5-AsC$_4$H$_4$)}Pt(CO)(CF$_3$)$_2$] (**4'**)

Atom	x	y	z
Pt	-0.616890	-0.152698	1.397446
As	1.507251	1.244471	0.595875
Co	0.674390	-0.192740	-1.253926
O	1.181653	-2.538751	2.183295
O	-1.945699	0.239003	-2.440507
C	2.679830	0.086139	-1.689938
C	0.536144	-1.945969	-1.302839
O	0.485412	-3.087673	-1.338622
C	1.923889	1.175983	-2.190697
C	0.506576	-1.667799	1.893920
C	1.140072	1.800569	-1.202007
C	2.504994	-0.122155	-0.305438
C	-0.931086	0.064899	-1.951633
H	2.976623	-0.958640	0.198943
H	3.283944	-0.555021	-2.322437
H	1.900408	1.450049	-3.239458
H	0.449770	2.600196	-1.434964
C	-1.869697	1.435317	0.995672
C	-2.171320	-0.948002	2.480018
F	-1.441798	2.259601	-0.023462
F	-3.121603	1.084126	0.634784
F	-1.985933	2.255460	2.062994
F	-2.832888	-0.065561	3.245814
F	-3.083576	-1.532698	1.675743
F	-1.750468	-1.928107	3.331423

Thermodynamic properties (298.15 K, ωB97X-D/6-31G*/LANL2Dz)
Zero Point Energy: 306.68 kJmol^{-1} (ZPE)
Temperature Correction : 52.72 kJmol^{-1}
(vibration + gas law + rotation + translation)
Enthalpy Correction: 359.40 kJmol^{-1}
(ZPE + temperature correction)
Enthalpy: -4907.134806 au
(Electronic Energy + Enthalpy Correction)
Entropy: 584.18 Jmol^{-1}K^{-1}
Gibbs Energy: -4907.201145 au (Enthalpy - T*Entropy)
Cv : 342.92 Jmol^{-1}K^{-1}

2.8 [{Co(CO)$_2$(η^5-AsC$_4$Me$_4$)}Pt(CO)(CF$_3$)$_2$] (4”)

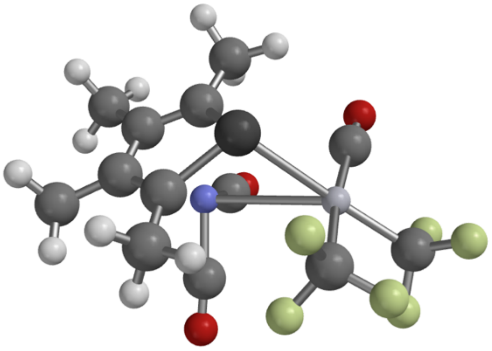

Figure S8. Optimised geometry for **4”** (ωB97X-D/6-31G*/LANL2Dζ)

Table S9. Cartesian Coordinates for [{Co(CO)$_2$(η^5-AsC$_4$Me$_4$)}Pt(CO)(CF$_3$)$_2$] **(4”)**

Atom	x	y	z
Pt	-1.348825	-0.370678	2.130822
As	0.775567	0.961807	1.236657
Co	-0.104981	-0.443168	-0.611271
O	0.143054	-2.989000	2.773076
O	-2.697031	-0.053062	-1.856409
C	1.895095	-0.136212	-1.098496
C	-0.195032	-2.191259	-0.656452
O	-0.182750	-3.335946	-0.706224
C	1.102554	0.962629	-1.556759
C	-0.400101	-2.023238	2.504479
C	0.325561	1.573509	-0.541096
C	1.765674	-0.384386	0.288288
C	-1.699645	-0.212750	-1.323604
C	-2.416075	1.382567	1.938298
C	-3.021931	-1.187441	2.995700
F	-1.609437	2.489894	2.112828
F	-2.963554	1.543905	0.699714
F	-3.423869	1.573090	2.807392
F	-4.135387	-0.958910	2.270591
F	-2.936921	-2.547731	3.098293
F	-3.263648	-0.743791	4.242416
C	2.537850	-1.468600	0.990739
H	3.612405	-1.278710	0.892323
H	2.339124	-2.465089	0.581914
H	2.310234	-1.499629	2.057862
C	1.109933	1.427712	-2.985768
H	1.994920	2.047974	-3.170658
H	0.229005	2.028880	-3.217325
H	1.136689	0.590077	-3.686808
C	-0.541436	2.771903	-0.821381
H	0.090465	3.603063	-1.154742
H	-1.089585	3.089775	0.062041
H	-1.275647	2.572987	-1.608663
C	2.800315	-0.930813	-1.997614
H	3.761589	-0.414760	-2.106005
H	2.375836	-1.061022	-2.995703
H	2.999983	-1.923574	-1.588454

Thermodynamic properties (298.15 K, ωB97X-D/6-31G*/LANL2Dz):
Zero Point Energy: 586.78 kJmol^{-1} (ZPE)
Temperature Correction: 67.38 kJmol^{-1}
(vibration + gas law + rotation + translation)
Enthalpy Correction: 654.16 kJmol^{-1}
(ZPE + temperature correction)
Enthalpy: -5064.262558 au
(Electronic Energy + Enthalpy Correction)
Entropy: 679.22 Jmol^{-1}K^{-1}
Gibbs Energy: -5064.339689 au (Enthalpy - T*Entropy)
Cv: 445.48 Jmol^{-1}K^{-1}

Table S10. Comparison of Infrared Data for [{Co(CO)$_2$(η^5-AsC$_4$H$_4$)}Pt(CO)(CF$_3$)$_2$] **(4’)** and [{Co(CO)$_2$(η^5-AsC$_4$Me$_4$)}Pt(CO)(R)$_2$] (R = C$_6$F$_5$ **4**, CF$_3$ **4”**)

**Complex**		**νCO [cm^{-1}]**		
[{Co(CO)$_2$(η^5-AsC$_4$H$_4$)}Pt(CO)(CF$_3$)$_2$] **4’**	Calculated[a]	2040	2067	2080
	Uncorrected	2174	2204	2218
[{Co(CO)$_2$(η^5-AsCMe$_4$)}Pt(CO)(CF$_3$)$_2$] **4’**	Calculated[a]	2021	2057	2073
	Uncorrected	2155	2193	2210
[{Co(CO)$_2$(η^5-AsC$_4$Me$_4$)}Pt(CO)(C$_6$F$_5$)$_2$] **4**	Measured[b]	2014	2053	2084

[a]ωB97X-D/6-31G*/LANL2Dζ/gas phase, scaling factor 0.9382. [b]CH$_2$Cl$_2$ solution.

Table S11. Comparison of Geometrical Data for [{Co(CO)$_2$(η^5-AsC$_4$H$_4$)}Pt(CO)(CF$_3$)$_2$] **(4’)** and [{Co(CO)$_2$(η^5-AsC$_4$Me$_4$)}Pt(CO)(R)$_2$] (R = C$_6$F$_5$ **4**, CF$_3$ **4”**)

**Complex**	**Co-Pt** [Å]	**Co-As** [Å]	**Pt-As** [Å]	**Pt-C$_{trans}$** [Å]	**Pt-C$_{cis}$** [Å]	**As-Pt-Co** [°]
**4’**[a]	2.949	2.486	2.666	2.054	2.062	52.26
**4”**[a]	3.012	2.483	2.662	2.053	2.062	51.45
**4**[b]	2.996	2.499	2.516	2.077	2.053	53.04

[a]ωB97X-D/6-31G*/LANL2Dζ/gas phase. [b]X-ray diffraction.

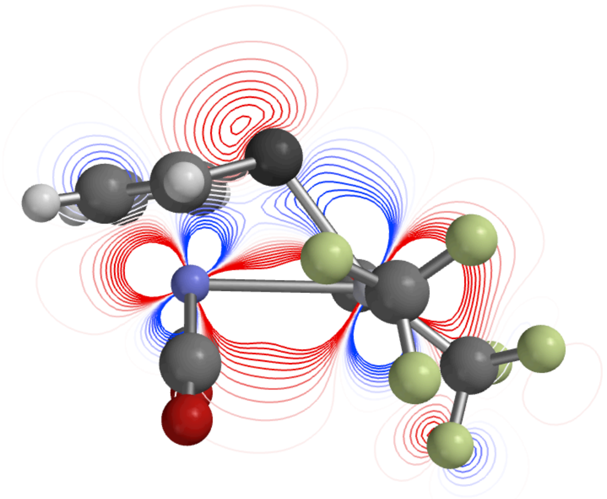

Figure S9. Topology of HOMO-7 for **4’** (ωB97X-D/6-31G*/LANL2Dζ) consistent with Co-Pt σ-bonding.

2.9 [{Co(CO)$_2$(η^5-AsC$_4$H$_4$)}$_2$Au]$^+$ (5’)

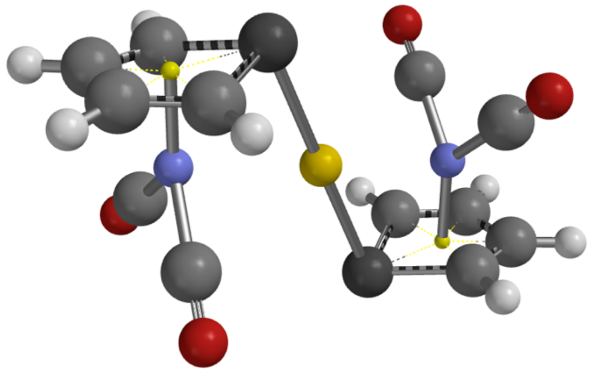

Figure S10. Optimised geometry for **5’** (ωB97X-D/6-31G*/LANL2Dζ)

Table S12. Cartesian Coordinates for [{Co(CO)$_2$(η^5-AsC$_4$H$_4$)}$_2$Au]$^+$ (**5’**)

Atom	x	y	z
Co	-0.187757	-0.034150	2.705172
H	1.366466	-2.394746	2.726482
C	1.406843	-1.322908	2.884705
C	1.116700	-0.747622	4.140426
H	0.844412	-1.325906	5.016223
C	1.142936	0.669357	4.122944
H	0.891654	1.278305	4.984082
C	1.453568	1.202711	2.853946
H	1.451704	2.271086	2.669668
As	2.062209	-0.086685	1.575317
Au	0.001947	-0.059929	0.000097
C	-1.364464	1.283019	2.588486
O	-2.104957	2.147308	2.522648
C	-1.408250	-1.312890	2.618702
O	-2.178330	-2.152188	2.574741
Co	0.190535	0.043653	-2.703914
H	-1.312410	-2.347986	-2.794882
C	-1.375396	-1.273032	-2.921043
C	-1.099030	-0.654809	-4.159289
H	-0.815299	-1.200981	-5.051895
C	-1.156841	0.760111	-4.100318
H	-0.919758	1.399448	-4.943236
C	-1.477495	1.248505	-2.815870
H	-1.499180	2.310720	-2.599892
As	-2.057362	-0.090878	-1.575996
C	1.336712	1.383918	-2.552139
O	2.048454	2.270421	-2.467780
C	1.433469	-1.214250	-2.655753
O	2.208920	-2.049602	-2.641632

Thermodynamic properties (298.15 K, ωB97X-D/6-31G*/LANL2Dz):
Zero Point Energy: 433.25 kJmol^{-1} (ZPE)
Temperature Correction: 53.34 kJmol^{-1}
(vibration + gas law + rotation + translation)
Enthalpy Correction: 486.58 kJmol^{-1}
(ZPE + temperature correction)
Enthalpy: -8134.527779 au
(Electronic Energy + Enthalpy Correction)
Entropy: 590.58 Jmol^{-1}K^{-1}
Gibbs Energy: -8134.594845 au (Enthalpy - T*Entropy)
Cv: 357.97 Jmol^{-1}K^{-1}

2.10 [{Co(CO)$_2$(η^5-AsC$_4$Me$_4$)}$_2$Au]$^+$ (5”)

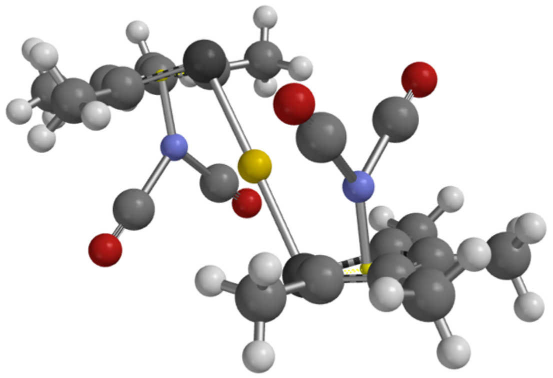

Figure S11. Optimised geometry for **5”** (ωB97X-D/6-31G*/LANL2Dζ)

Table S13. Cartesian Coordinates for [{Co(CO)$_2$(η^5-AsC$_4$Me$_4$)}$_2$Au]$^+$ (**5’**)

Atom	x	y	z
Co	-2.708384	0.257810	-0.049812
C	-2.949408	-1.301648	-1.378892
C	-4.189964	-1.022441	-0.750221
C	-4.128316	-1.075512	0.680411
C	-2.839348	-1.397283	1.177605
As	-1.621957	-1.998491	-0.179445
Au	0.001054	0.006477	-0.148759
C	-2.531481	1.393550	1.286482
O	-2.430486	2.099355	2.179681
C	-2.645814	1.486952	-1.311513
O	-2.625342	2.252957	-2.158920
Co	2.707283	-0.256204	0.035302
C	2.990069	1.276000	-1.315937
C	4.212593	1.005549	-0.648691
C	4.113556	1.085230	0.778708

Atom	x	y	z
C	2.812379	1.418070	1.236580
As	1.634603	2.002028	-0.164978
C	2.482573	-1.363856	1.387694
O	2.345944	-2.050653	2.290933
C	2.675086	-1.510618	-1.202260
O	2.671704	-2.294611	-2.033306
C	5.498921	0.677114	-1.352009
H	6.037383	-0.137312	-0.861462
H	6.150842	1.558178	-1.349246
H	5.332143	0.392604	-2.392114
C	2.819257	1.243767	-2.810250
H	3.315406	0.384893	-3.272120
H	3.244448	2.150633	-3.253577
H	1.762053	1.200685	-3.086024
C	5.285636	0.848595	1.687866
H	5.895713	0.004499	1.358187
H	4.971505	0.656345	2.715048
H	5.924768	1.738922	1.697686
C	2.432922	1.547578	2.686878
H	2.846911	0.743455	3.302661
H	1.346817	1.534004	2.813713
H	2.804949	2.496714	3.087471
C	-2.739125	-1.294736	-2.868376
H	-3.157360	-2.206640	-3.307988
H	-1.674864	-1.261048	-3.116987
H	-3.218381	-0.440640	-3.356519
C	-5.458949	-0.712567	-1.492723
H	-6.111259	-1.593241	-1.481895
H	-5.267410	-0.456360	-2.535887
H	-6.008087	0.114539	-1.036543
C	-5.328386	-0.822101	1.547757
H	-5.978471	-1.704464	1.537144
H	-5.915605	0.028664	1.194050
H	-5.048080	-0.631112	2.584784
C	-2.501637	-1.492101	2.640644
H	-2.887617	-2.430416	3.053334
H	-2.931027	-0.671384	3.223469
H	-1.419640	-1.477091	2.798600

Thermodynamic properties (298.15 K, ωB97X-D/6-31G*/LANL2Dz):

Zero Point Energy: 990.01 kJmol^{-1} (ZPE)
Temperature Correction: 82.94 kJmol^{-1}
(vibration + gas law + rotation + translation)
Enthalpy Correction: 1072.95 kJmol^{-1}
(ZPE + temperature correction)
Enthalpy: -8448.795549 au
(Electronic Energy + Enthalpy Correction)
Entropy: 781.23 Jmol^{-1}K^{-1}
Gibbs Energy: -8448.884265 au (Enthalpy - T*Entropy)
Cv: 556.99 Jmol^{-1}K^{-1}

Table S14. Comparison of Infrared Data for $[\{Co(CO)_2(\eta^5\text{-}AsC_4H_4)\}_2Au]^+$ (**5’**) and $[\{Co(CO)_2(\eta^5\text{-}AsC_4Me_4)\}_2Au]^+$ (**5**)

Complex		ν_{CO} [cm^{-1}]		
$[\{Co(CO)_2(\eta^5\text{-}AsC_4H_4)\}_2Au]^+$ **5’**	Calculated[a]	2086	2059	
	Uncorrected	2223	2195	
$[\{Co(CO)_2(\eta^5\text{-}AsC_4Me_4)\}_2Au]^+$ Me$_8$**5’**	Calculated[a]	2071	2041	
	Uncorrected	2207	2176	
$[\{Co(CO)_2(\eta^5\text{-}AsC_4Me_4)\}_2Au]^+$ **5**	Measured[b]	2062	2051	2009

[a]ωB97X-D/6-31G*/LANL2Dζ/gas phase, scaling factor 0.9382. [b]CH_2Cl_2 solution, as $[Au(C_6F_5)_2]^-$ salt, variance with calculated values may suggest a

Table S15. Comparison of Geometrical Data for $[\{Co(CO)_2(\eta^5\text{-}AsC_4H_4)\}_2Au]^+$ (**5’**) and $[\{Co(CO)_2(\eta^5\text{-}AsC_4Me_4)\}_2Au]^+$ (**5**)

**Complex**		**Co-Au** [Å]	**Co-As** [Å]	**Au-As** [Å]	**As-Au-Co** [°]
5’	Calculated[a]	2.713	2.519	2.594	56.62
5	Measured[b]	2.664	2.520	2.526	58.13

[a]ωB97X-D/6-31G*/LANL2Dζ/gas phase. [b]X-ray diffraction of $[Au(C_6F_5)_2]^-$ salt, mean values for crystallographically unique parameters.

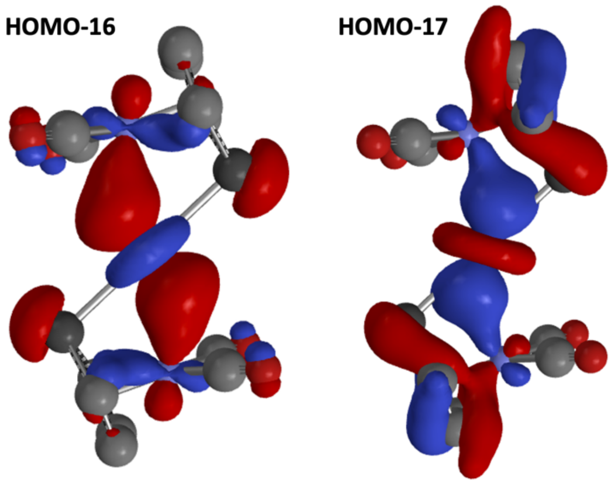

Figure S12. Orbitals associated with Co-Au σ-bonding for **5’**: (a) HOMO-16 (b) HOMO-17,

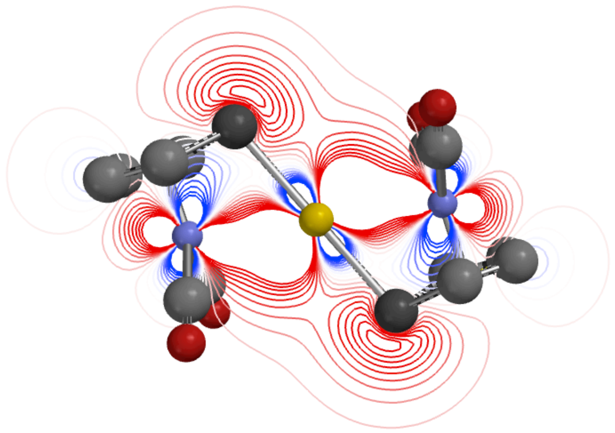

Figure S13. Topology of HOMO-16 (Slice) for **5’** (ωB97X-D/6-31G*/LANL2Dζ) consistent with Co-Au σ-bonding.

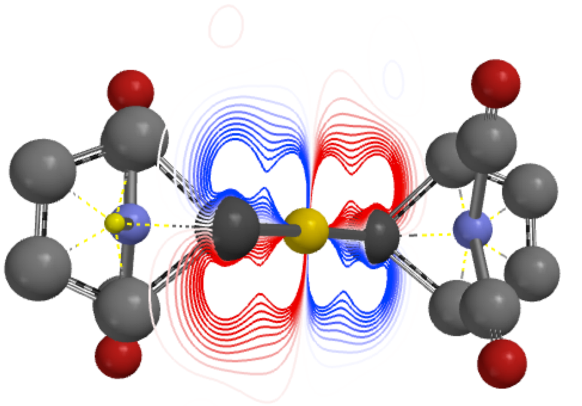

Figure S14. Topology of HOMO-19 (slice) for **5'** (ωB97X-D/6-31G*/LANL2Dζ) consistent with As-Au π-bonding.

Table S16. Calculated Löwden Bond orders

Complex	Co-As	Co-M	As–M
$Co(CO)_2(AsC_4H_4)$ (**2**$_{As}$')	0.702	–	–
$Co(CO)_2(AsC_4Me_4)$ (Me_4-**2**$_{As}$')	0.698		
(**2**$_{As}$')$Pt(CF_3)_2(CO)$ (**4'**)	0.470	0.421	0.736
(Me_4-**2**$_{As}$')$Pt(CF_3)_2(CO)$	0.469	0.383	0.753
(**2**$_{As}$')$_2Au^+$ (**5'**)	0.448	0.559	0.780
(Me_4-**2**$_{As}$')$_2Au^+$	0.454	0.544	0.808

Observations:

(i) In all cases, coordination of the arsenic to an extraneous metal (Au or Pt) results in a decrease in the Co–As bond order;
(ii) Permethylation of the arsolyl ligand results in a negligible of the Co–As bond order for the 'free' metalla-ligands;
(iii) Permethylation of the arsolyl ligand results in a modest (**5'**) or significant (Pt) decrease in the Co–M bond order, most likely due steric impacts.

(a)

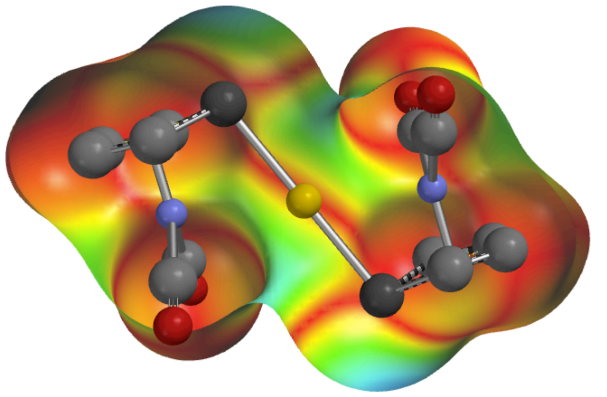

(b)

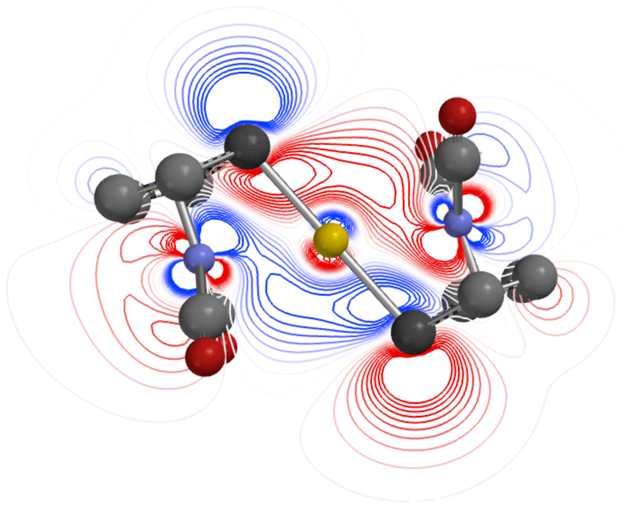

Figure S15. (a) LUMO Map (Isovalue: 0.002 e.au^{-3}) indicating likely interaction sites (As, blue) for $[Au(C_6F_5)_2]^-$ association and (b) LUMO topology (Slice) for **5'**.

3. Additional Tables and Figures

Table S17. Selected spectroscopic data for complexes prepared in this work, and some previously reported complexes for comparison.

Entry	Complex	$^{13}C\ \delta_{C\text{-}\alpha}$[a]	$^{13}C\ \delta_{C\text{-}\beta}$[a]	$^{13}C\ \delta_{CO}$[a]	ν_{CO} (sym)[b]	ν_{CO} (asym)[b]	*k* [c]
**2a**	$Co(CO)_2(\eta^5\text{-}AsC_4Ph_4)$	137.2	135.1	203.8	2026	1978, 1970[d]	16.13
**2b**	$Co(CO)_2(\eta^5\text{-}AsC_4Ph_2H_2)$	138.1	94.9	203.0	2032	1985, 1976[d]	16.23
**2c**	$Co(CO)_2(\eta^5\text{-}AsC_4Me_4)$	118.9[e]	113.1[e]	205.1[e]	2019	1967	16.02
**2d**	$Co(CO)_2(\eta^5\text{-}AsC_4Et_4)$	128.4[e]	118.8[e]	205.3[e]	2017	1965	15.99
**2e**	$Co(CO)_2(\eta^5\text{-}AsC_4TMS_2Me_2)$	122.2[e]	119.8[e]	204.5[e]	2015	1962	15.95
**2f**	$Co(CO)\{P(OMe)_3\}(\eta^5\text{-}AsC_4Me_4)$	114.3	109.8	206.8	1947		15.39
**2g**	$Co(CO)\{P(OMe)_3\}(\eta^5\text{-}AsC_4Et_4)$	124.4	115.3	207.3	1945		15.36
**2h**	$Co(CO)\{P(OMe)_3\}(\eta^5\text{-}AsC_4TMS_2Me_2)$	118.7	116.1	205.5	1941		15.30
Ref.[7]	$Co(CO)_2(\eta^5\text{-}C_5H_5)$	84.5[e]		205.6[e]	2033[f]	1972[f]	16.17
Ref.[7]	$Co(CO)_2(\eta^5\text{-}C_5Me_5)$	96.7[e]		207.9[e]	2011	1949	15.81
Ref.[8]	$Co(CO)_2(\eta^5\text{-}C_5Ph_5)$	*No data reported*			2000	1945	15.69
Ref.[8]	$Co(CO)_2(\eta^5\text{-}C_5\{CH_2Ph\}_5)$	*No data reported*			2020	1960	15.97
Ref.[9]	$Co(CO)_2(\eta^5\text{-}PC_4Ph_2H_2)$	*No data reported*			2030	1980	16.21
Ref.[10]	$Co(CO)_2(\eta^5\text{-}PC_4{}^tBu_2H_2)$	136.2	91.9	204.0	2023	1968	16.06
Ref.[10]	$Co(CO)\{P(OMe)_3\}(\eta^5\text{-}PC_4{}^tBu_2H_2)$	134.7	90.5	208.0	*No data reported*		-

[a] C_6D_6 solution unless otherwise stated, ppm downfield from $SiMe_4$, 25 °C; the labels α and β refer to ring-carbon positions with respect to the heteroatom (where applicable). [b] *n*-hexane solution unless otherwise stated, cm^{-1}, 25 °C. [c] Cotton-Kraihanzel force constant in Ncm^{-1}. [d] resolution of the doubly degenerate *E* vibrational mode is observed in *n*-hexane for these complexes *c.f.*, ν_{CO} (CH_2Cl_2): **2a** 2022, 1963 cm^{-1}; **2b** 2027, 1972 cm^{-1}. [e] $CDCl_3$ solution, 25 °C. [f] Cyclohexane solution.

4. Synthetic Procedures and Spectroscopic and Crystallographic Data

4.1 $Co(CO)_2(\eta^5\text{-}AsC_4Ph_4)$ (2a):

A 250 mL three-necked round bottom flask fitted with a water-cooled reflux condenser was charged 600 mg $Co_2(CO)_8$ (1.75 mmol) and 800 mg $PhAsC_4Ph_4$ (1.5 mmol) or 700 mg $ClAsC_4Ph_4$ (1.5 mmol); 80 mL THF was admitted, and the mixture was brought to reflux under argon for 6 hours. After this time, volatiles were removed under reduced pressure and the residue was take up in CH_2Cl_2, filtered through a short Celite® plug and adsorbed onto a small quantity of oven-dried, degassed silica gel by evaporation of the solvent. This was transferred to a water-jacketed column of oven dried silica gel (30 x 2.5 cm) prepared by loading with *n*-pentane under nitrogen. Eluting with 4:1 *n*-pentane/benzene (**caution – carcinogen**) provided a bright orange band which was collected under nitrogen and taken to dryness. The product was re-crystallised from the minimum amount of hot *n*-hexane/benzene (*ca.* 9:1 *v*/*v*) cooled to -20 °C overnight providing 230 mg of the title compound as an orange-brown crystalline solid in 28 % isolated yield (based on $ClAsC_4Ph_4$; a 20 % yield was obtained with $PhAsC_4Ph_4$). The product is readily soluble in CH_2Cl_2, $CHCl_3$, THF, acetonitrile, acetone, benzene and toluene, somewhat soluble in Et_2O and less-so in *n*-hexane and *n*-pentane. NMR (C_6D_6, 25 °C): ^1H (400 MHz) δ 7.78—7.15 (m, 8H, C_6H_5), 6.85—6.77 (m, 12H, C_6H_5); $^{13}C\{^1H\}$ (176 MHz) δ 203.8 (*C*O), 137.2 (C_α), 135.1 (C_β), 133.1 (C_6H_5), 132.5 (C_6H_5), 130.5 (C_6H_5), 128.2 (C_6H_5), 127.9 (C_6H_5), 127.7 (C_6H_5), 127.6 (C_6H_5), 119.5 (C_6H_5); some aromatic carbon resonances are partially obscured by the C_6D_6 solvent signal, however a $^{13}C\{^1H\}$ spectrum in $CDCl_3$ finds δ 203.2 (*C*O), 136.9 (C_α), 134.6 (C_β), 136.8 (C_6H_5), 134.6 (C_6H_5), 132.2 (C_6H_5), 130.2 (C_6H_5), 127.9 (C_6H_5), 127.6 (C_6H_5), 127.5 (C_6H_5), 127.4

(C_6H_5), 127.3 (C_6H_5), 119.1 (C_6H_5); IR (*n*-hexane) ν_{CO} = 2026(vs), 1978(vs), 1970(vs) cm^{-1}; IR (CH_2Cl_2) ν_{CO} = 2022(vs), 1963(vs) cm^{-1}; HR-MS (EI, MeCN) found *m*/*z* 546.0014 (calc. for $C_{30}H_{20}O_2{}^{75}As^{59}Co$ [*M*]$^+$: 546.0011). Analysis found: C, 65.91; H, 3.61%; calc. for $C_{30}H_{20}O_2AsCo$: C, 65.95; H, 3.69%.

Single crystals of **2a** suitable for X-ray diffraction were grown from benzene/*n*-hexane at -20 °C. A suitable crystal was selected and mounted in oil on a Micromount™ mylar loop and fixed under a cold stream of nitrogen. Data were collected using an Agilent Technologies Supernova/EosS2-CCD diffractometer with graphite monochromated Cu-K_α radiation (λ = 1.54184 Å) at 150.0(1) K. Data were processed using the CrysAlisPRO-CCD and –RED software packages[11] and a Gaussian absorption correction was applied (T_{min} / T_{max} = 0.219 / 1.000). The structure was solved within Olex2[12] with SHELXT[13] using Intrinsic Phasing and refined with the SHELXL[14] refinement package using Least-Squares against F^2 in an anisotropic (for non-hydrogen atoms) approximation. Aromatic hydrogen atom positions were refined in isotropic approximation in a "riding" model with the U_{iso}(H) parameters fixed to 1.2 U_{eq}(C), where U_{eq}(C) is the equivalent thermal parameter of the carbon atom to which the corresponding H atom is bonded. Principal crystallographic data and refinement parameters are as follows: $C_{30}H_{20}AsCoO_2$ (M_w = 546.31 gmol^{-1}): orange plate 0.298 × 0.110 × 0.083 mm, triclinic, space group *P*-1 (no. 2), *a* = 9.0514(4) Å, *b* = 10.5907(5) Å, *c* = 12.8357(6) Å, α = 76.116(4)°, β = 84.418(4)°, γ = 78.076(4)°, *V* = 1167.24(10) Å^3, *Z* = 2, μ(Cu-K_α) = 7.541 mm^{-1}, ρ_{calc} = 1.554 Mgm^{-3}, 11946 reflections measured (7.104° ≤ 2Θ ≤ 147.544°), 4691 unique (R_{int} = 0.0239, R_{sigma} = 0.0282) which were used in all calculations, GOF = 1.037, D_{min} / D_{max} = -0.64 / 0.44 e/Å^3. The final R_1 was 0.0336 ($I > 2\sigma(I)$) and wR_2 was 0.0887 (all data). CCDC number 2130783.

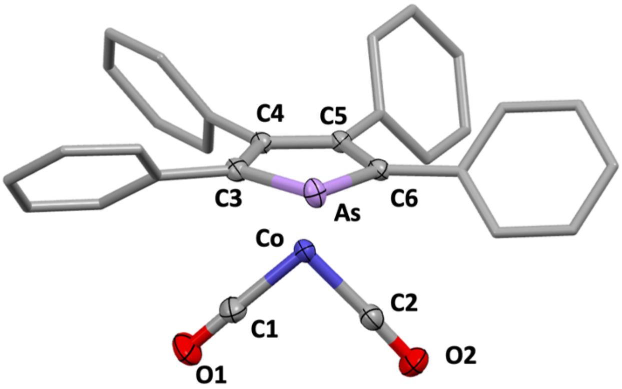

Figure S16. Molecular structure of **2a** (50% displacement ellipsoids, arsenolyl ring substituents simplified). Selected distances [Å] and angles [°]:As1-Co1 2.427(4), As1-C3 1.900(2), C3-C4 1.443(3), C4-C5 1.427(3), C5-C6 1.439(3), C6-As1 1.915(2), Co1-C1 1.746(3), Co1-C2 1.747(3), C3-As1-C6 84.52(8), As1-C3-C4 114.32(1), C3-C4-C5 113.65(2), C4-C5-C6 112.84(2), C5-C6-As1 114.38(1), C1-Co1-C2 90.82(1).

4.2 $Co(CO)_2(\eta^5\text{-}AsC_4Ph_2H_2)$ (2b):

Prepared as above. Quantities: 500 mg $Co_2(CO)_8$ (1.5 mmol), 450 mg $PhAsC_4P_2H_2$ (1.25 mmol). Chromatographed on silica gel (30 x 2.5 cm) under nitrogen with 9:1 *n*-pentane/benzene providing a bright orange band. Re-crystallised from the minimum amount of hot *n*-hexane/benzene (ca. 9:1 *v*/*v*) cooled to -20 °C overnight, isolated yield of orange-brown crystalline solid: 105 mg (21 % based on arsenic). Solubility properties are similar to **2a** above. NMR (C_6D_6, 25 °C) ^{1}H (400 MHz) δ 7.27 (m, 4H, C_6H_5), 6.99 (m, 6H, C_6H_5) 5.93 (s, 2H, β-C*H*); ^{13}C{^{1}H} (176 MHz) δ 203.2 (*C*O), 138.1 (C_α), 128.9 (C_6H_5), 128.5 (C_6H_5), 128.3 (C_6H_5), 127.3 (C_6H_5), 94.9 (C_β); some aromatic carbon resonances are partially obscured by the C_6D_6 solvent signal, however a ^{13}C{^{1}H} spectrum in $CDCl_3$ finds δ 202.7 (*C*O), 137.9 (C_α), 128.9 (C_6H_5), 128.8 (C_6H_5) 128.3 (C_6H_5), 127.2 (C_6H_5), 94.7 (C_β); IR (*n*-hexane) ν_{CO} = 2032(vs), 1985(vs), 1976(vs) cm^{-1}; IR (CH_2Cl_2) ν_{CO} = 2027(vs), 1974(vs) cm^{-1}; HR-MS (EI, MeCN) found *m*/*z* 393.9383 (calc. for $C_{18}H_{12}O_2{}^{75}As^{59}Co$ [*M*]$^+$: 393.9385). Analysis found: C, 54.86; H, 2.93%; calc. for $C_{18}H_{12}O_2AsCo$: C, 54.85; H, 3.07%.

Single crystals of **2b** suitable for X-ray diffraction were grown from benzene/*n*-hexane at -20 °C. A suitable crystal was selected and mounted in oil on a Micromount™ mylar loop and fixed under a cold stream of nitrogen. Data were collected using an Agilent Technologies Supernova/EosS2-CCD diffractometer with graphite monochromated Cu-K_α radiation (λ = 1.54184 Å) at 150.0(1) K. Data were processed using the CrysAlisPRO-CCD and –RED software packages[11] and Multi-scan absorption correction was applied (T_{min} / T_{max} = 0.56452 / 1.00000). The structure was solved within Olex2[12] with SHELXT[13] using Intrinsic Phasing and refined with the SHELXL[14] refinement package using Least-Squares against F^2 in an anisotropic (for non-hydrogen atoms) approximation. Aromatic hydrogen atom positions were refined in isotropic approximation in a "riding" model with the U_{iso}(H) parameters fixed to 1.2 U_{eq}(C), where U_{eq}(C) is the equivalent thermal parameter of the carbon atom to which the corresponding H atom is bonded. Atom C5 (see Fig. S2) exhibited a belligerent thermal ellipsoid which could be indicative of some rotational disorder of the η^5-arsolyl group however this could not be modelled satisfactory – this effect was present across three separate crystal specimens and did not appear to affect data quality. Principal crystallographic data and refinement parameters are as follows: $C_{18}H_{12}AsCoO_2$ (M_w = 394.13 gmol^{-1}): orange needle, 0.109 × 0.061 × 0.029 mm, orthorhombic, space group *Pna*2$_1$ (no. 33), *a* = 11.23470(10) Å, *b* = 17.27710(10) Å, *c* = 7.83560(10) Å, *V* = 1520.91(3) Å^3, *Z* = 4, *μ*(Cu-K_α) = 11.289 mm^{-1}, ρ_{calc} = 1.721 Mgm^{-3}, 27398 reflections measured (9.39° ≤ 2Θ ≤ 147.908°), 3057 unique (R_{int} = 0.0453, R_{sigma} = 0.0224) which were used in all calculations, GOF = 1.085, D_{min}/D_{max} = -0.65/0.97 eÅ^{-3}. The final R_1 was 0.0386 ($I > 2\sigma(I)$) and wR_2 was 0.0936 (all data) for 193 parameters and one restraint. CCDC number 2130784.

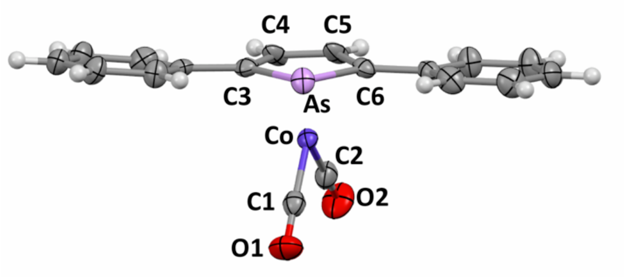

Figure S17. Molecular structures of **2b** (50% displacement ellipsoids, arsenolyl ring substituents simplified). Selected distances [Å] and angles [°]: As1-Co1 2.393(1), As1-C3 1.933(7), C3-C4 1.389(9), C4-C5 1.365(1), C5-C6 1.357(9), C6-As1 1.915(6), Co1-C1 1.735(8), Co1-C2 1.734(7), C3-As1-C6 87.37(3), As1-C3-C4 111.12(5), C3-C4-C5 110.23(6), C4-C5-C6 126.02(6), C5-C6-As1 104.64(5), C1-Co1-C2 94.45(3).

4.3 Co(CO)$_2$(η^5-AsC$_4$Me$_4$) (2c):

Prepared as above with the modification of heating to reflux in 50 mL *n*-hexane for 6 hours. Typical quantities: 720 mg $Co_2(CO)_8$ (2.1 mmol) and 430 mg freshly prepared ClAsC$_4$Me$_4$ (2 mmol). Chromatography on neutral alumina (25 x 2.5 cm) under nitrogen with *n*-pentane provided an orange-red band, which was collected under nitrogen and taken to dryness providing between *ca*. 80—190 mg of the product as a dark orange-red liquid in an average isolated yield of ~19 % (based on ClAsC$_4$Me$_4$ across 6 separate preparations; a ~15 % average isolated yield was obtained with PhAsC$_4$Me$_4$ across 3 separate preparations). The product is readily soluble most common organic solvents, though was difficult to completely free from solvents with which it had previously been dissolved despite being held under reduced pressure for extended periods of time, as evidenced by NMR spectroscopy. The product is readily soluble in most common organic solvents. NMR (CDCl$_3$, 25 °C) ^{1}H (400 MHz) δ 2.12 (s, 6H, α-C*H*$_3$), 1.85 (s, 6H, β-C*H*$_3$); ^{13}C{^{1}H} (176 MHz) δ 205.8 (*C*O), 118.9 (*C*$_α$), 113.1 (*C*$_β$), 17.1 (α-*C*H$_3$), 14.5 (β-*C*H$_3$); IR (*n*-hexane) $ν_{CO}$ = 2019(vs), 1967(vs) cm^{-1}; HR-MS (EI, MeCN) found *m*/*z* 297.9389 (calc. for C$_{10}$H$_{12}$O$_4$^{75}As^{59}Co [*M*]$^+$: 297.9385); satisfactory elemental microanalysis could not be obtained due to entrained solvent.

4.4 Co(CO)$_2$(η^5-AsC$_4$Et$_4$) (2d):

Prepared as above. Quantities: 480 mg $Co_2(CO)_8$ (1.4 mmol) and 350 mg ClAsC$_4$Et$_4$, (1.25 mmol). Chromatography on neutral alumina (25 x 2.5 cm) under nitrogen with *n*-pentane provided an orange-red band, which was collected under nitrogen and taken to dryness under reduced pressure giving 105—130 mg of the product as a dark orange-red liquid in an average isolated yield of 25 % (based on arsenic from 2 separate preparations; a 16 % yield was obtained with PhAsC$_4$Et$_4$ from one preparation). The product is readily soluble in most common organic solvents. NMR (CDCl$_3$, 25 °C) ^{1}H (400 MHz; NB: diastereotpic methylene protons on each Et groups) δ 2.54 (dt, *J* = 15, 7, 7 Hz, 2H, α-C*H*$_2$CH$_3$), 2.41 (dt, *J* = 15, 7, 7 Hz, 2H, α-C*H*$_2$CH$_3$), 2.26 (dt, *J* = 15, 7, 7 Hz, 2H, β-CH$_2$C*H*$_3$), 2.07 (dt, *J* = 15, 7, 7 Hz, 2H, β-CH$_2$C*H*$_3$), 1.19 (td, *J* = 7, 7, 3 Hz, 12H, α- & β-CH$_2$C*H*$_3$); ^{13}C{^{1}H} (176 MHz) δ 205.3 (*C*O), 128.4 (*C*$_α$), 118.8 (*C*$_β$), 24.4 (α-*C*H$_2$CH$_3$), 21.2 (β-*C*H$_2$CH$_3$), 16.9 (α-CH$_2$*C*H$_3$), 16.6 (β-CH$_2$*C*H$_3$); these data are consistent with methyl groups *syn* or *anti* to the cobalt not interconverting on the ^{13}C NMR timescale. IR (*n*-hexane) $ν_{CO}$ = 2017(vs), 1965(vs) cm^{-1}; HR-MS (EI, MeCN) found *m*/*z* 354.0011 (calc. for C$_{14}$H$_{20}$O$_2$^{59}Co$_1$^{75}As$_1$ [*M*]$^+$: 354.0010); Satisfactory elemental microanalytical data were not be obtained for this oil.

4.5 Co(CO)$_2$(η^5-AsC$_4$(SiMe$_3$)$_2$Me$_2$) (2e):

Prepared as above. Quantities: 450 mg $Co_2(CO)_8$ (1.3 mmol) and 400 mg ClAsC$_4$TMS$_2$Me$_2$ (1.2 mmol) Chromatography on alumina (25 x 2.5 cm) with *n*-pentane provides an orange band which is collected under nitrogen and taken to dryness, giving 130 mg of the product as a dark orange liquid in 26 % isolated yield (based on arsenic). The product is readily soluble in most common organic solvents. NMR (CDCl$_3$, 25 °C) ^{1}H (400 MHz) δ 2.21 (s, 6H, β-C*H*$_3$), 0.23 (s, 18H, α-Si(C*H*$_3$)$_3$); ^{13}C{^{1}H} (176 MHz) δ 204.5 (*C*O), 122.2 (*C*$_α$), 119.8 (*C*$_β$), 18.4 (β-*C*H$_3$), 1.3 (α-Si(*C*H$_3$)$_3$); IR (*n*-hexane) $ν_{CO}$ = 2015(vs), 1963(vs) cm^{-1}; HR-MS (EI, MeCN) found *m*/*z* 413.9851 (calc. for C$_{14}$H$_{24}$O$_2$^{28}Si$_2$^{59}Co^{75}As [*M*]$^+$: 413.9863). Satisfactory elemental microanalytical data were not be obtained for this oil.

4.6-8 Co(CO){P(OMe)$_3$}(η^5-AsC$_4$R$_4$) (2f—h)

To a solution of **2c** (120 mg, 0.30 mmol), **2d** (110 mg, 0.31 mmol) or **2e** (110 mg, 0.27 mmol) in 10 mL toluene was added ~0.1 mL neat P(OMe)$_3$ (excess) and the mixture warmed to 100 °C – the dark orange-red colour discharged to a bright orange over the course of *ca.* 30 minutes, and heating was continued for a total of 2 hours. After this time, the mixture was transferred *via* syringe to a column of oven-dried neutral alumina (20 x 2.5 cm) made up in *n*-pentane under nitrogen. Eluting with *n*-pentane, followed by 4:1 *n*-pentane/Et$_2$O provided a bright orange band which was collected under nitrogen and taken to dryness under reduced pressure. No unreacted starting materials were observed during chromatography although a minor green-brown band remined immobile atop the alumina. The complexes **2f** and **2h** were obtained as bright orange solids after re-crystallisation from SiMe$_4$ whereas **2g** is an orange liquid which eventually solidified upon prolonged storage at -30 °C. Isolated yields: **2f** 100 mg (85 %), **2g** 110 mg (80 %), and **2h** 115 mg (83 %). All were found to decompose upon contact with air, either neat or in solution, and appear to react with CH$_2$Cl$_2$ and CDCl$_3$ to precipitate unknown paramagnetic materials. All are readily soluble in common organic solvents.

4.6 Co(CO){P(OMe)$_3$}(η^5-AsC$_4$Me$_4$) (2f)

2f: NMR (C$_6$D$_6$, 25 °C): ^{1}H (400 MHz) δ 3.36 (d, $^3J_{PH}$ = 12 Hz, 9H, POC*H*$_3$), 1.94 (s, 6H, α-C*H*$_3$), 1.83 (s, 6H, β-C*H*$_3$); ^{13}C{^{1}H} (176 MHz) δ 206.8 (*C*O), 114.3 (*C*$_α$), 109.8 (*C*$_β$), 51.0 (PO*C*H$_3$), 16.9 (α-*C*H$_3$), 14.4 (β-*C*H$_3$); ^{31}P{^{1}H} (161 MHz) δ 3.3 (s, broad); IR (*n*-hexane) $ν_{CO}$ = 1947 cm^{-1}; HR-MS (EI, MeCN) found m/z 393.9725

(calc. for $C_{12}H_{21}O_4{}^{75}As^{59}CoP$ [*M*]$^+$: 393.9725). Analysis found: C, 36.60; H, 5.16%; calc. for $C_{12}H_{21}O_4AsCoP$: 36.57 C, 5.37 H%.

Single crystals of **2f** suitable for X-ray diffraction were grown by evaporation of $SiMe_4$ solution at -30 °C. A suitable crystal was selected and mounted in oil on a Micromount™ mylar loop and fixed under a cold stream of nitrogen. Data were collected using an Agilent Technologies Supernova/EosS2-CCD diffractometer with graphite monochromated Cu-K_α radiation (λ = 1.54184 Å) at 150.0(1) K. Data were processed using the CrysAlisPRO-CCD and –RED software packages[11] and a Gaussian absorption correction was applied (T_{min} / T_{max} = 0.29678 / 0.37651). The structure was solved within Olex2[12] with SHELXT[13] using Intrinsic Phasing and refined with SHELXL[14] refinement package using Least-Squares against F^2 in an anisotropic (for non-hydrogen atoms) approximation. Aliphatic hydrogen atom positions were refined in isotropic approximation in a "riding" model with the U_{iso}(H) parameters fixed to 1.5 U_{eq}(C), where U_{eq}(C) is the equivalent thermal parameter of the carbon atom to which the corresponding H atom is bonded. Two independent molecules of **2f** were found in the unit cell. Principal crystallographic data and refinement parameters are as follows: $C_{12}H_{21}AsCoO_4P$ (M_w = 394.11 gmol^{-1}): orange plate, 0.235 × 0.176 × 0.075 mm, monoclinic, space group $P2_1/n$ (no. 14), *a* = 8.19800(10) Å, *b* = 14.8986(2) Å, *c* = 26.2475(3) Å, β = 97.0710(10)°, *V* = 3181.45(7) Å^3, *Z* = 8, μ(Cu-K_α) = 11.788 mm^{-1}, ρ_{calc} = 1.646 Mgm^{-3}, 42721 reflections measured (6.786° ≤ 2Θ ≤ 148.738°), 6425 unique (R_{int} = 0.0382, R_{sigma} = 0.0223) which were used in all calculations, GOF = 1.065, D_{min} / D_{max} = -0.89 / 1.46 eÅ^{-3}. The final R_1 was 0.0360 ($I > 2\sigma(I)$) and wR_2 was 0.0875 (all data). CCDC number 2130785.

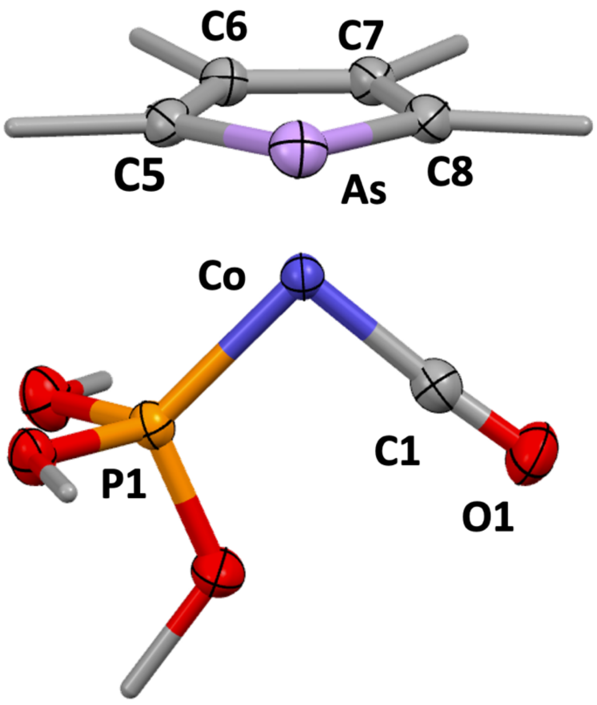

Figure S18 Molecular structure of **2f** (50% displacement ellipsoids, arsenolyl ring and phosphite substituents simplified). Selected distances [Å] and angles [°]: As1-Co1 2.408(5), As1-C5 1.910(3), C5-C6 1.430(4), C6-C7 1.423(4), C7-C8 1.427(5), C8-As1 1.903(3), Co1-C1 1.719(3), Co1-P1 2.097(9), C5-As1-C8 84.12(1), As1-C5-C6 114.25(2), C5-C6-C7 113.30(3), C6-C7-C8 113.47(3), C7-C8-As1 114.56(2), C1-Co1-P1 93.49(1).

4.7 Co(CO){P(OMe)$_3$}(η^5-AsC$_4$Et$_4$) (2g)

2g: NMR (C_6D_6, 25 °C): ^{1}H (400 MHz) δ 3.40 (d, $^3J_{PH}$ = 12 Hz, 9H, POCH_3), 2.45 (m, 4H, α-CH_2CH$_3$), 2.19 (m, 4H, β-CH_2CH$_3$), 1.23 (t, $^3J_{HH}$ = 7 Hz, 6H, α-CH_3), 1.13 (t, $^3J_{HH}$ = 7 Hz, 6H, β-CH_3); ^{13}C{^{1}H} (176 MHz) δ 207.4 (*C*O), 124.4 (C_α), 115.3 (C_β), 51.3 (PO*C*H$_3$), 24.7 (α-*C*H$_2$CH$_3$), 22.0 (β-*C*H$_2$CH$_3$), 17.8 (α-CH$_2$*C*H$_3$), 16.6 (β-CH$_2$*C*H$_3$); ^{31}P{^{1}H} (161 MHz) δ 3.4 (s, broad); IR (*n*-hexane) ν_{CO} = 1945 cm^{-1}; HR-MS (EI, MeCN) found *m*/*z* 450.0351 (calc. for $C_{16}H_{29}O_4{}^{75}AsCoP$ [*M*]$^+$: 450.0352). Analysis found: C, 42.73; H, 6.31%; calc. for $C_{16}H_{29}O_4AsCoP$: C, 42.68; H, 6.49%. Single crystals suitable for X-ray diffraction were not obtained.

4.8 Co(CO){P(OMe)$_3$}(η^5-AsC$_4$(SiMe$_3$)$_2$Me$_2$) (2h)

2h: NMR (C_6D_6, 25 °C): ^{1}H (400 MHz) δ 3.40 (d, $^3J_{PH}$ = 12 Hz, 9H, POCH_3), 2.15 (s, 6H, β-CH_3), 0.32 (s, 18H, α-Si(CH_3)$_3$); ^{13}C{^{1}H} (176 MHz) δ 205.6 (*C*O), 118.7 (C_α), 116.0 (C_β), 51.5 (PO*C*H$_3$), 18.7 (β-*C*H$_3$), 1.6 (Si(*C*H$_3$)$_3$); ^{31}P{^{1}H} (161 MHz) δ 3.8 (s, broad); IR (*n*-hexane) ν_{CO} = 1941 cm^{-1}; HR-MS (EI, MeCN) found *m*/*z* 510.0199 (calc. for $C_{16}H_{33}O_4{}^{75}AsCo_1P_1{}^{29}Si_2$ [*M*]$^+$: 510.0203). Analysis found: C, 37.55; H, 6.32%; calc. for $C_{16}H_{33}O_4AsCoPSi_2$: C, 37.65; H, 6.52%.

Single crystals of **2h** suitable for X-ray diffraction were grown by evaporation of $SiMe_4$ solution at -30 °C. A suitable crystal was selected and mounted in oil on a Micromount™ mylar loop and fixed under a cold stream of nitrogen. Data were collected using an Agilent Technologies Supernova/EosS2-CCD diffractometer with graphite monochromated Cu-K_α radiation (λ = 1.54184 Å) at 150.0(1) K. Data were processed using the CrysAlisPRO-CCD and –RED software packages[11] and a Gaussian absorption correction was applied (T_{min} / T_{max} = 0.178 / 0.672). The structure was solved within Olex2[12] with SHELXT[13] using Intrinsic Phasing and refined with the SHELXL[14] refinement package using Least-Squares against F^2 in an anisotropic (for non-hydrogen atoms) approximation. Aliphatic hydrogen atom positions were refined in isotropic approximation in a "riding" model with the U_{iso}(H) parameters fixed to 1.5 U_{eq}(C), where U_{eq}(C) is the equivalent thermal parameter of the carbon atom to which the corresponding H atom is bonded. Principal crystallographic data and refinement parameters are as follows: $C_{16}H_{33}AsCoO_4PSi_2$ (M_w = 510.42 gmol^{-1}): orange needle, 0.554 × 0.091 × 0.042 mm, monoclinic, space group *Cc* (no. 9), *a* = 7.93850(10) Å, *b* = 19.9045(2) Å, *c* = 15.36370(10) Å, β = 90.3000(10)°, *V* = 2427.61(4) Å^3, *Z* = 4, μ(Cu-K_α) = 8.764 mm^{-1}, ρ_{calc} = 1.397 Mgm^{-3}, 23360 reflections measured (8.886° ≤ 2Θ ≤ 147.518°), 4232 unique (R_{int} = 0.0252, R_{sigma} = 0.0181) which were used in all calculations, GOF = 1.030, D_{min} / D_{max} = -0.32 / 0.31 eÅ^{-3}.. The final R_1 was 0.0219 (I > 2σ(I)) and wR_2 was 0.0564 (all data). CCDC number 2130786.

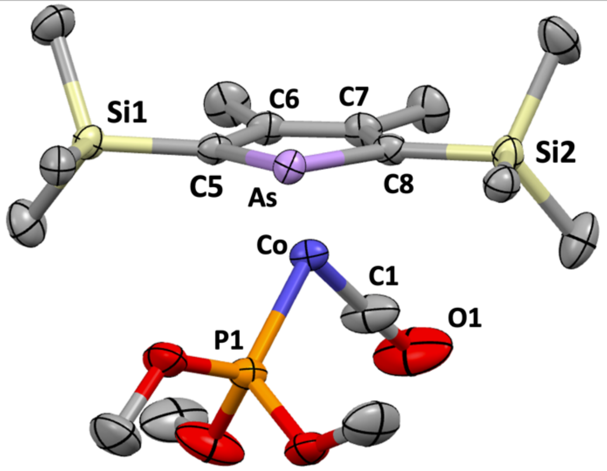

Figure S19. Molecular structure **2h** (50% displacement ellipsoids, arsolyl ring and phosphite substituents simplified). Selected distances [Å] and angles [°]: As1-Co1 2.3957(6), As1-C5 1.942(3), C5-C6 1.408(4), C6-C7 1.451(4), C7-C8 1.424(4), C8-As1 1.893(3), Co1-C1 1.712(4), Co1-P1 2.094(1), C5-As1-C8 87.48(1), As1-C5-C6 111.19(2), C5-C6-C7 114.09(3), C6-C7-C8 116.06(3), C7-C8-As1 111.06(2), C1-Co1-P1 91.14(2).

4.9 [Co(CO)$_2$(σ:η^5-AsC$_4$Me$_4$)·HgCl(μ-Cl)]$_2$ (3)

To a stirred solution of **2c** (150 mg, 0.50 mmol) in 3 mL acetone was added a solution of $HgCl_2$ (125 mg, 0.46 mmol) in 3 mL of the same solvent. After a brief while a bright yellow precipitate began to form, and the mixture was stirred for a further 60 minutes. After this time, the almost colourless supernatant was decanted, and the solids washed further with acetone (3 x 3 mL portions) and Et_2O (3 x 3 mL portions) and dried under vacuum. The isolated yield was 230 mg (91 % based on Hg). The product may be handled in air for some time as a solid however gains a brown cast overnight. The product was poorly soluble in virtually all available solvents and only partially dissolved in acetonitrile with prolonged trituration. Satisfactory NMR spectra could, regrettably, not be obtained due to extensive precipitation before or during acquisition. As indicated by IR, the bulk sample obtained from this preparation is exclusively the *anti*-isomer. IR (MeCN) ν_{CO} = 2072(vs), 2036(vs) cm^{-1}; HR-MS (ESI, MeCN) found *m*/*z* 1104.7222 (calc. for $C_{20}H_{24}O_4{}^{75}As_2{}^{35}Cl_3Co_2{}^{202}Hg_2$ [*M* – Cl]$^+$: 1104.7216). Analysis found: C, 21.11; H, 1.98%; calc. for $C_{20}H_{24}O_4As_2Cl_4Co_2Hg_2$: C, 21.09; H, 2.12%.

Single crystals of ***anti*-3** and ***syn*-3** suitable for X-ray diffraction were grown by layering an acetone solution of **2c** with an acetone solution of $HgCl_2$ at -30 °C. Suitable crystals were selected and mounted in oil on Micromount™ mylar loops and fixed under a cold stream of nitrogen. Data were collected using an Agilent Technologies Supernova/EosS2-CCD diffractometer with graphite monochromated Cu-K$_\alpha$ radiation (λ = 1.54184 Å) at 150.00(10) K. Data were processed using the CrysAlisPRO-CCD and –RED software packages[11] and spherical absorption corrections were applied (***anti*-3**: T_{min} / T_{max} = 0.07271 / 0.17197. ***syn*-3**: T_{min} / T_{max} = 0.02226 / 0.10591). The structures were solved within Olex2[12] with SHELXT[13] using Intrinsic Phasing and refined with the SHELXL[14] refinement package using Least-Squares against F^2 in an anisotropic (for non-hydrogen atoms) approximation. Aliphatic hydrogen atom positions were refined in isotropic approximation in a "riding" model with the U_{iso}(H) parameters fixed to 1.5 U_{eq}(C), where U_{eq}(C) is the equivalent thermal parameter of the carbon atom to which the corresponding H atom is bonded. Principal crystallographic data and refinement parameters are as follows: *anti*-$C_{20}H_{24}As_2Cl_4Co_2Hg_2O_4$ (M_w = 1139.07 gmol^{-1}): yellow needle, 0.51 × 0.074 × 0.046 mm, monoclinic, space group $P2_1/n$ (no. 14), *a* = 8.83040(10) Å, *b* = 14.2969(2) Å, *c* = 11.8460(2) Å, β = 94.2220(10)°, *V* = 1491.47(4) Å^3, *Z* = 2, *μ*(Cu-K$_\alpha$) = 32.581 mm^{-1}, ρ_{calc} = 2.536 Mgm^{-3}, 8025 reflections measured (9.712° ≤ 2Θ ≤ 147.534°), 3000 unique (R_{int} = 0.0307, R_{sigma} = 0.0342) which were used in all calculations, GOF = 1.074, D_{min} / D_{max} = -1.34 / 1.32 e/Å^3. The final R_1 was 0.0415 (I > 2σ(I)) and wR_2 was 0.1021 (all data). CCDC number 2130789; *syn*-$C_{20}H_{24}As_2Cl_4Co_2Hg_2O_4$ (M_w = 1139.07 gmol^{-1}): orange prism, 0.426 × 0.151 × 0.118 mm, monoclinic, space group *C*2/*c* (no. 15), *a* = 14.1928(3) Å, *b* = 14.4745(3) Å, *c* = 14.7169(3) Å, β = 99.340(2)°, *V* = 2983.26(11) Å^3, *Z* = 4, *μ*(Cu-K$_\alpha$) = 32.578 mm^{-1}, ρ_{calc} = 2.536 Mgm^{-3}, 8233 reflections measured (8.786° ≤ 2Θ ≤ 147.722°), 3020 unique (R_{int} = 0.0468, R_{sigma} = 0.0454) which were used in all calculations, GOF = 1.109, D_{min} / D_{max} = -2.61 / 4.25 eÅ^{-3}. The final R_1 was 0.0660 (I > 2σ(I)) and wR_2 was 0.1782 (all data). CCDC number 2130788.

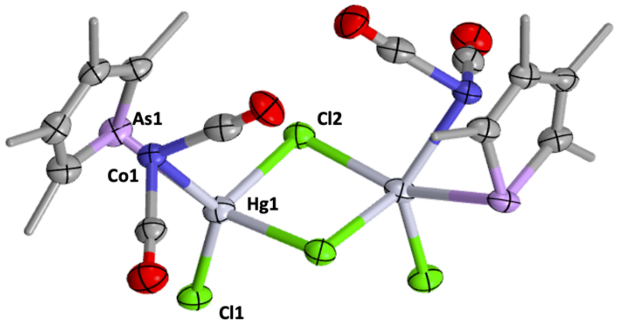

Figure S20. The molecular structure of *syn*-**3** (Methyl groups simplified, 50% displacement ellipsoids). Selected distances [Å] and angles [°]: (a) *syn*-**3** As1-Hg1 2.7268(9), Co1-Hg1 2.620(1), Hg1-Cl1 2.491(2), Hg1-Cl2 2.598(2), Hg1-Cl2' 2.708(2), As1-Hg1-Co1 55.86(3), Hg1-Cl2-Hg1' 91.87(6), Cl2-Hg1-Cl2' 87.62(6), Cl1-Hg1-Cl2 100.71(6).

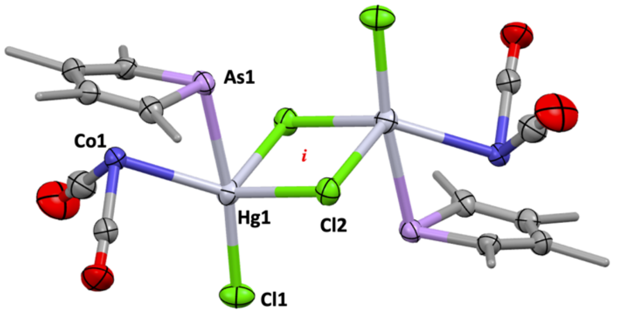

Figure S21. The molecular structures of *anti*-**3** (Methyl groups simplified, 50% displacement ellipsoids). Selected distances [Å] and angles [°]:As1-Hg1 2.6334(6), Co1-Hg1 2.6702(9), Hg1-Cl1 2.390(1), Hg1-Cl2 2.778(1), Hg1-Cl2' 2.777(1) As1-Hg1-Co1 56.27(2), Hg1-Cl2-Hg1' 84.37(4), Cl2-Hg1-Cl2' 95.63(4), Cl1-Hg1-Cl2, 100.59(5). *i* = crystallographic inversion centre.

4.10 *cis*-[{Co(CO)$_2$(μ:σ,η^5-AsC$_4$Me$_4$)}Pt(CO)(C$_6$F$_5$)$_2$] (4)

To a solution of **2c** (110 mg, 0.37 mmol) in 5 mL CH_2Cl_2 was added solid $Pt(C_6F_5)_2(\eta^4\text{-}C_6H_{10})$ (145 mg, 0.27 mmol). Over the course of about 60 minutes the orange colour discharged to a dark yellow-brown; the reaction was monitored by IR and was deemed complete after a further 60 minutes when **2c** was no longer observed. Volatiles were removed under reduced pressure and the residue extracted with the minimum amount of Et_2O and transferred *via* syringe to a water-jacketed column of oven-dried Florisil® (15 x 1 cm) made up in *n*-pentane under nitrogen. Eluting with *n*-pentane, followed by 3:1 *n*-pentane/Et_2O provided an orange band which was collected under nitrogen and taken to dryness under reduced pressure. The orange residue was crystallised from the minimum amount of benzene overnight giving 95 mg of the product as a dark orange solid in 45% yield (based on Pt). The product is readily soluble in CH_2Cl_2, $CHCl_3$, acetone, acetonitrile, Et_2O and THF, somewhat soluble in benzene and toluene and less-so in *n*-hexane or *n*-pentane. NMR (CD_2Cl_2, 25 °C): ^{1}H (400 MHz) δ 2.30 (s, 6H: α-C*H*$_3$), 1.48 (s, 6H: β-C*H*$_3$); ^{13}C{^{1}H} (176 MHz) δ 198.0 (Co-*C*O), 177.17 (Pt-*C*O), 146.7 (m, *C*$_6$F$_5$), 139.6 (m, *C*$_6$F$_5$), 138.7 (m, *C*$_6$F$_5$), 138.2 (m, *C*$_6$F$_5$), 137.0 (m, *C*$_6$F$_5$), 126.2 (C_α), 124.3 (C_β), 17.3 (α-*C*H$_3$), 15.1 (β-*C*H$_3$); ^{19}F{^{1}H} (376 MHz) δ_F -112.47 [d+dd, 2 F, $F^{2,6}(C_6F_5)$, $^3J_{FF}$ = 23, $^3J_{PtF}$ = 358 Hz], -119.77 [d+dd, 2 F, $F^{2,6}(C_6F_5)$, $^3J_{FF}$ = 23, $^3J_{PtF}$ = 354 Hz], -159.87 [t, 1 F, $F^4(C_6F_5)$, $^3J_{FF}$ = 19 Hz], -160.44 [t, 1 F, $F^4(C_6F_5)$, $^3J_{FF}$ = 19 Hz], -163.58 [t, 2 F, $F^{3,5}(C_6F_5)$, $^3J_{FF}$ ~ 25 Hz, 2nd order], -163.96 [t, 2 F, $F^{3,5}(C_6F_5)$, $^3J_{FF}$ ~ 17 Hz, 2nd order]; ^{195}Pt{^{1}H} (150 MHz) δ -4079.8 ppm (t.t.t.t. $A_2B_2C_2D_2X$ approximating to A_4B_4X p.p. as $^3J_{AX}$ ~ $^3J_{BX}$ >> $^4J_{CX}$ ~ $^4J_{DX}$. $^3J_{FPt}$ ~ 344, $^4J_{FPt}$ ~ 83 Hz). IR (CH_2Cl_2) ν_{CO} = 2084(vs), 2053(vs), 2014(vs) cm^{-1}. HR-MS (ESI, MeCN) found *m/z* 798.8942 (calc. for $C_{21}H_{12}O_1{}^{75}AsCoF_{10}{}^{195}Pt$ [*M* – 2CO]$^+$: 798.8936). Analysis found: C, 32.41; H, 1.35%; calc. for $C_{23}H_{12}O_3AsCoF_{10}Pt$: C, 32.30; H, 1.41%.

Single crystals of **4** suitable for X-ray diffraction were grown from evaporation of Et_2O solution at -30 °C. A suitable crystal was selected and mounted in oil on a Micromount™ mylar loop and fixed under a cold stream of nitrogen. Data were collected using an Agilent Technologies Supernova/EosS2-CCD diffractometer with graphite monochromated Cu-K_α radiation (λ = 1.54184 Å) at 150.0(1) K. Data were processed using the CrysAlisPRO-CCD and –RED software packages[11] and a spherical absorption correction was applied (T_{min} / T_{max} = 0.14128 / 0.23775). The structure was solved within Olex2[12] with SHELXT[13] using Intrinsic Phasing and refined with the SHELXL[14] refinement package using Least-Squares against F^2 in an anisotropic (for non-hydrogen atoms) approximation. Aliphatic hydrogen atom positions were refined in isotropic approximation in a "riding" model with the U_{iso}(H) parameters fixed to 1.5 U_{eq}(C), where U_{eq}(C) is the equivalent thermal parameter of the carbon atom to which the corresponding H atom is bonded. The crystal comprised a single enantiomer Principal crystallographic data and refinement parameters are as follows: $C_{23}H_{12}AsCoF_{10}O_3Pt$ (M_w = 855.27 gmol^{-1}): orange prism, 0.264 × 0.12 × 0.067 mm, orthorhombic, space group $P2_12_12_1$ (no. 19), a = 9.18910(10) Å, b = 12.75590(10) Å, c = 21.0227(2) Å, V = 2464.18(4) Å^3, Z = 4, μ(Cu Kα) = 18.190 mm^{-1}, ρ_{calc} = 2.305 Mgm^{-3}, 8725 reflections measured (8.108° ≤ 2Θ ≤ 147.49°), 4597 unique (R_{int} = 0.0233, R_{sigma} = 0.0328) which were used in all calculations, GOF = 1.056, D_{min} / D_{max} = -1.50 / 1.21 eÅ^{-3}. The final R_1 was 0.0292 ($I > 2\sigma(I)$) and wR_2 was 0.0714 (all data). CCDC number 2130787.

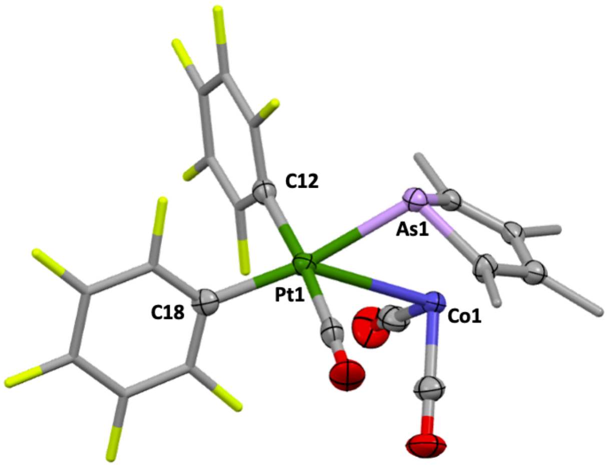

Figure S22: (a) The molecular structure of **5** (Methyl and pentafluorophenyl groups simplified, 50% displacement ellipsoids, Pt *TBPY-5*-12-*C* enantiomer in non-centrosymmetric $P2_12_12_1$ space group). Selected distances [Å] and angles [°]: As1-Pt1 2.5185(8), Co1-Pt1 2.996(1), Pt1-C3 1.889(9), Pt1-C12 2.053(8), Pt-C18 2.077(7), As1-Pt1-Co1 53.04(3), As1-Pt1-C18 169.58(2), Co1-Pt1-C18 137.24(2).

4.11 [{Co(CO)$_2$(μ:σ,η^5-AsC$_4$Me$_4$)}$_2$Au][Au(C$_6$F$_5$)$_2$] (5)

To a stirred solution of **2c** (110 mg, 0.37 mmol) in CH_2Cl_2 (5 mL) was added solid $Au(C_6F_5)(THT)$ (150 mg, 0.33 mmol). After stirring overnight, the mixture was filtered through a short (2 x 2 cm) Celite® plug and the filtrate dried under reduced pressure. The yellow solids obtained were then washed with Et_2O (3 x 3 mL portions). Re-crystallisation from the minimum amount of CH_2Cl_2/Et_2O at -20 °C overnight provided 180 mg of the product as a yellow microcrystalline solid in 84 % isolated yield (based on Au). The product is readily soluble in polar organic solvents and may be handled in air as a solid or solution for a short while however turns brown overnight. NMR (CD_2Cl_2, 25 °C): ^{1}H (400 MHz) δ 2.33 (s, 6H, α-C*H*$_3$), 1.70 (s, 6H, β-C*H*$_3$); ^{13}C{^{1}H} (176 MHz) δ 200.2 (*C*O), 149.5 (m, *C*$_6$F$_5$), 138.7 (m, *C*$_6$F$_5$), 138.3 (m, *C*$_6$F$_5$), 137.35 (m, *C*$_6$F$_5$), 122.7 (C_α), 118.0 (C_β), 18.2 (α-*C*H$_3$), 14.4 (β-*C*H$_3$); ^{19}F{^{1}H} δ -115.63 [d, 2 F, $^3J_{FF}$ = 30 Hz $F^{2,6}(C_6F_5)$], -163.00 (t, 1 F, $^3J_{FF}$ = 21 Hz $F^4(C_6F_5)$], -164.16 [dd, 2 F, $^3J_{FF}$ ~ 23 Hz, $F^{3,5}(C_6F_5)$]; IR (CH_2Cl_2) ν_{CO} = 2062 (sh), 2051(vs), 2009(vs) cm^{-1}; HR-MS (ESI, MeCN) found *m/z* 792.8439 (calc. for $C_{20}H_{24}O_4{}^{75}As_2{}^{197}AuCo_2$ [*M*]$^+$: 792.8436). Analysis found: C, 29.03; H, 1.87%; calc. for $C_{32}H_{24}O_4As_2Au_2Co_2F_{10}$: C, 29.03; H, 1.83%.

Single crystals of **5** suitable for X-ray diffraction were grown from the vapour diffusion of Et_2O into CH_2Cl_2 solution at -30 °C. A suitable crystal was selected and mounted in oil on a Micromount™ mylar loop and fixed under a cold stream of nitrogen. Data were collected using an Agilent Technologies Supernova/EosS2-CCD diffractometer with graphite monochromated Cu-K_α radiation (λ = 1.54184 Å) at 150.0(1) K. Data were processed using the CrysAlisPRO-CCD and –RED software packages[11] and a spherical absorption correction was applied (T_{min} / T_{max} = 0.10891 / 0.20750). The structure was solved within Olex2[12] with SHELXT[13] using Intrinsic Phasing and refined with the SHELXL[14] refinement package using Least-Squares against F^2 in an anisotropic (for non-hydrogen atoms)

approximation. Aliphatic hydrogen atom positions were refined in isotropic approximation in a “riding” model with the U_{iso}(H) parameters fixed to 1.5 U_{eq}(C), where U_{eq}(C) is the equivalent thermal parameter of the carbon atom to which the corresponding H atom is bonded. Principal crystallographic data and refinement parameters are as follows: $C_{32}H_{24}O_4As_2Au_2Co_2F_{10}O_4$ (M_w = 1324.14 gmol^{-1}): yellow needle, 0.346 × 0.085 × 0.08 mm, triclinic, space group *P*-1 (no. 2), *a* = 15.12080(10) Å, *b* = 21.7675(2) Å, *c* = 23.8537(2) Å, α = 100.2830(10)°, β = 104.7800(10)°, γ = 91.0780(10)°, *V* = 7452.43(11) Å^3, *Z* = 8, μ(Cu-K$_α$) = 24.075 mm^{-1}, $ρ_{calc}$ = 2.360 Mgm^{-3}, 149532 reflections measured (7.102° ≤ 2Θ ≤ 146.294°), 29642 unique (R_{int} = 0.0391, R_{sigma} = 0.0298) which were used in all calculations, GOF = 1.041, D_{min} / D_{max} = -1.82 / 1.22 e/Å^3. The final R_1 was 0.0265 ($I > 2σ(I)$) and wR_2 was 0.0631 (all data). CCDC number 2130790.

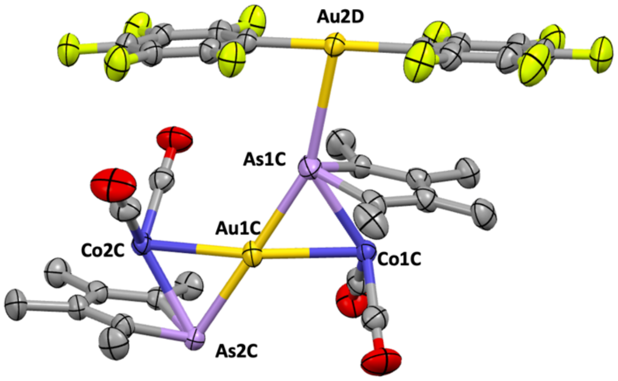

Figure S23. Molecular structure of the repeating unit of infinite chains of **5$_n$** (Hydrogen atoms omitted for clarity, four crystallographically independent molecules in the unit cell, 50% displacement ellipsoids).

5. Selected spectra

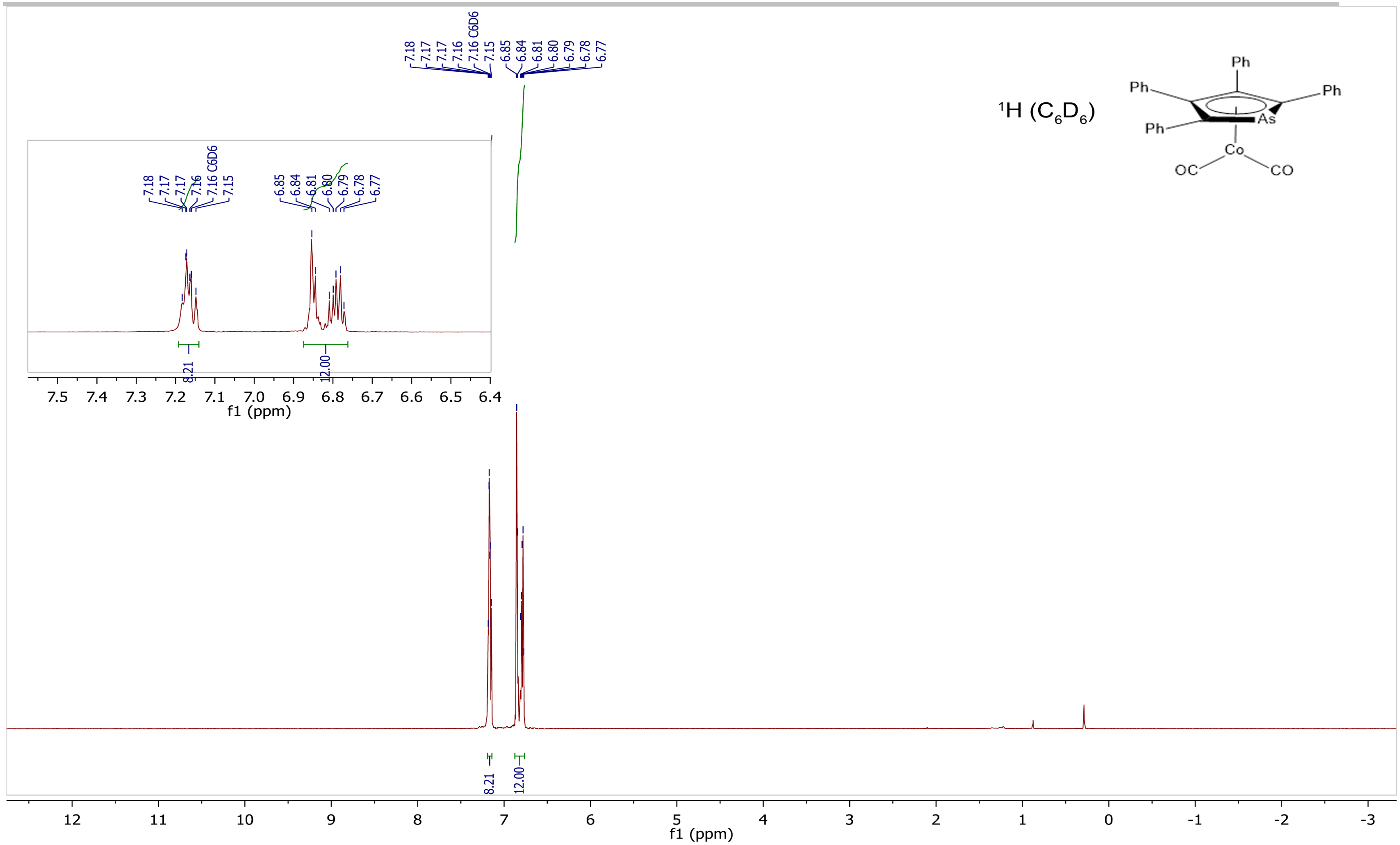

7.18
7.17
7.17
7.16
7.16 C6D6
7.15
6.85
6.84
6.81
6.80
6.79
6.78
6.77
^{1}H (C_6D_6)
Ph
Ph
Ph
Ph
As
Co
OC
CO
8.21
12.00
7.5 7.4 7.3 7.2 7.1 7.0 6.9 6.8 6.7 6.6 6.5 6.4
f1 (ppm)
12 11 10 9 8 7 6 5 4 3 2 1 0 -1 -2 -3
f1 (ppm)

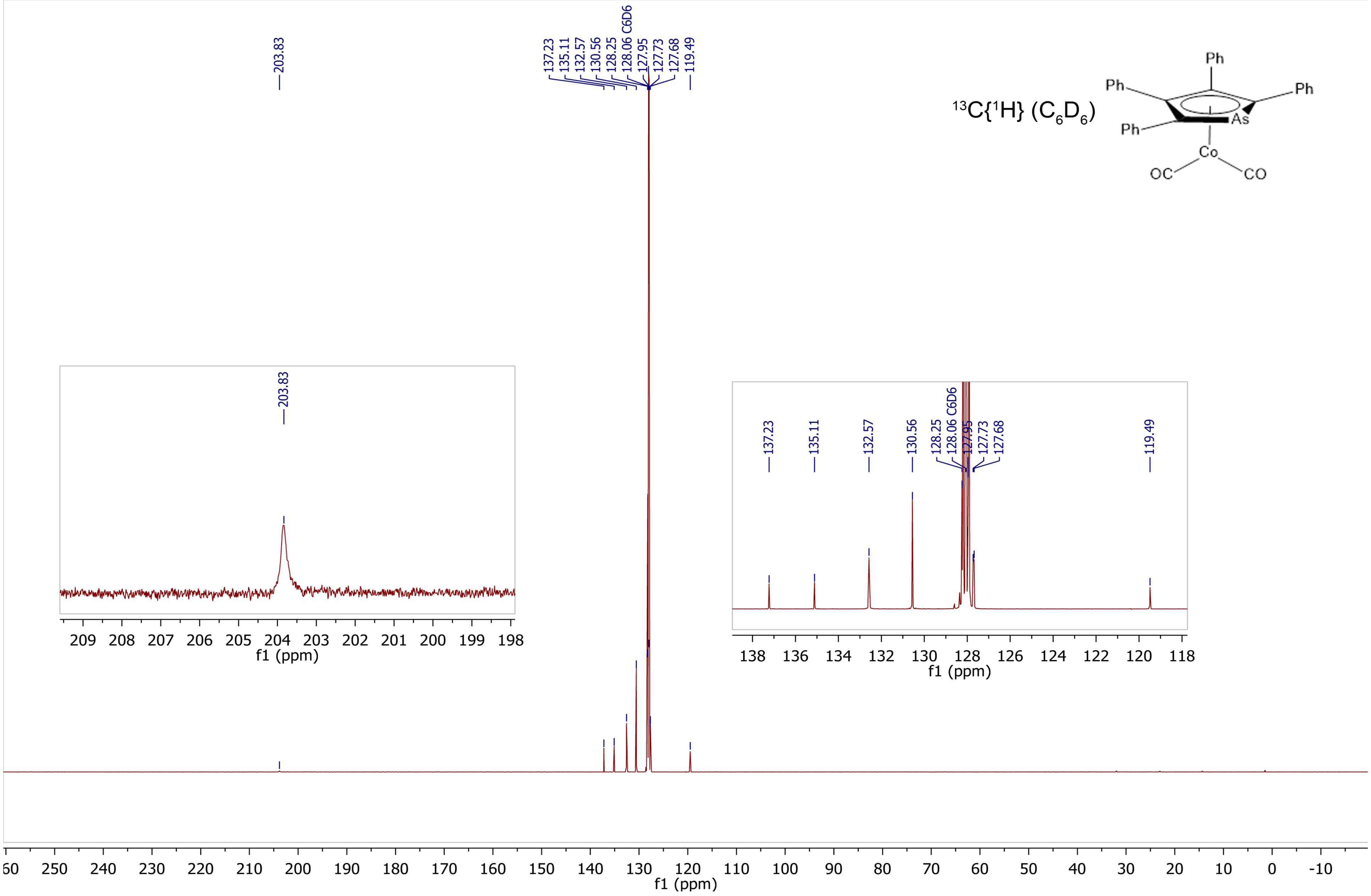

203.83
137.23
135.11
132.57
130.56
128.25
128.06 C6D6
127.95
127.73
127.68
119.49
13C{1H} (C6D6)
Ph
Ph
Ph
Ph
As
Co
OC
CO
f1 (ppm)

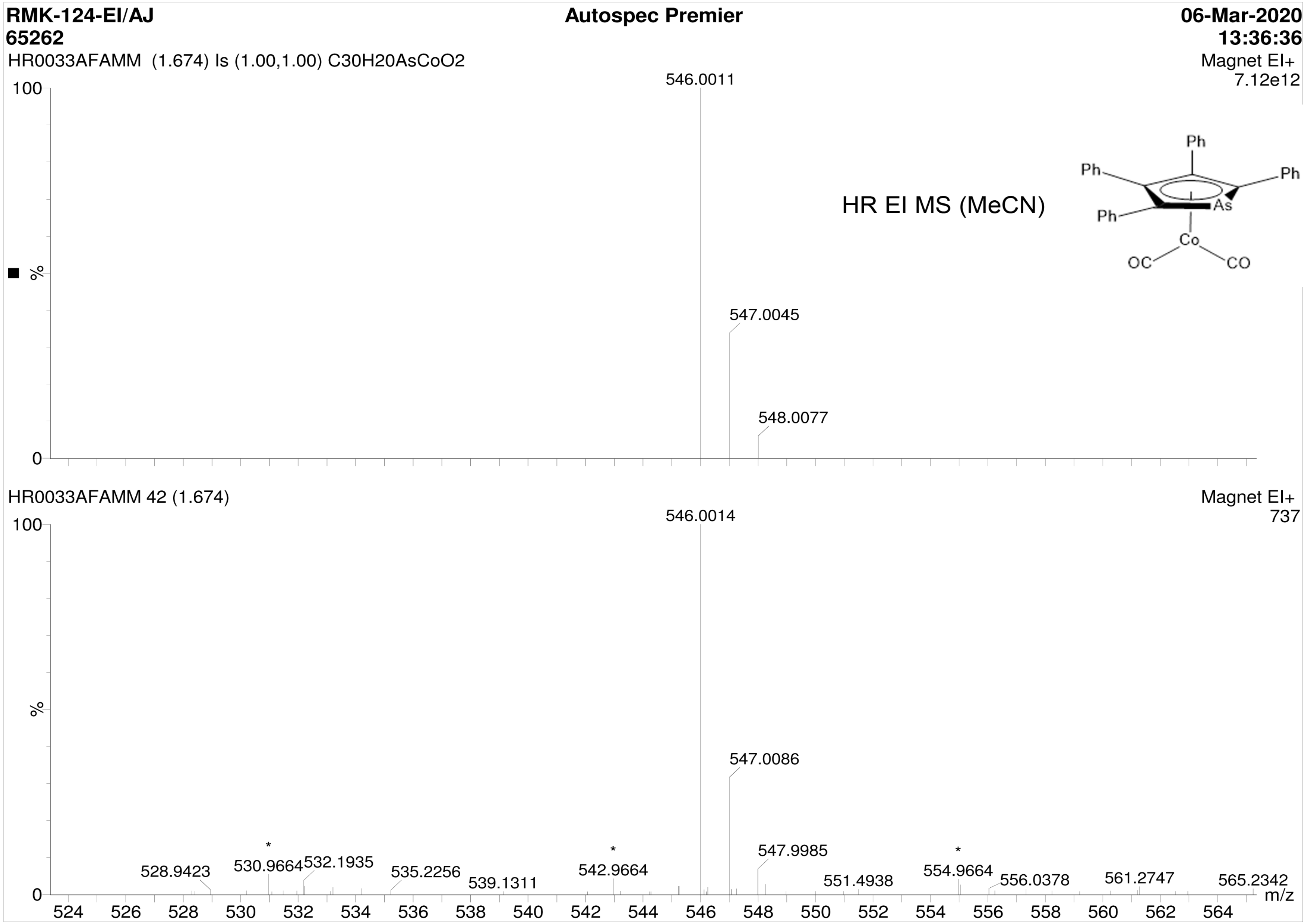

RMK-124-EI/AJ
65262
Autospec Premier
06-Mar-2020
13:36:36
HR0033AFAMM (1.674) Is (1.00,1.00) C30H20AsCoO2
Magnet EI+
7.12e12
546.0011
547.0045
548.0077
HR EI MS (MeCN)
Ph
Ph
Ph
Ph
As
Co
OC
CO
HR0033AFAMM 42 (1.674)
Magnet EI+
737
546.0014
547.0086
547.9985
528.9423
530.9664
532.1935
535.2256
539.1311
542.9664
551.4938
554.9664
556.0378
561.2747
565.2342
%
m/z

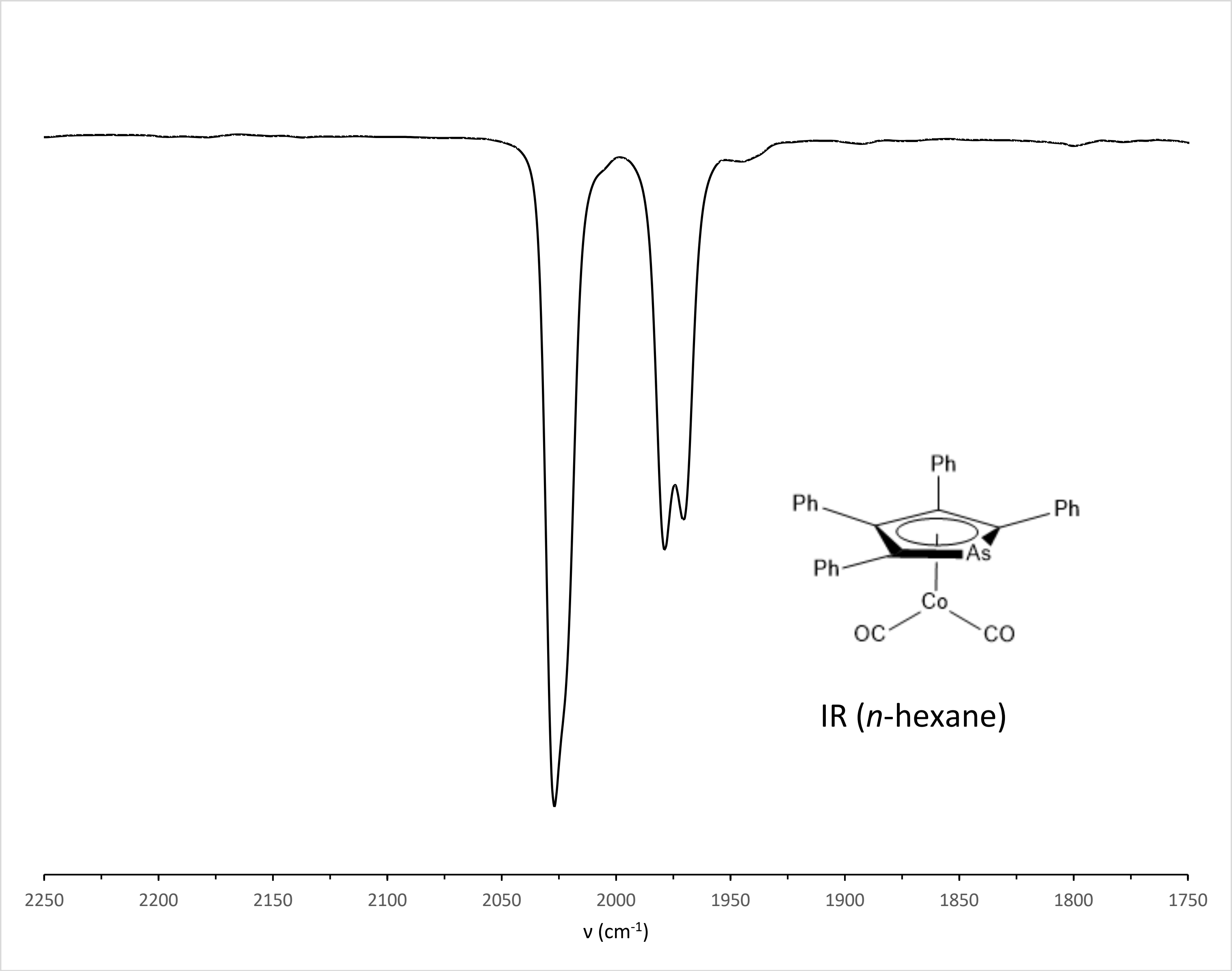
Ph
Ph
Ph
Ph
As
Co
OC
CO
IR (*n*-hexane)
2250
2200
2150
2100
2050
2000
1950
1900
1850
1800
1750
ν (cm⁻¹)

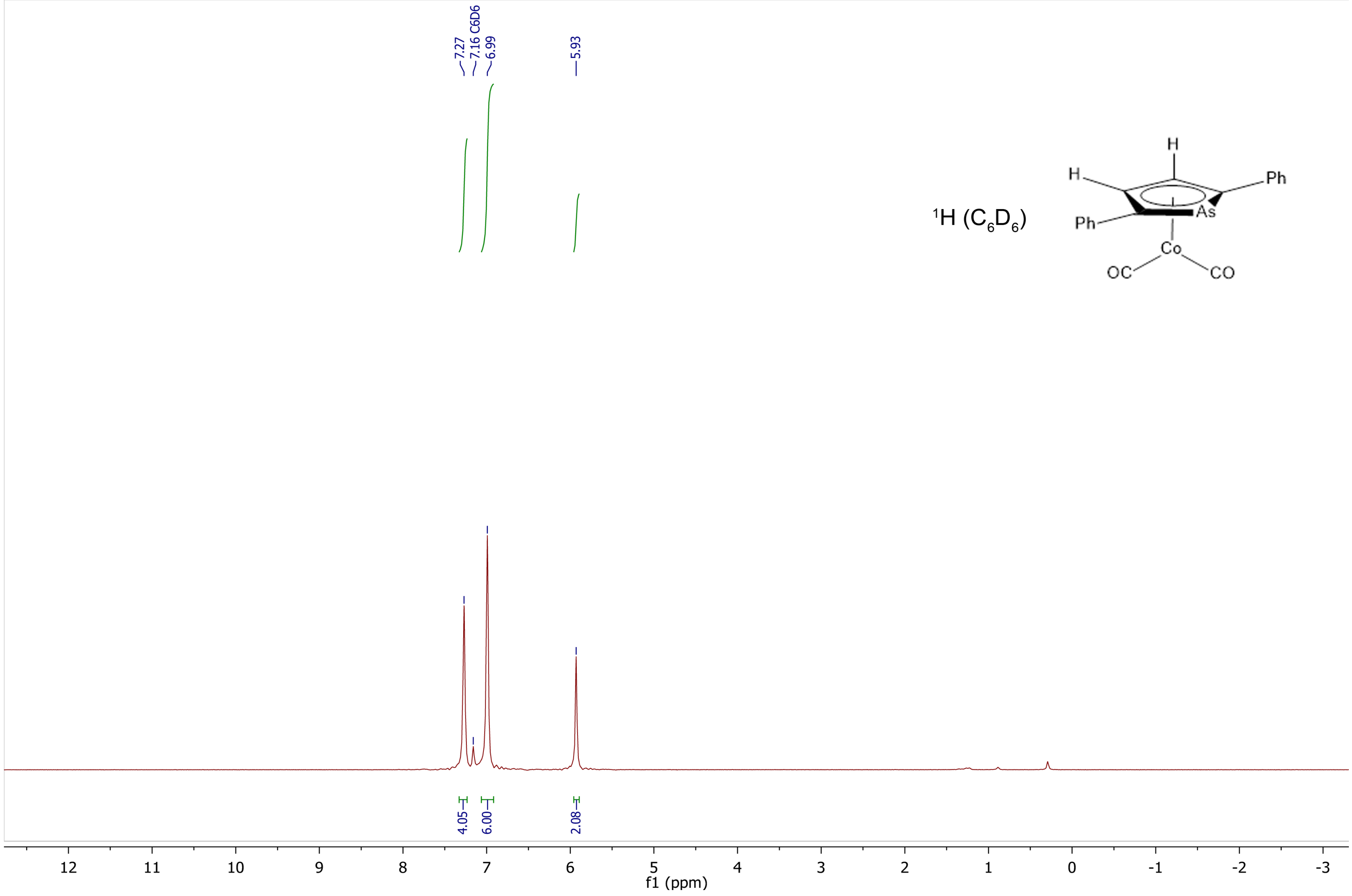
7.27
7.16 C6D6
6.99
5.93
1H (C6D6)
H
H
Ph
Ph
As
Co
OC
CO
4.05
6.00
2.08
12
11
10
9
8
7
6
5
4
3
2
1
0
-1
-2
-3
f1 (ppm)

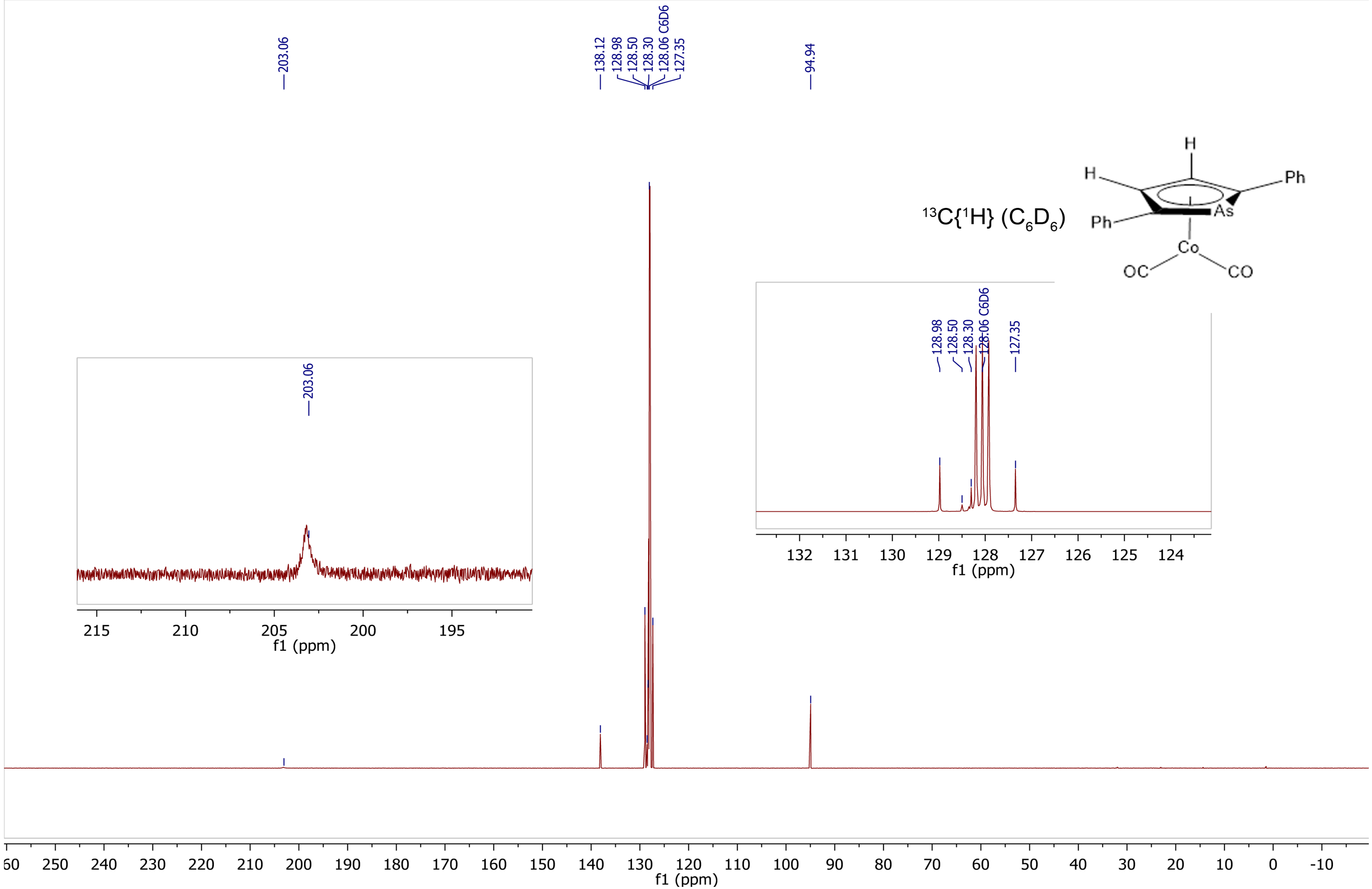
203.06
138.12
128.98
128.50
128.30
128.06 C6D6
127.35
94.94
13C{1H} (C6D6)
H
H
Ph
Ph
As
Co
OC
CO
f1 (ppm)

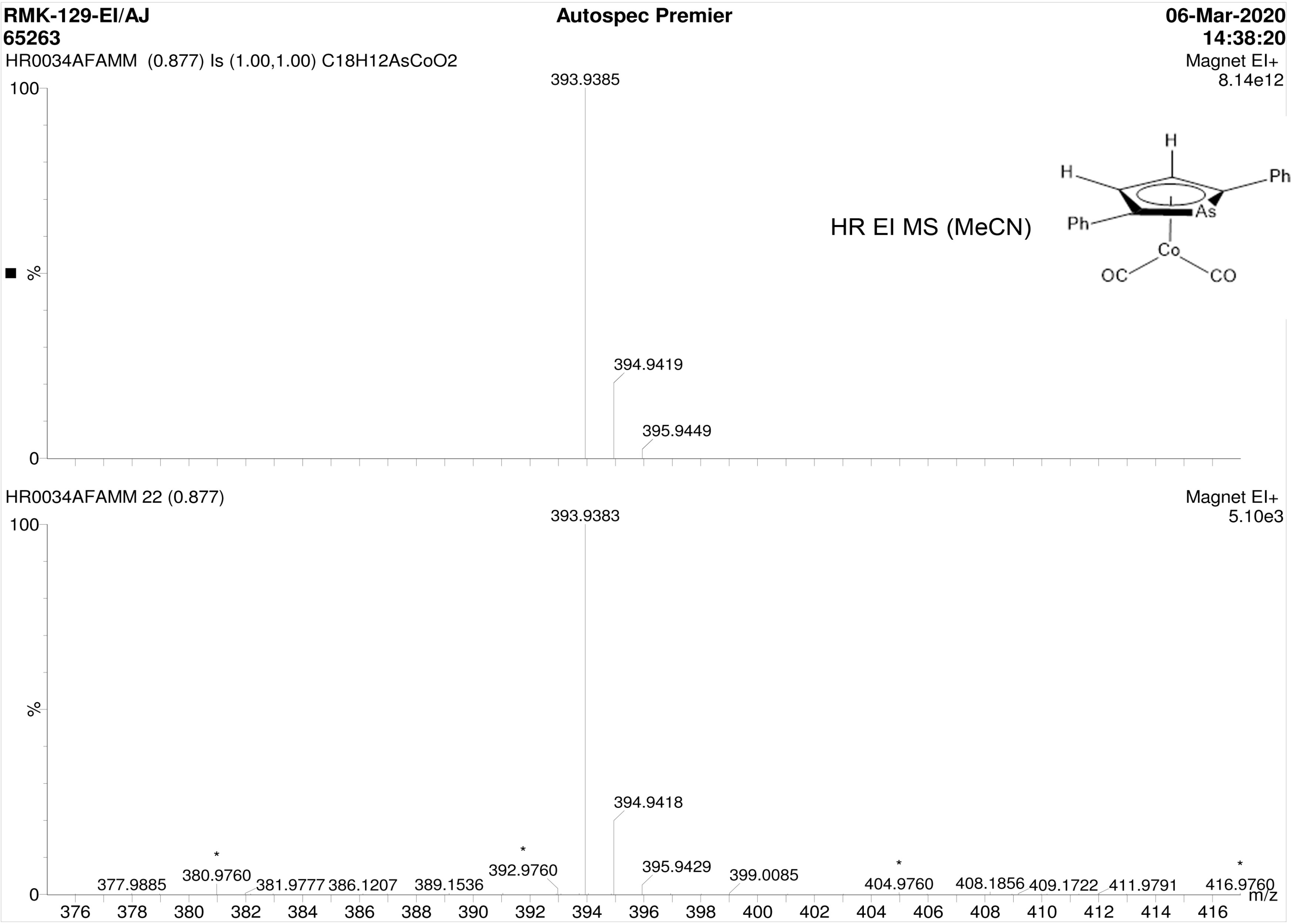

RMK-129-EI/AJ
65263
Autospec Premier
06-Mar-2020
14:38:20
HR0034AFAMM (0.877) Is (1.00,1.00) C18H12AsCoO2
Magnet EI+
8.14e12
393.9385
394.9419
395.9449
HR EI MS (MeCN)
HR0034AFAMM 22 (0.877)
Magnet EI+
5.10e3
393.9383
394.9418
377.9885
380.9760
381.9777
386.1207
389.1536
392.9760
395.9429
399.0085
404.9760
408.1856
409.1722
411.9791
416.9760
m/z
H
H
Ph
Ph
As
Co
OC
CO

6.

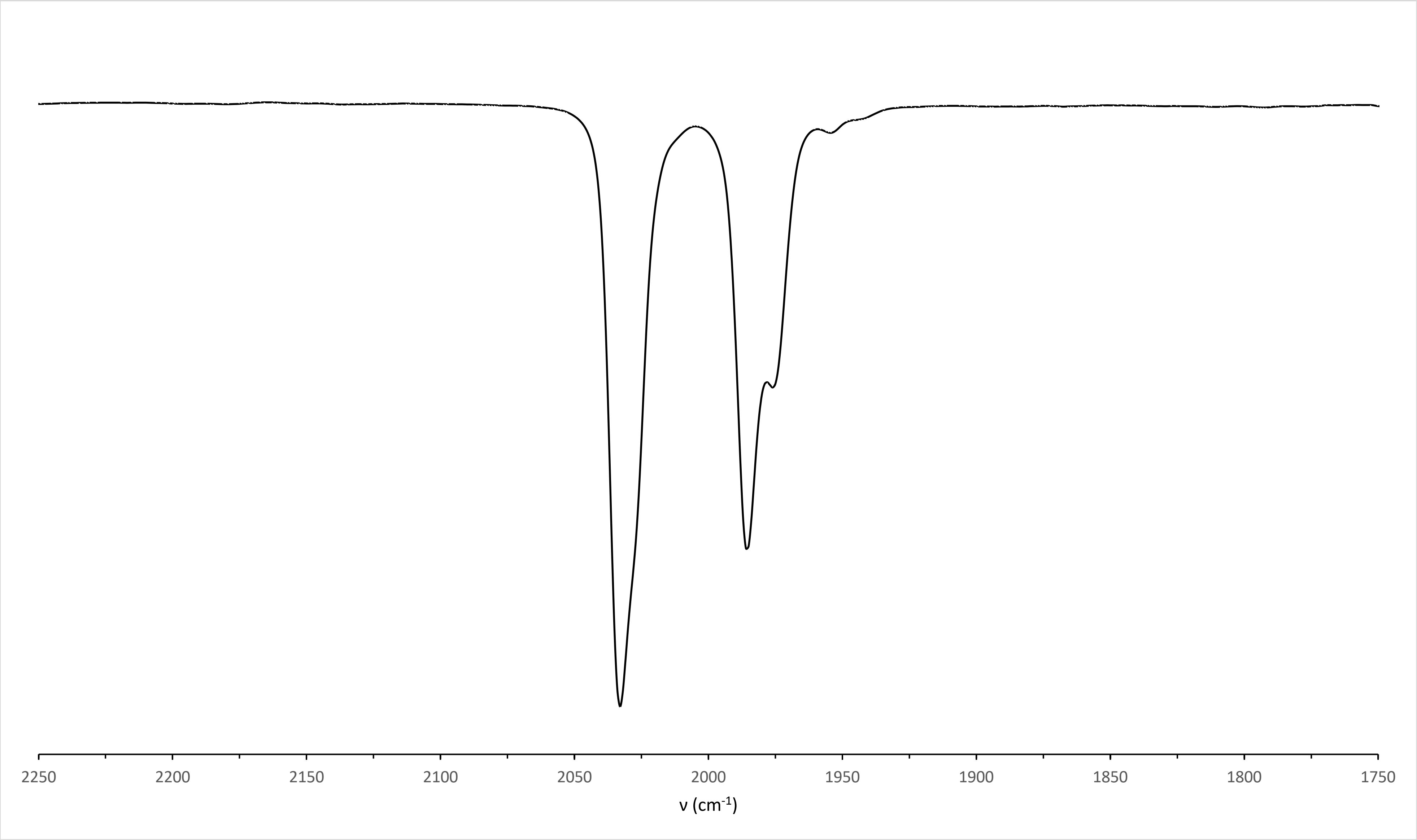
2250
2200
2150
2100
2050
2000
1950
1900
1850
1800
1750
ν (cm-1)

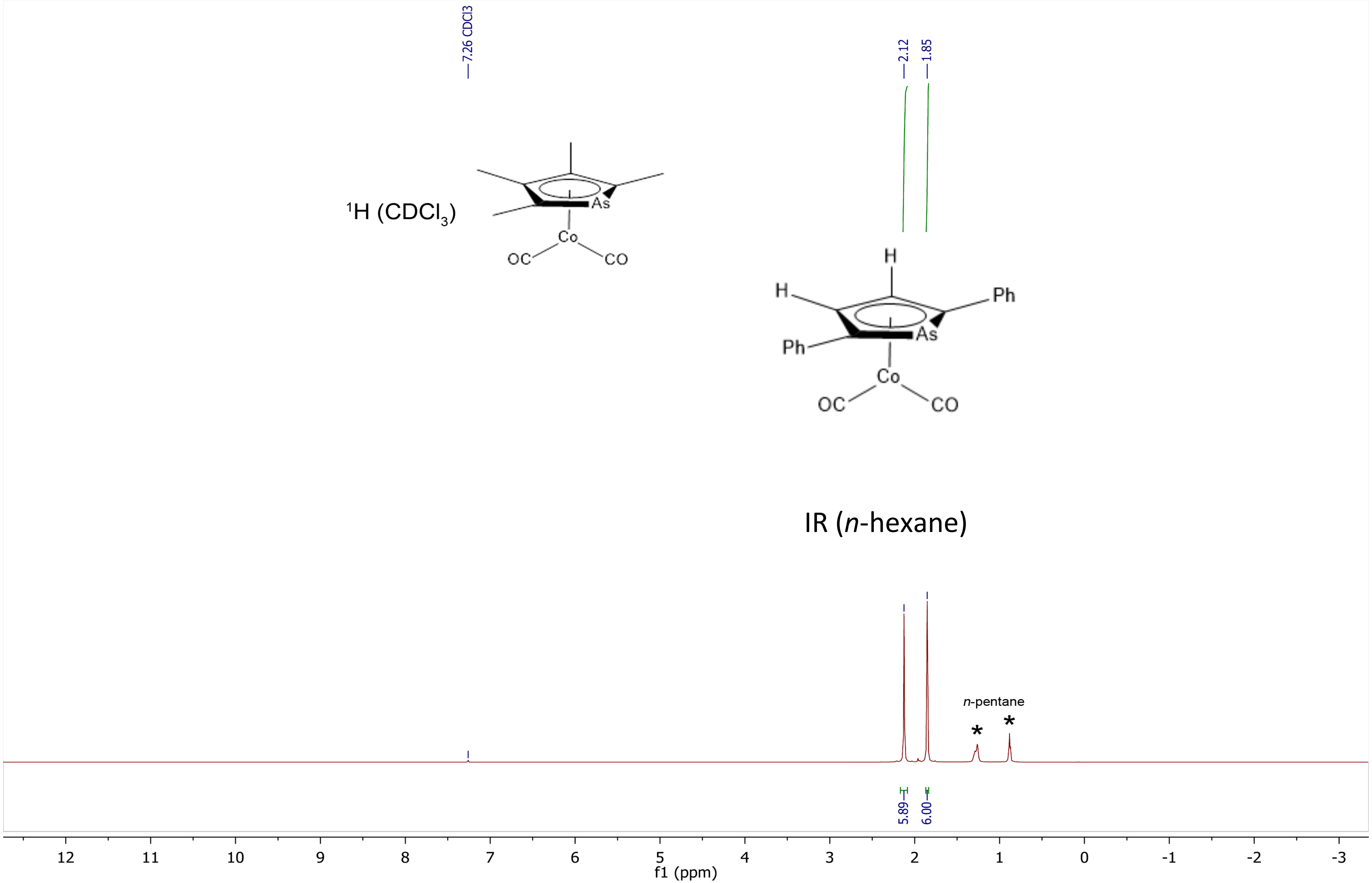
7.26 CDCl3
2.12
1.85
¹H (CDCl₃)
As
Co
OC
CO
H
H
Ph
Ph
As
Co
OC
CO
IR (n-hexane)
n-pentane
*
*
5.89
6.00
12
11
10
9
8
7
6
5
4
3
2
1
0
-1
-2
-3
f1 (ppm)

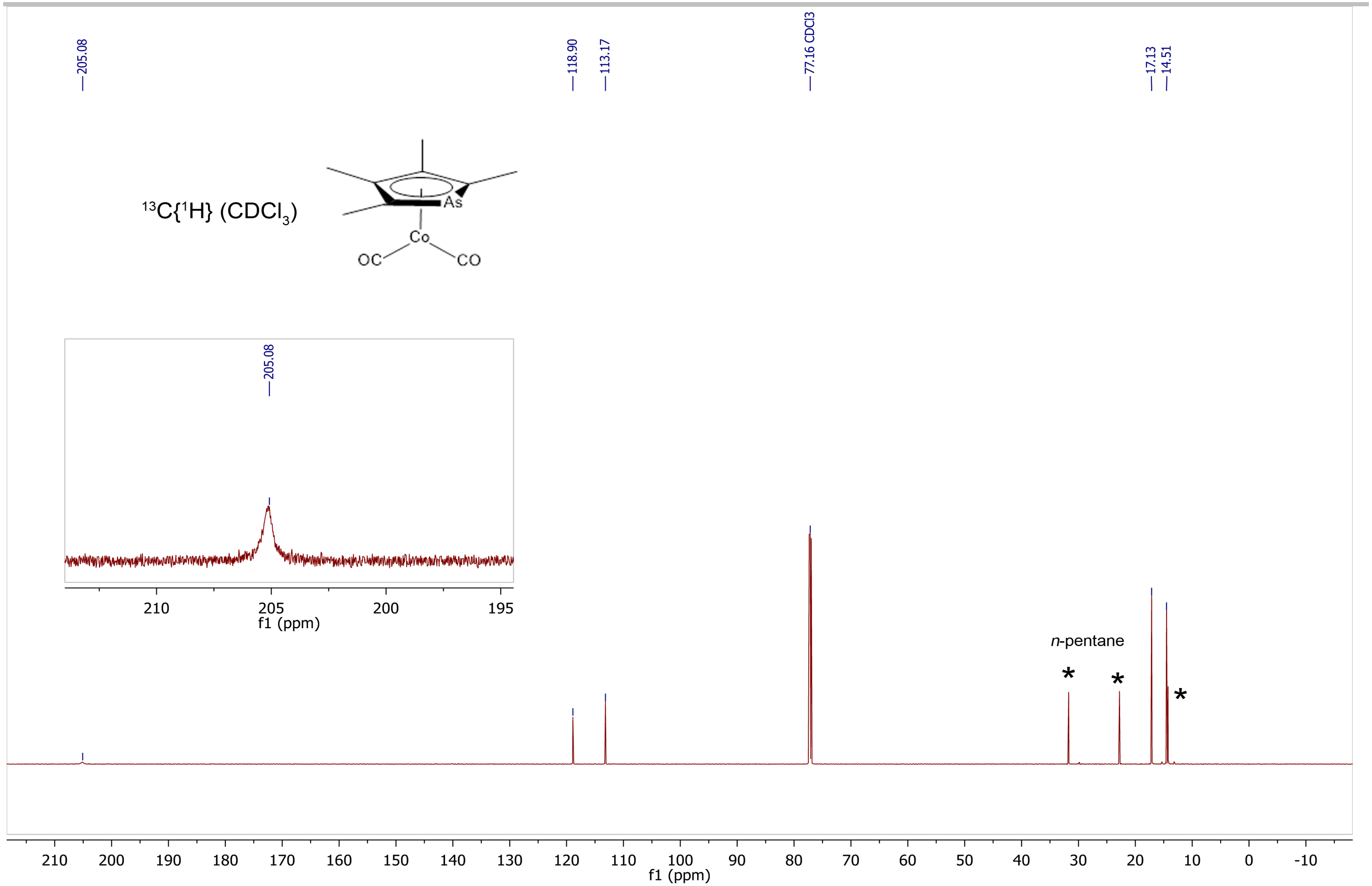
205.08
118.90
113.17
77.16 CDCl3
17.13
14.51
13C{1H} (CDCl3)
As
Co
OC
CO
205.08
210
205
200
195
f1 (ppm)
n-pentane
*
*
*
210
200
190
180
170
160
150
140
130
120
110
100
90
80
70
60
50
40
30
20
10
0
-10
f1 (ppm)

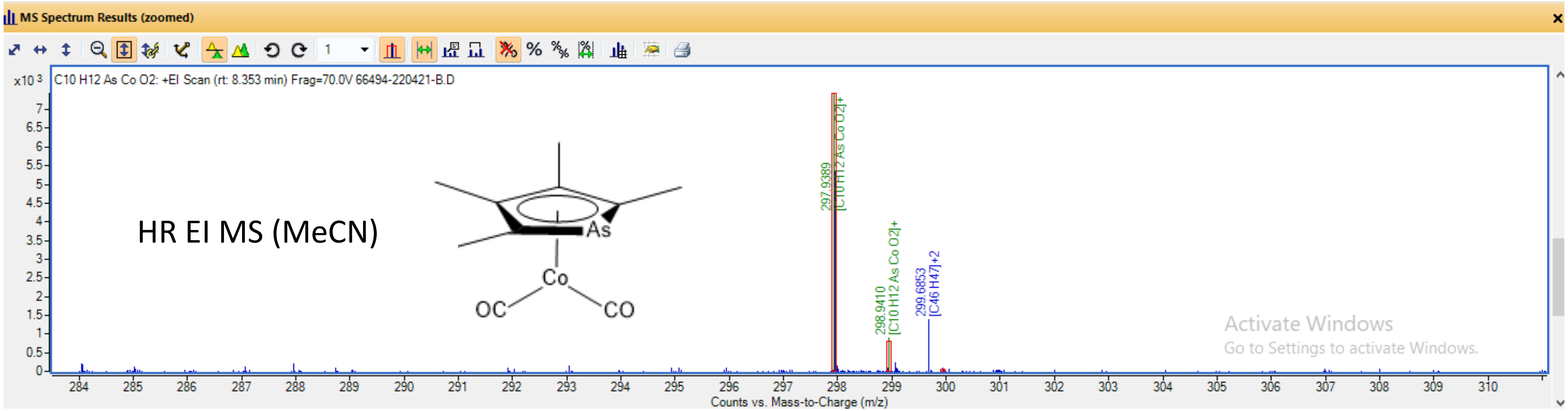
MS Spectrum Results (zoomed)
x10 3
C10 H12 As Co O2: +EI Scan (rt: 8.353 min) Frag=70.0V 66494-220421-B.D
HR EI MS (MeCN)
As
Co
OC
CO
297.9389
[C10 H12 As Co O2]+
298.9410
[C10 H12 As Co O2]+
299.6853
[C46 H47]+2
Activate Windows
Go to Settings to activate Windows.
Counts vs. Mass-to-Charge (m/z)

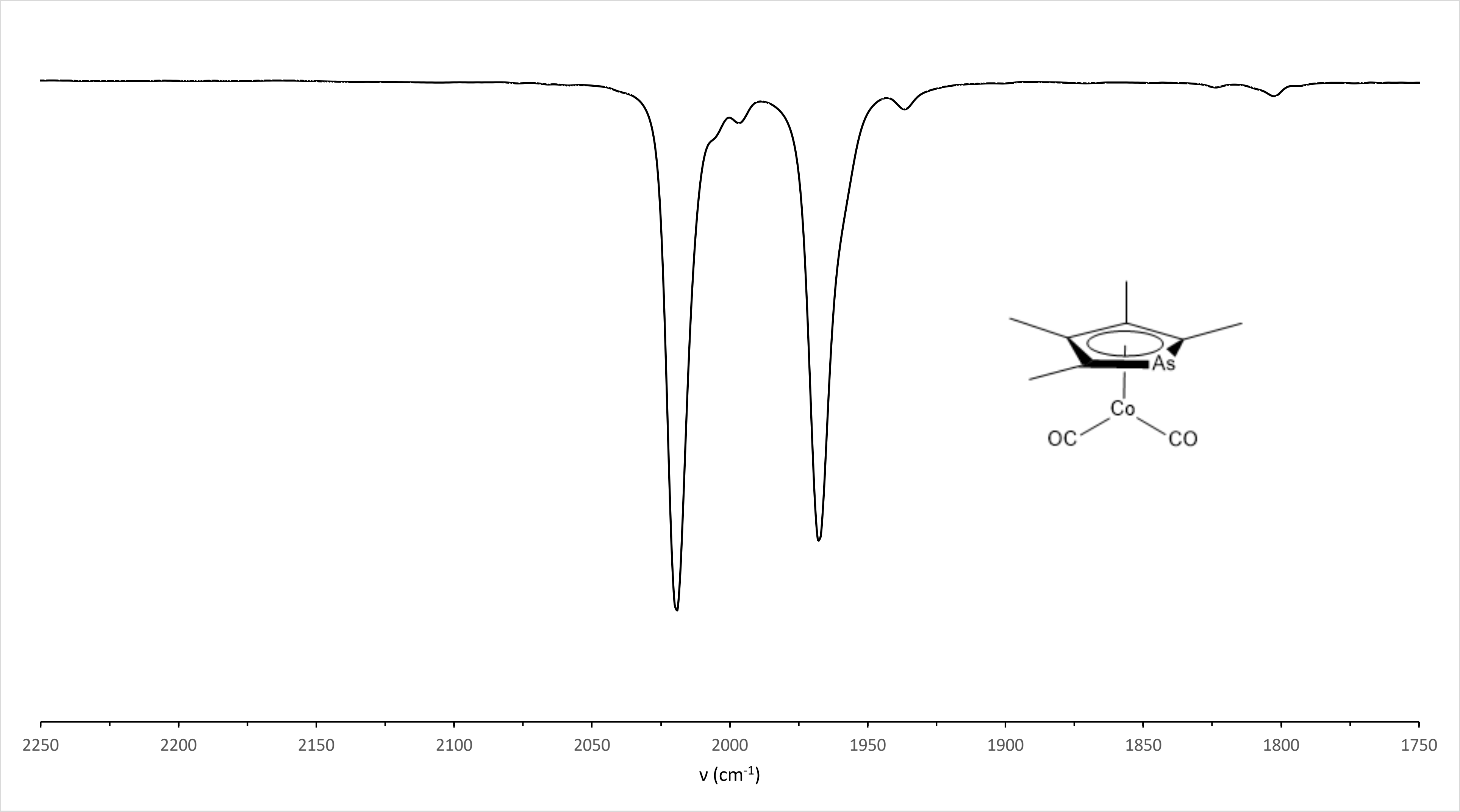
As
Co
OC
CO
2250
2200
2150
2100
2050
2000
1950
1900
1850
1800
1750
ν (cm-1)

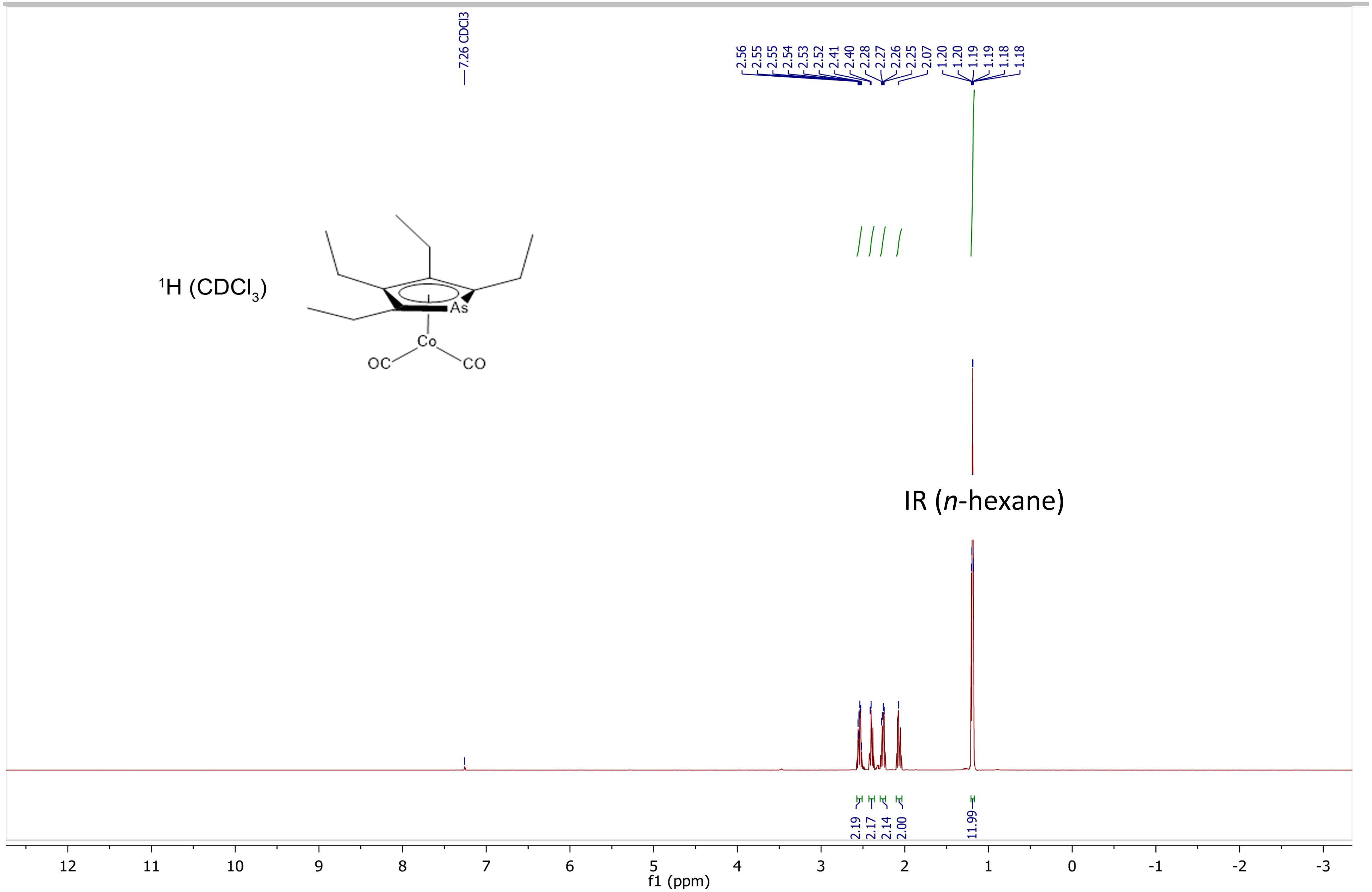

7.26 CDCl3
2.56
2.55
2.55
2.54
2.53
2.52
2.41
2.40
2.28
2.27
2.26
2.25
2.07
1.20
1.20
1.19
1.19
1.18
1.18
1H (CDCl3)
As
Co
OC
CO
IR (n-hexane)
2.19
2.17
2.14
2.00
11.99
12
11
10
9
8
7
6
5
4
3
2
1
0
-1
-2
-3
f1 (ppm)

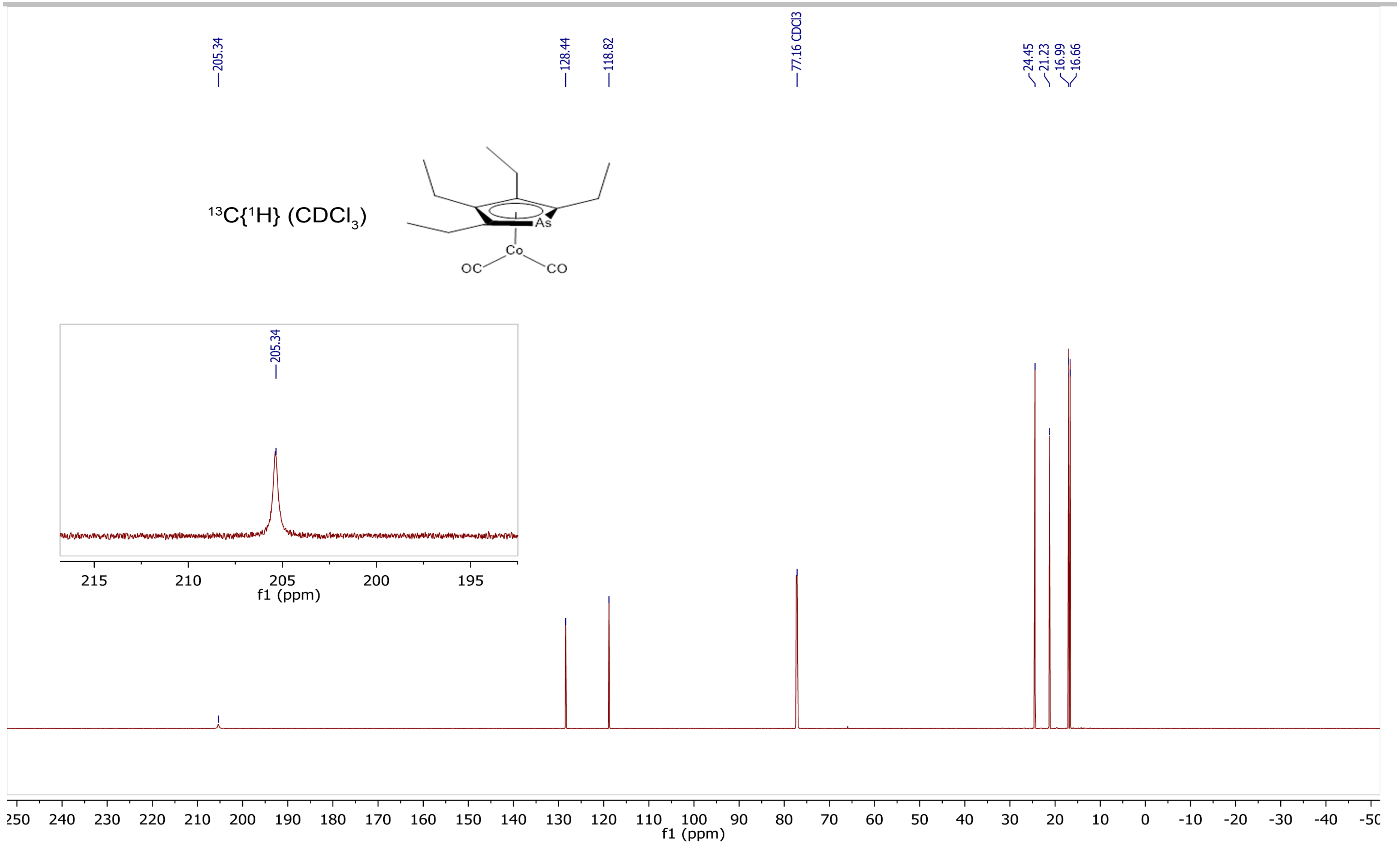

205.34
128.44
118.82
77.16 CDCl3
24.45
21.23
16.99
16.66
$^{13}C\{^1H\}$ ($CDCl_3$)
As
Co
OC
CO
205.34
215
210
205
200
195
f1 (ppm)
250
240
230
220
210
200
190
180
170
160
150
140
130
120
110
100
90
80
70
60
50
40
30
20
10
0
-10
-20
-30
-40
-50
f1 (ppm)

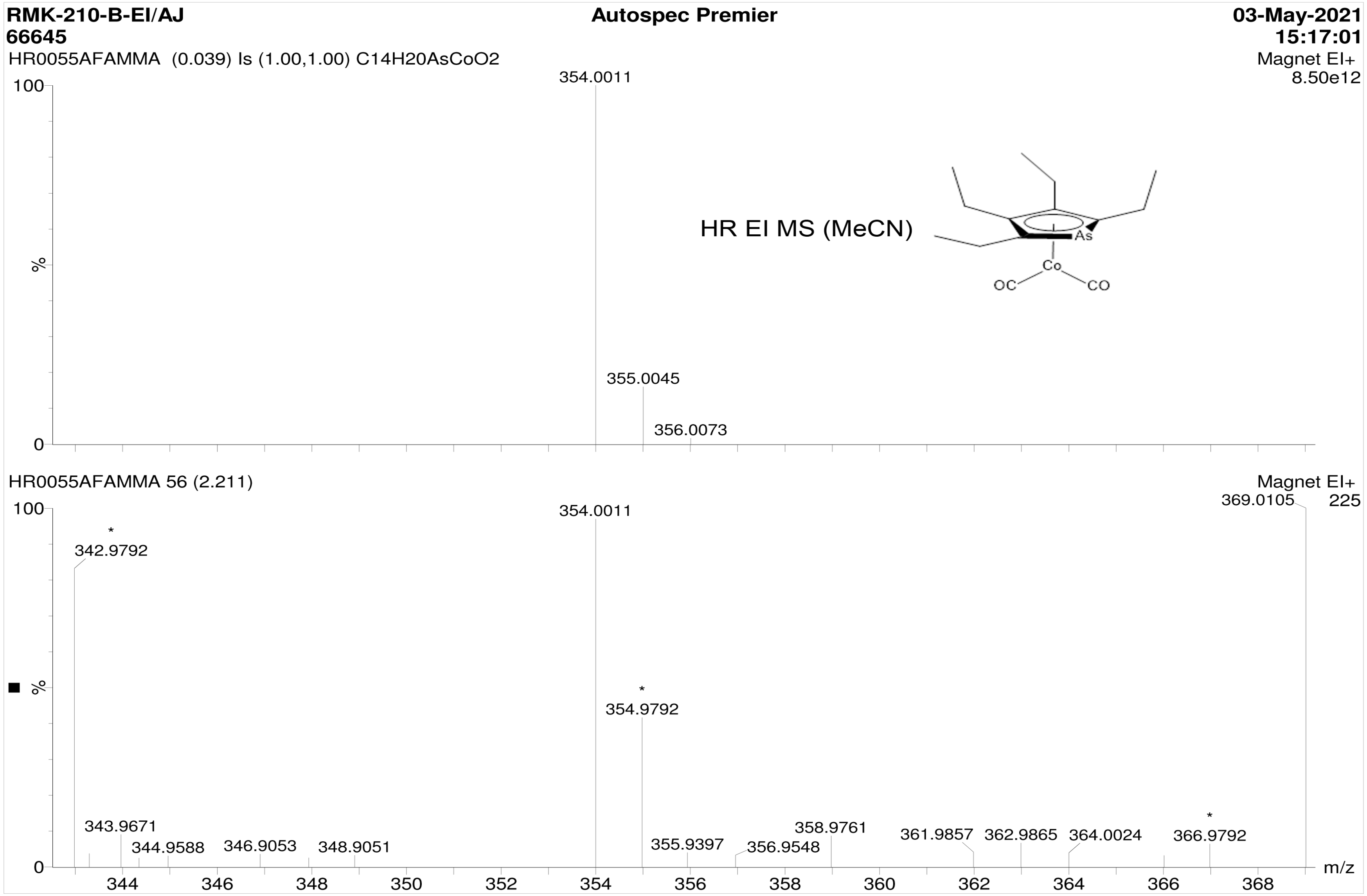
RMK-210-B-EI/AJ
66645
Autospec Premier
03-May-2021
15:17:01
HR0055AFAMMA (0.039) Is (1.00,1.00) C14H20AsCoO2
Magnet EI+
8.50e12
100
%
0
354.0011
355.0045
356.0073
HR EI MS (MeCN)
As
Co
OC
CO
HR0055AFAMMA 56 (2.211)
Magnet EI+
369.0105
225
354.0011
*
342.9792
*
354.9792
343.9671
344.9588
346.9053
348.9051
355.9397
356.9548
358.9761
361.9857
362.9865
364.0024
*
366.9792
344
346
348
350
352
354
356
358
360
362
364
366
368
m/z

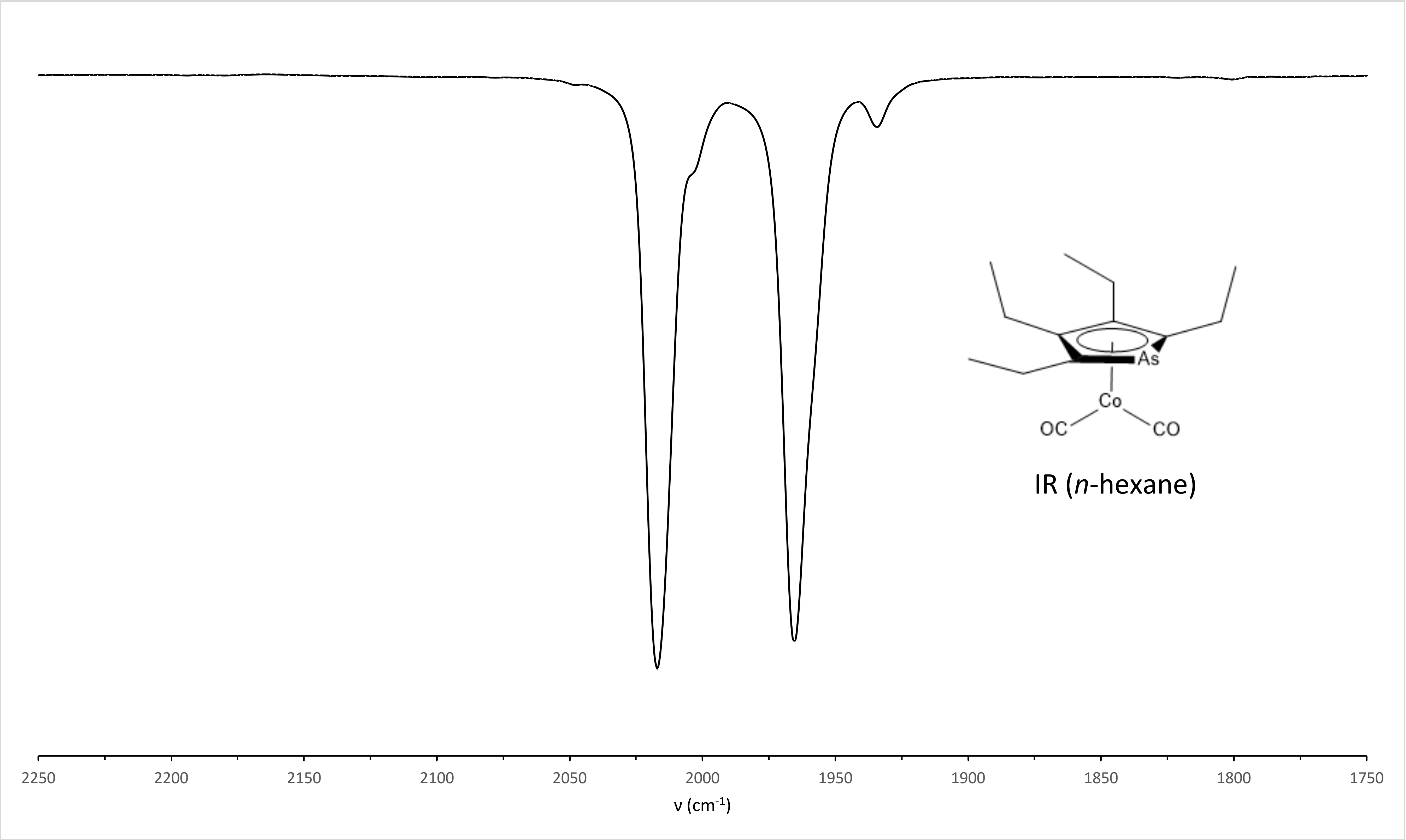
As
Co
OC
CO
IR (n-hexane)
2250
2200
2150
2100
2050
2000
1950
1900
1850
1800
1750
ν (cm-1)

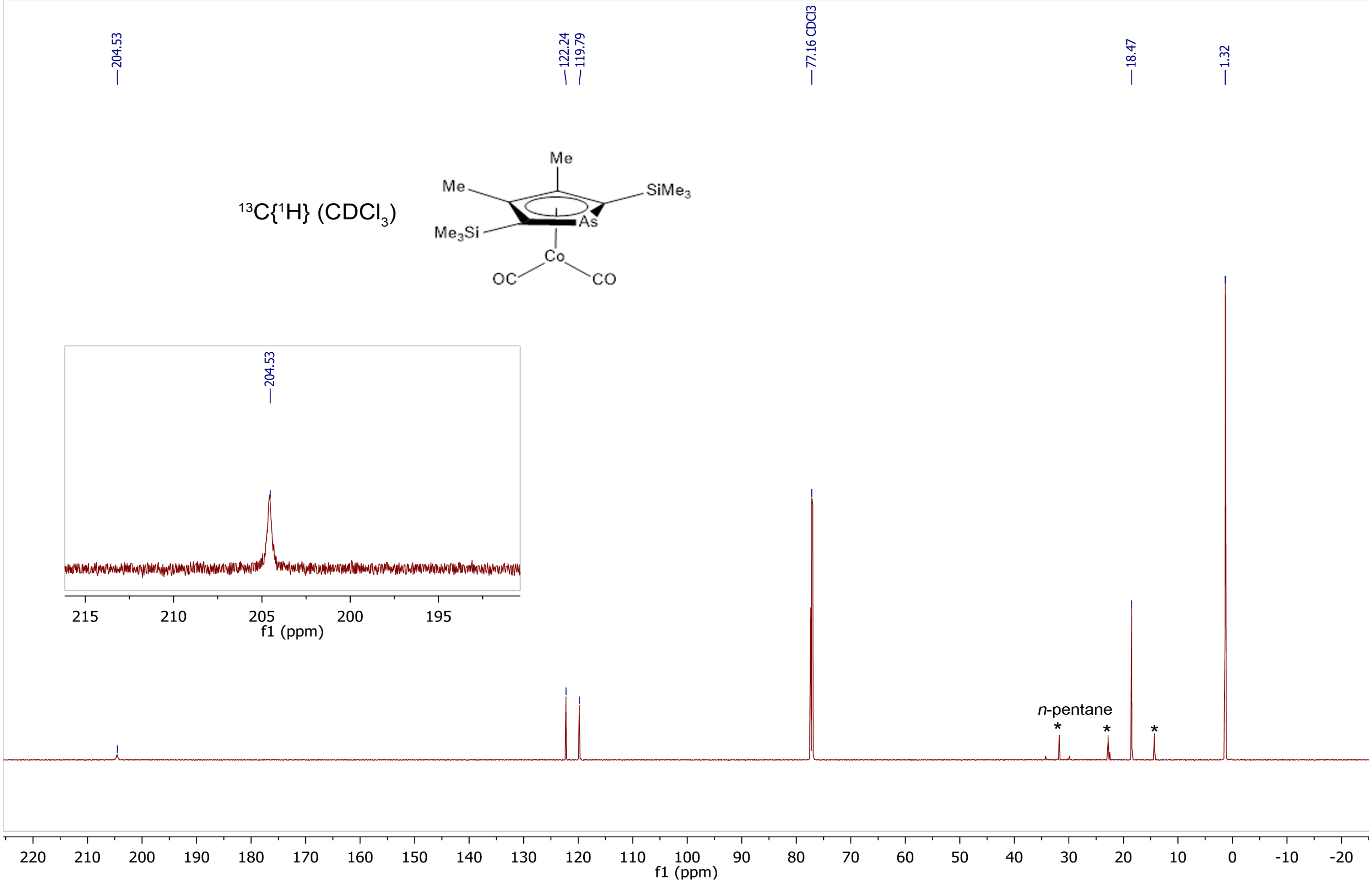

204.53
122.24
119.79
77.16 CDCl3
18.47
1.32
$^{13}C\{^1H\}$ ($CDCl_3$)
Me
Me
SiMe3
Me3Si
As
Co
OC
CO
204.53
215
210
205
200
195
f1 (ppm)
n-pentane
*
*
*
220
210
200
190
180
170
160
150
140
130
120
110
100
90
80
70
60
50
40
30
20
10
0
-10
-20
f1 (ppm)

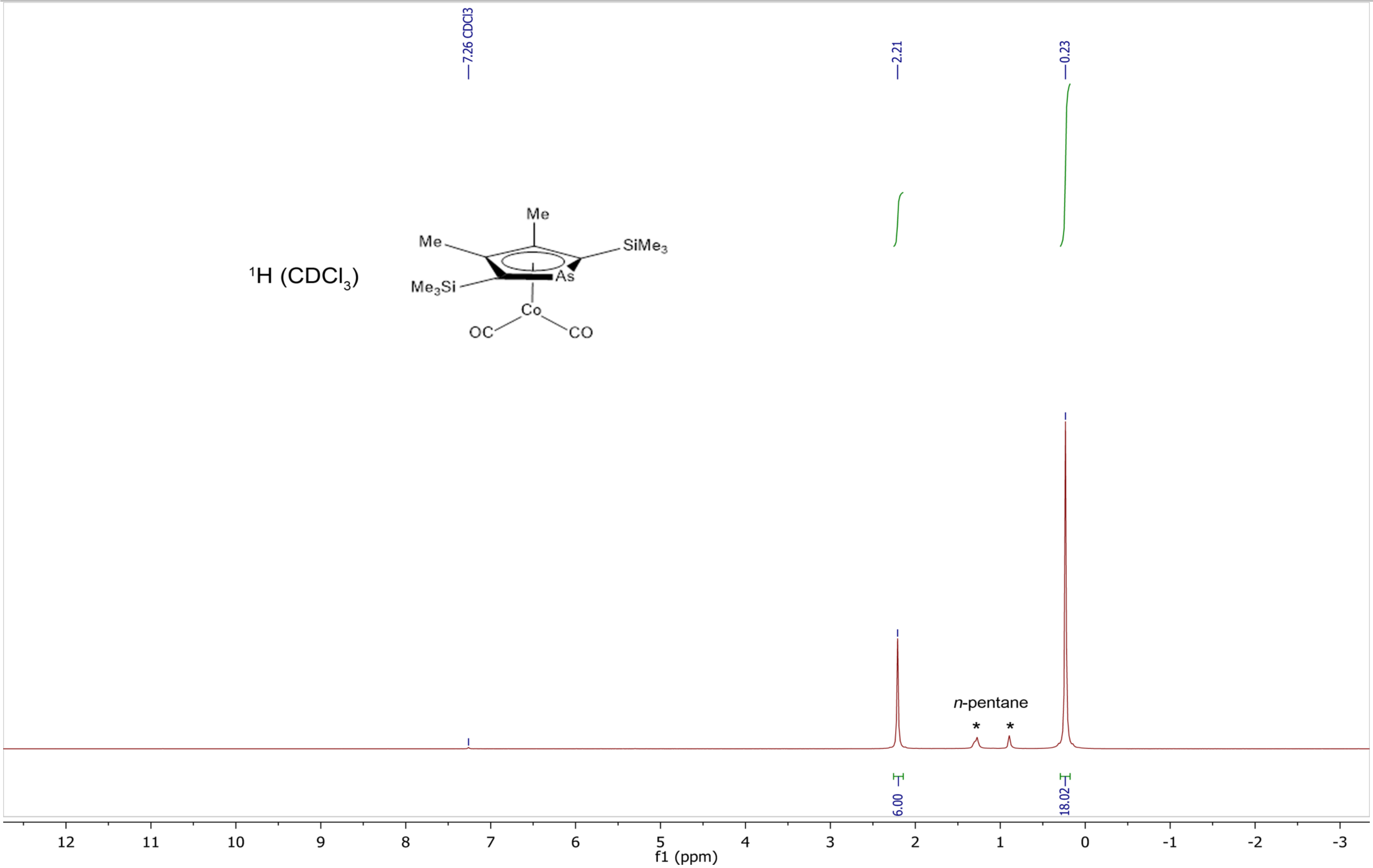
7.26 CDCl3
2.21
0.23
¹H (CDCl3)
Me
Me
SiMe3
Me3Si
As
Co
OC
CO
n-pentane
*
*
6.00
18.02
12
11
10
9
8
7
6
5
4
3
2
1
0
-1
-2
-3
f1 (ppm)

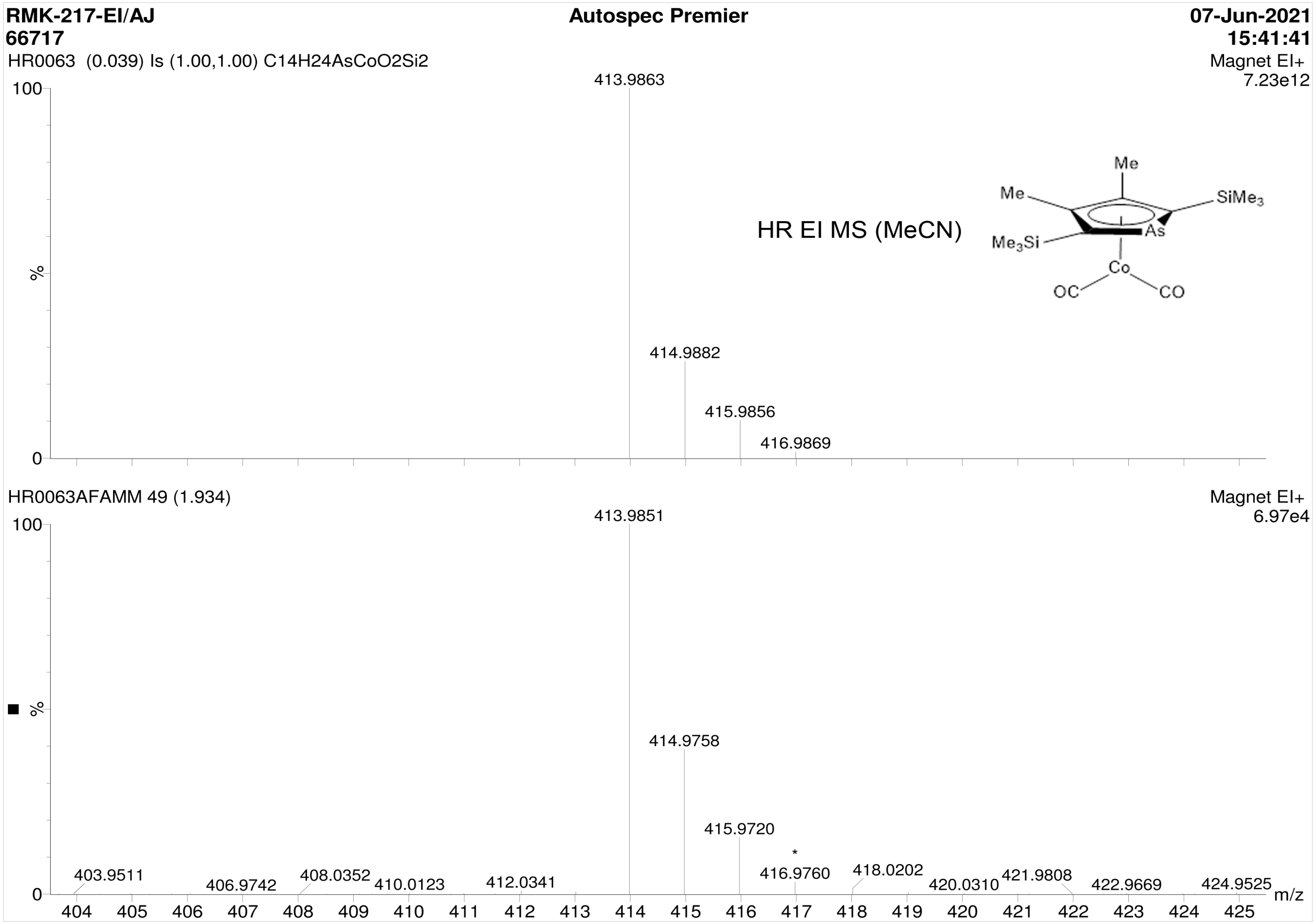
RMK-217-EI/AJ
66717
Autospec Premier
07-Jun-2021
15:41:41
HR0063 (0.039) Is (1.00,1.00) C14H24AsCoO2Si2
Magnet EI+
7.23e12
413.9863
414.9882
415.9856
416.9869
HR EI MS (MeCN)
Me
Me
SiMe3
Me3Si
As
Co
OC
CO
HR0063AFAMM 49 (1.934)
Magnet EI+
6.97e4
413.9851
414.9758
415.9720
403.9511
406.9742
408.0352
410.0123
412.0341
416.9760
418.0202
420.0310
421.9808
422.9669
424.9525
m/z
%
100
0

7.

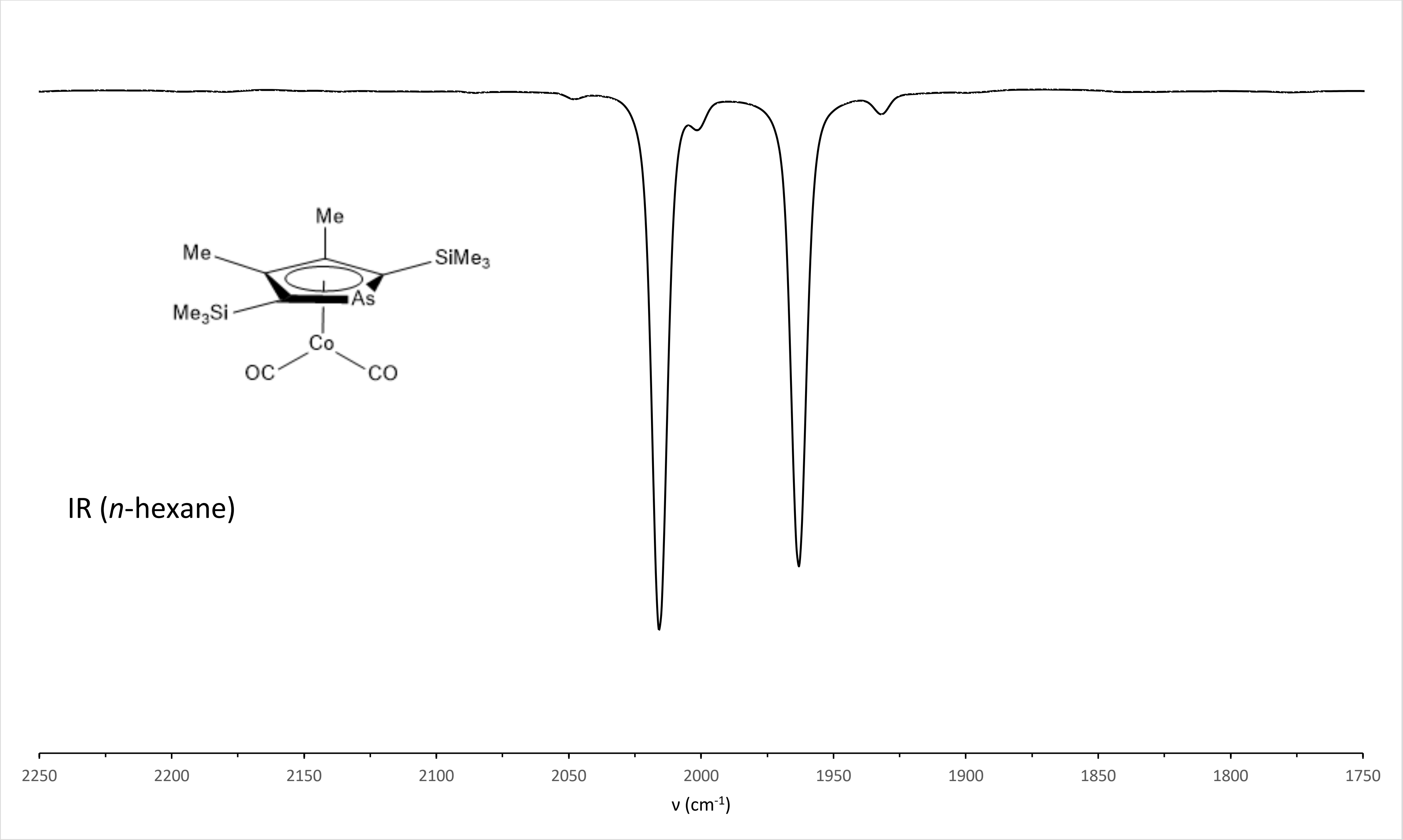

Me
Me
SiMe3
As
Me3Si
Co
OC
CO
IR (n-hexane)
2250
2200
2150
2100
2050
2000
1950
1900
1850
1800
1750
ν (cm-1)

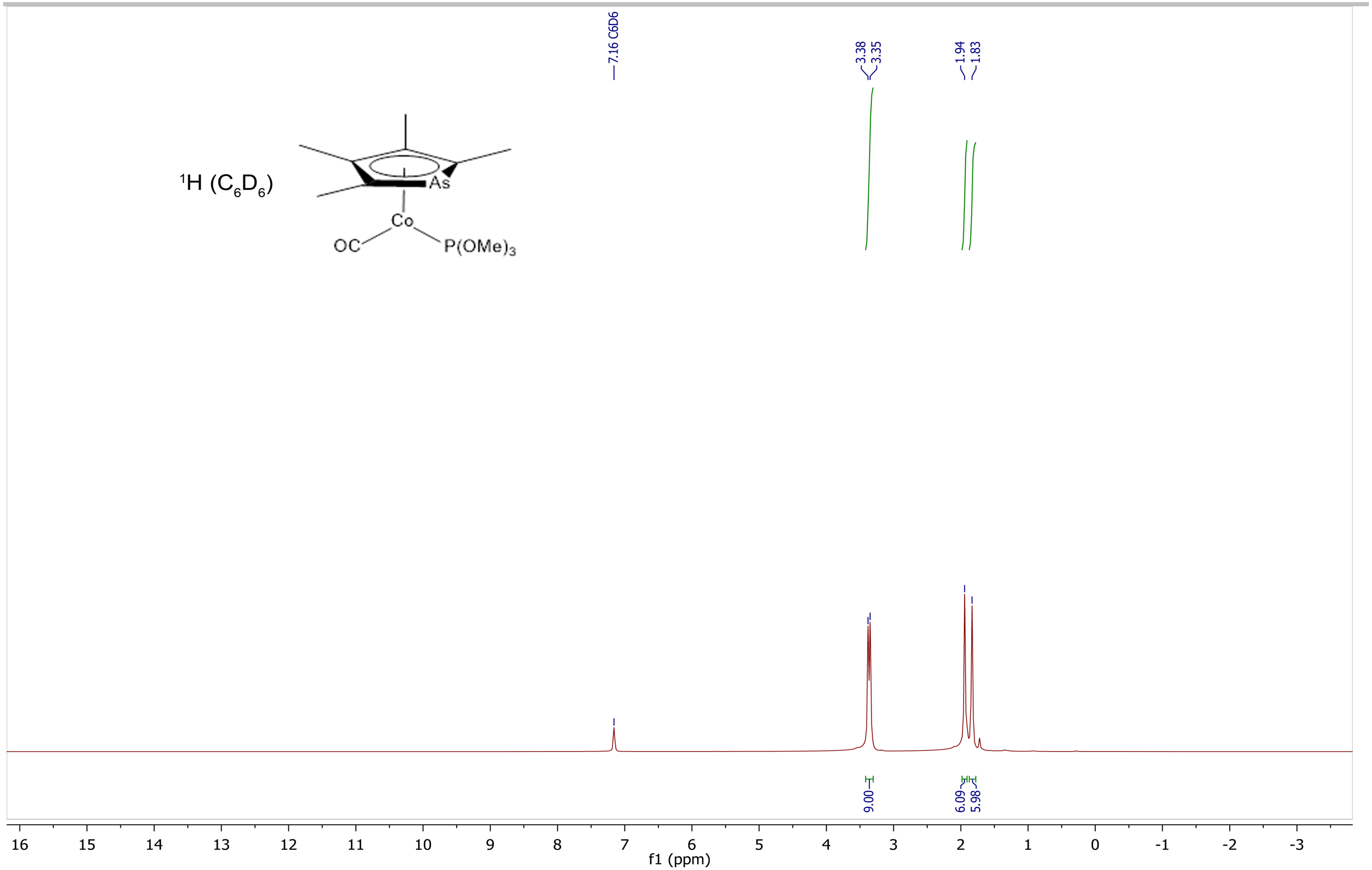

[1]H (C6D6)
As
Co
OC
P(OMe)3
7.16 C6D6
3.38
3.35
1.94
1.83
9.00
6.09
5.98
f1 (ppm)

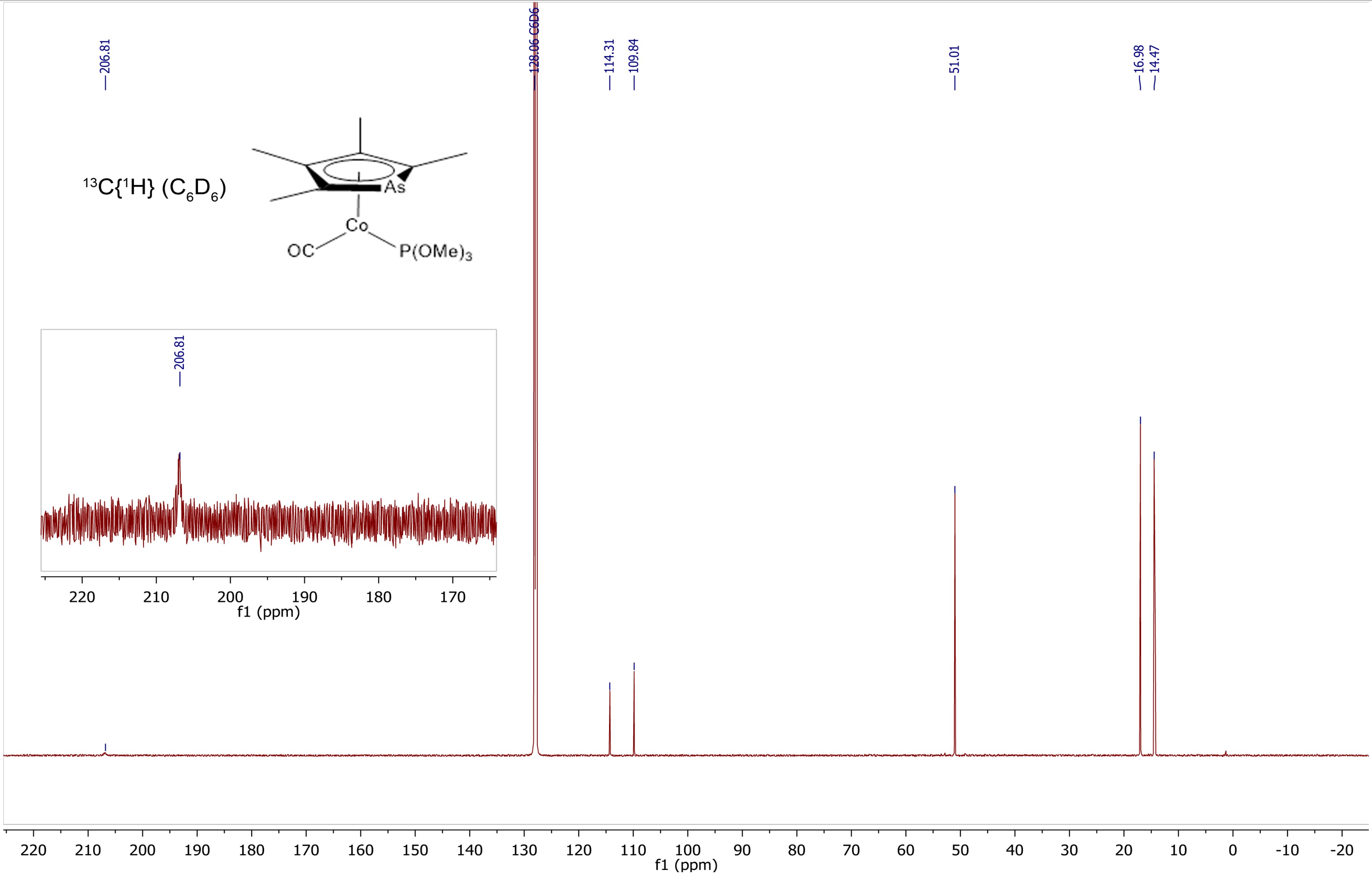
206.81
128.06 C6D6
114.31
109.84
51.01
16.98
14.47
$^{13}C\{^1H\}$ (C_6D_6)
As
Co
OC
P(OMe)3
206.81
220 210 200 190 180 170
f1 (ppm)
220 210 200 190 180 170 160 150 140 130 120 110 100 90 80 70 60 50 40 30 20 10 0 -10 -20
f1 (ppm)

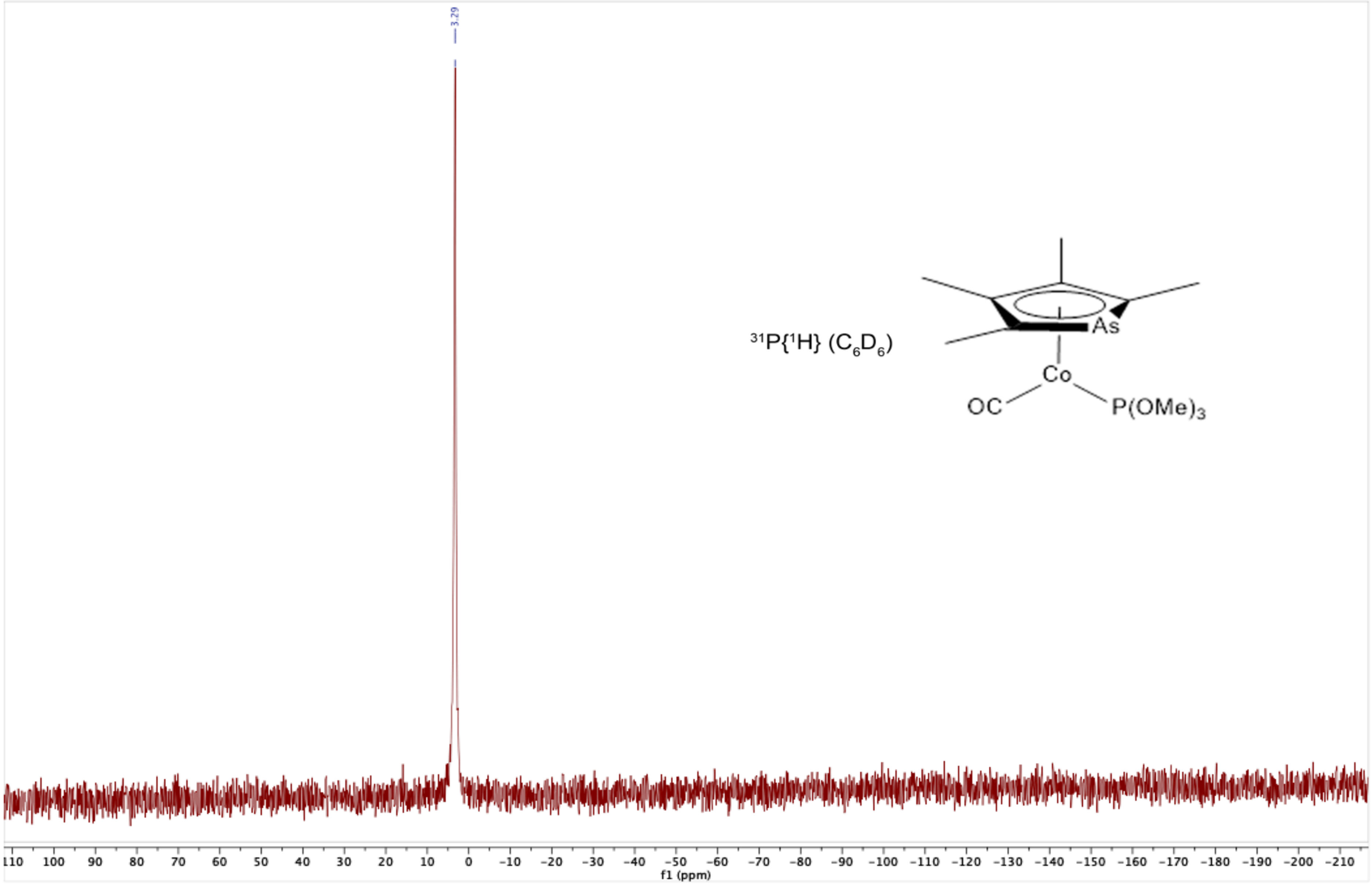
3.29
$^{31}P\{^{1}H\}$ (C_6D_6)
As
Co
OC
P(OMe)3
110 100 90 80 70 60 50 40 30 20 10 0 -10 -20 -30 -40 -50 -60 -70 -80 -90 -100 -110 -120 -130 -140 -150 -160 -170 -180 -190 -200 -210
f1 (ppm)

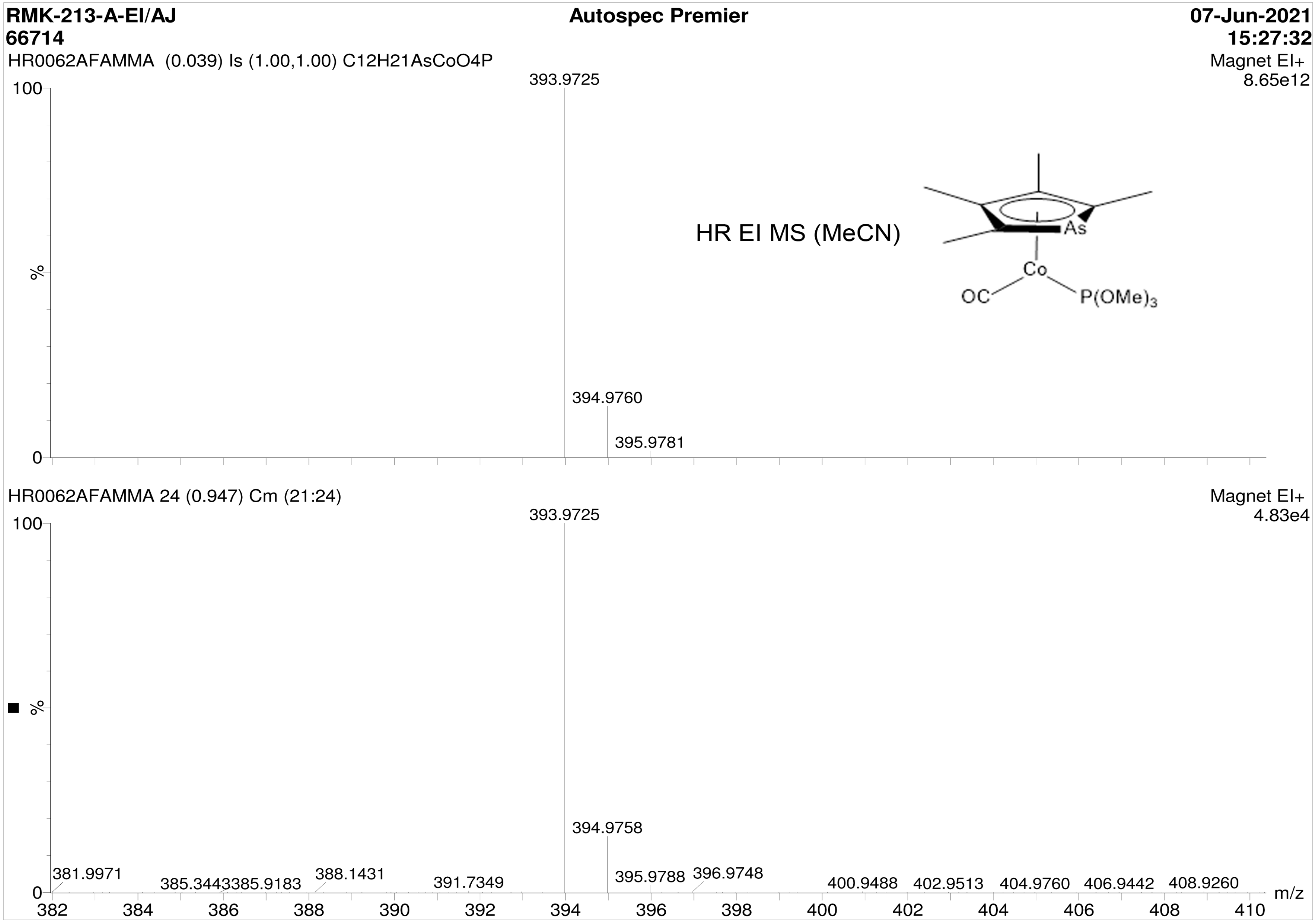
RMK-213-A-EI/AJ
66714
Autospec Premier
07-Jun-2021
15:27:32
HR0062AFAMMA (0.039) Is (1.00,1.00) C12H21AsCoO4P
Magnet EI+
8.65e12
393.9725
394.9760
395.9781
HR EI MS (MeCN)
As
Co
OC
P(OMe)3
HR0062AFAMMA 24 (0.947) Cm (21:24)
Magnet EI+
4.83e4
393.9725
394.9758
381.9971
385.3443385.9183
388.1431
391.7349
395.9788
396.9748
400.9488
402.9513
404.9760
406.9442
408.9260
%
100
0
m/z
382
384
386
388
390
392
394
396
398
400
402
404
406
408
410

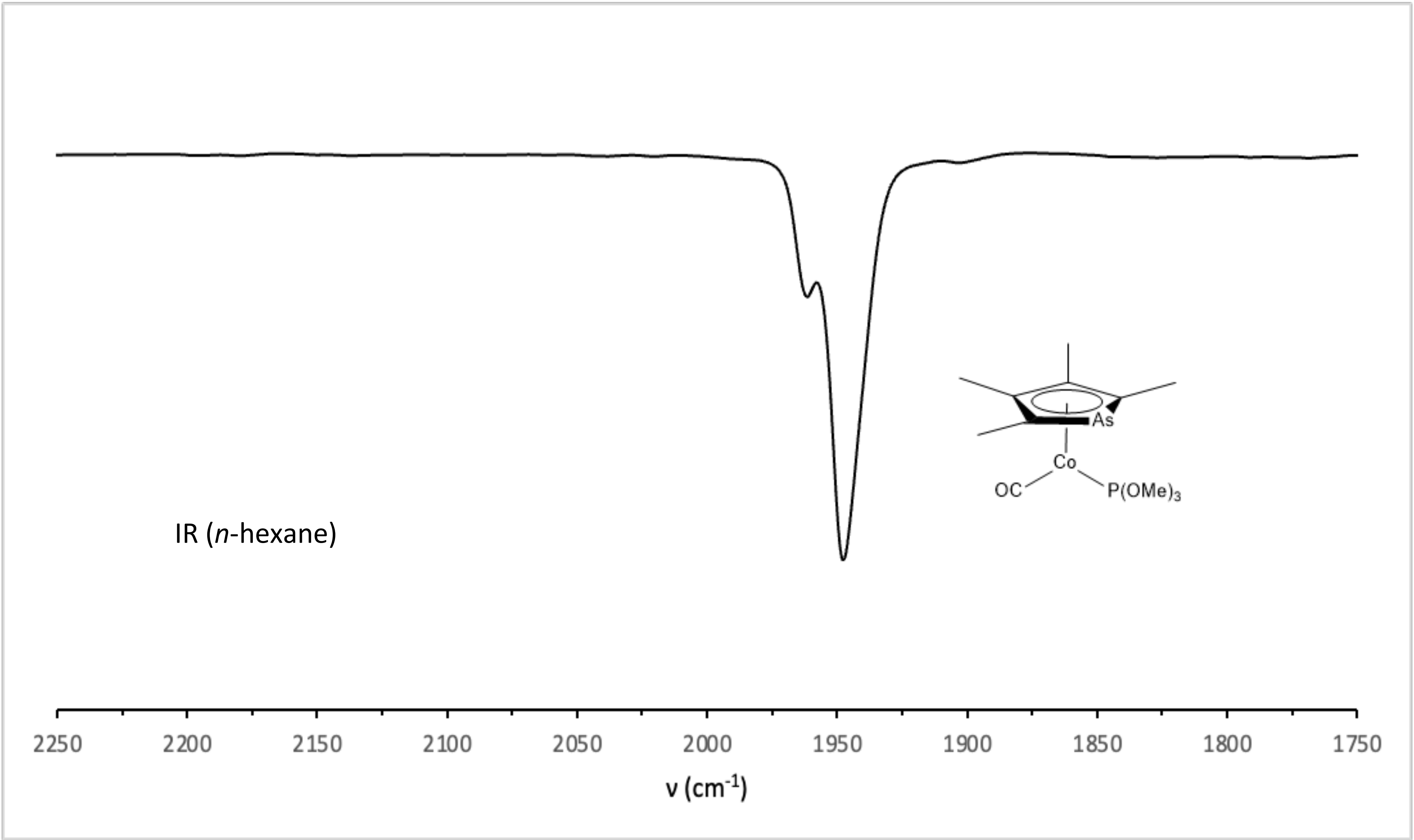
IR (n-hexane)
As
Co
OC
P(OMe)3
2250
2200
2150
2100
2050
2000
1950
1900
1850
1800
1750
ν (cm-1)

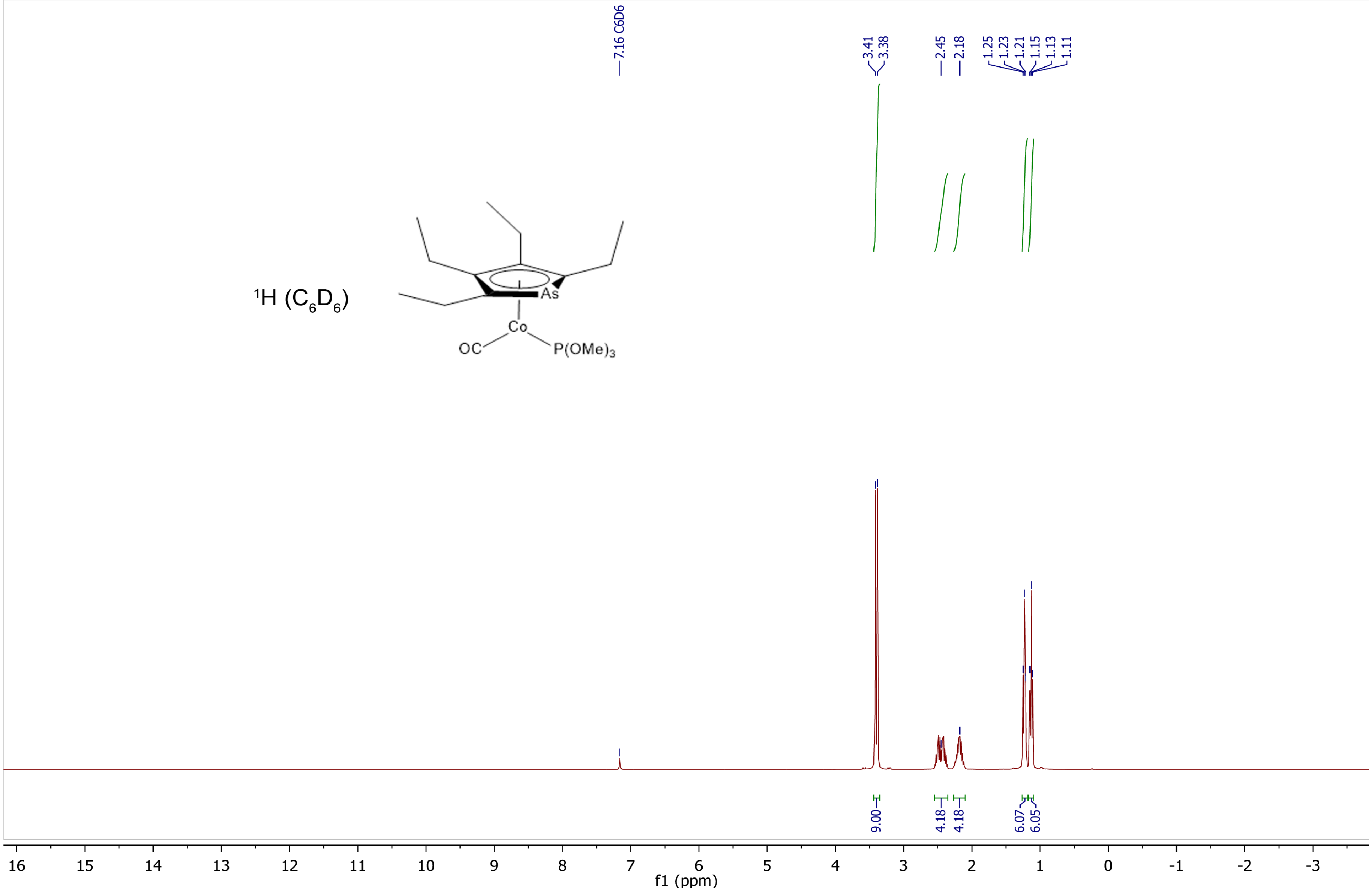

7.16 C6D6
3.41
3.38
2.45
2.18
1.25
1.23
1.21
1.15
1.13
1.11
^{1}H (C_6D_6)
As
Co
OC
P(OMe)3
9.00
4.18
4.18
6.07
6.05
16 15 14 13 12 11 10 9 8 7 6 5 4 3 2 1 0 -1 -2 -3
f1 (ppm)

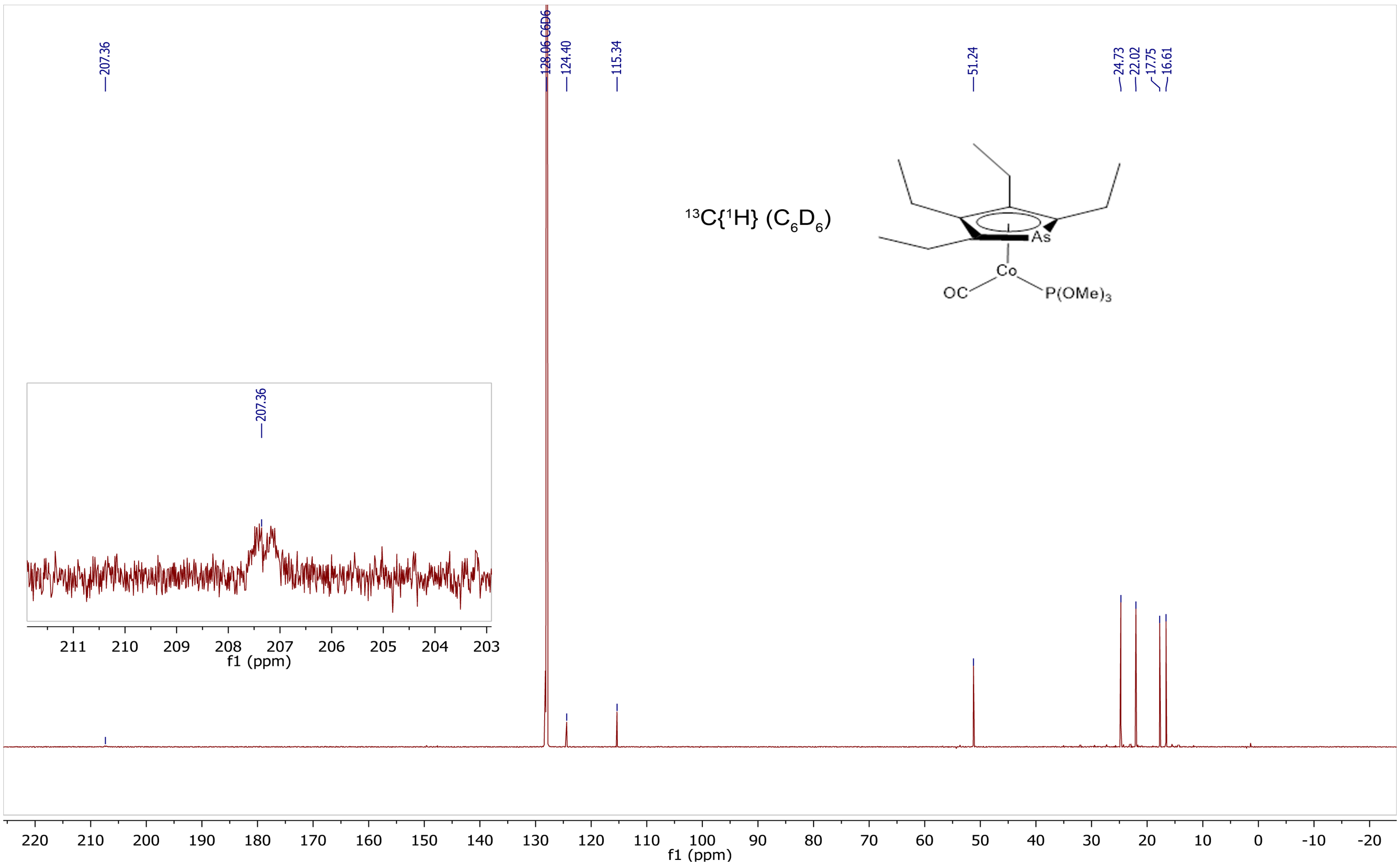
207.36
128.06 C6D6
124.40
115.34
51.24
24.73
22.02
17.75
16.61
$^{13}C\{^{1}H\}$ (C_6D_6)
As
Co
OC
P(OMe)3
207.36
211 210 209 208 207 206 205 204 203
f1 (ppm)
220 210 200 190 180 170 160 150 140 130 120 110 100 90 80 70 60 50 40 30 20 10 0 -10 -20
f1 (ppm)

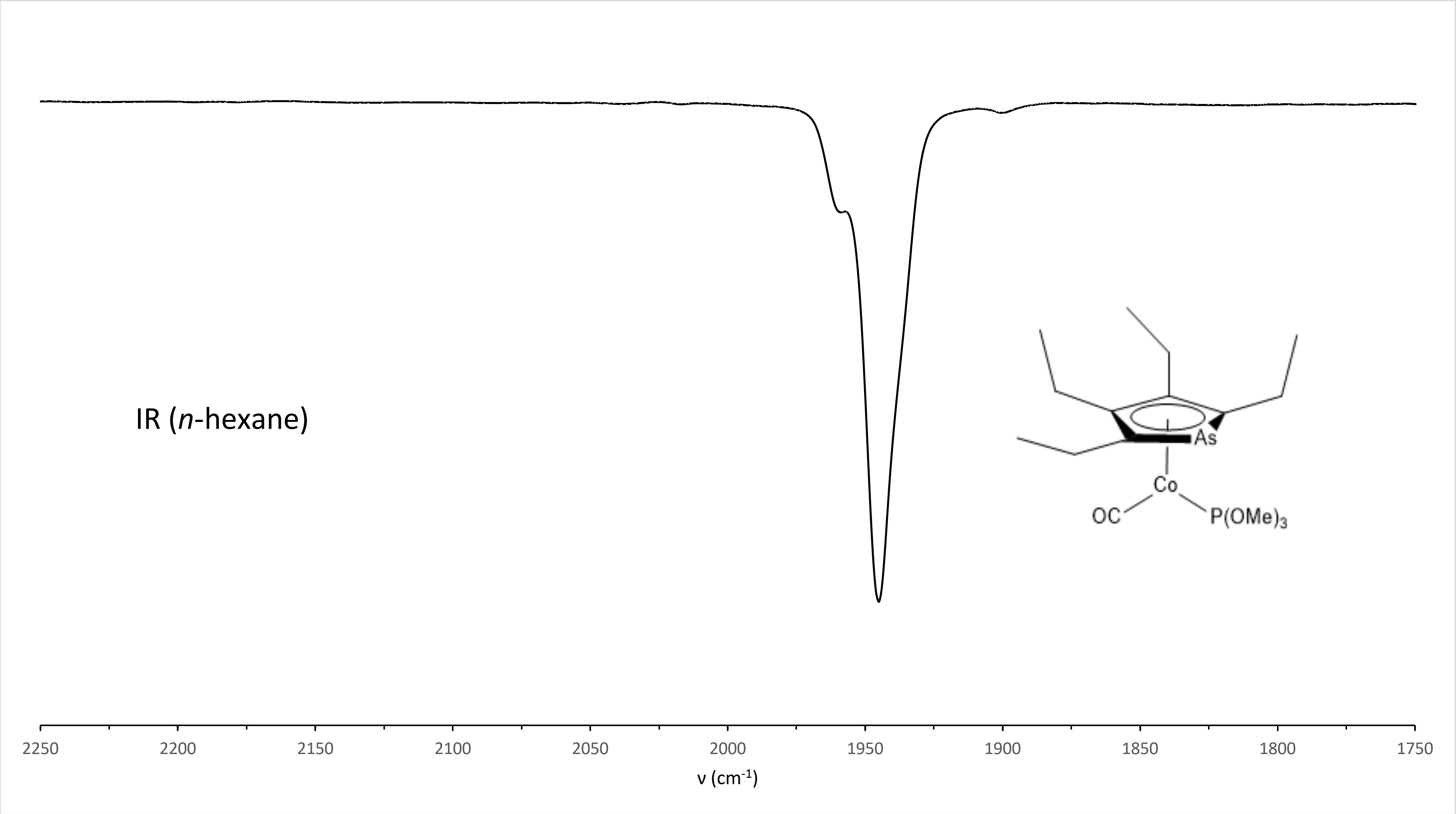
IR (n-hexane)
As
Co
OC
P(OMe)3
2250
2200
2150
2100
2050
2000
1950
1900
1850
1800
1750
ν (cm-1)

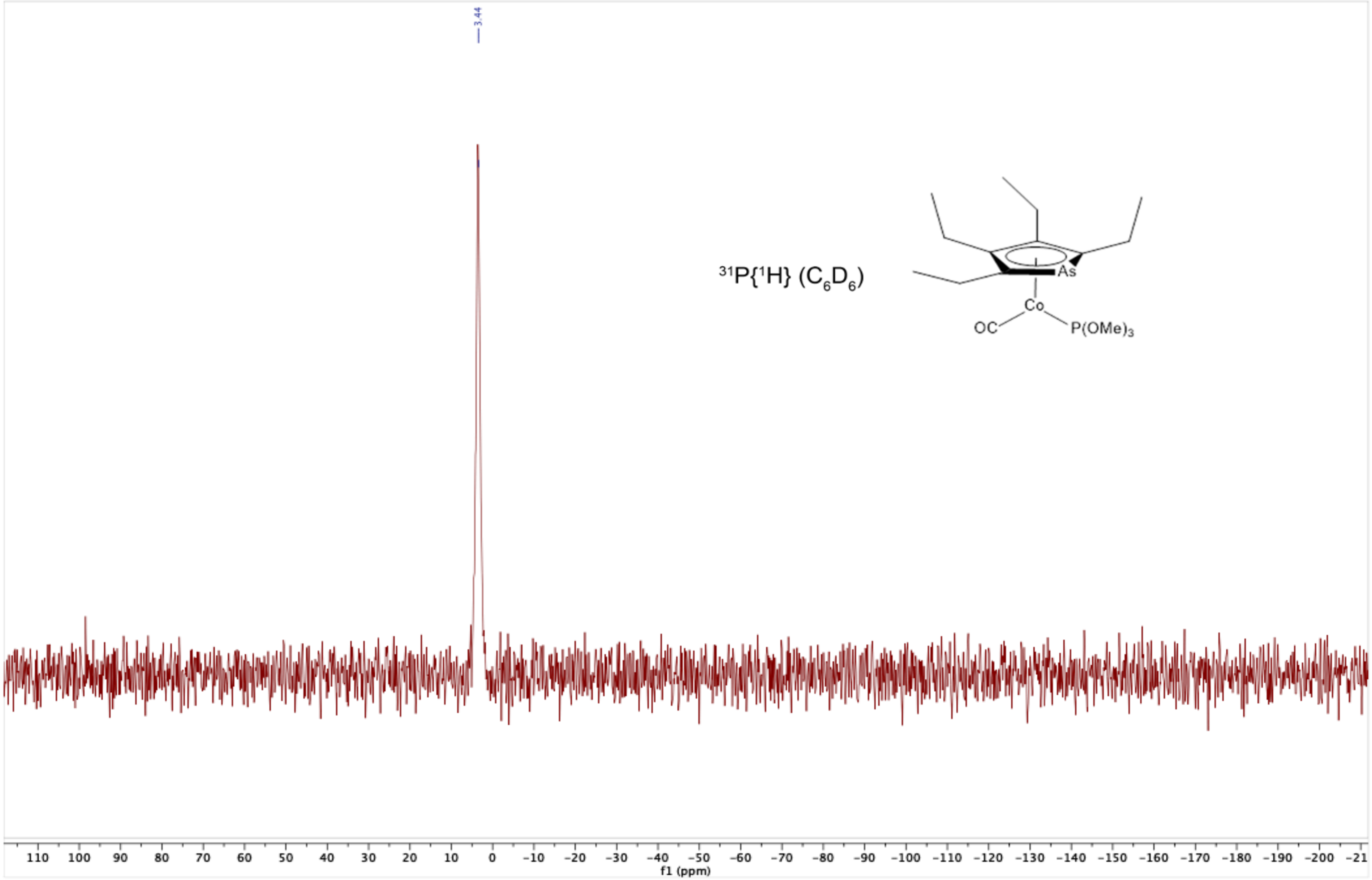
3.44
$^{31}P\{^{1}H\}$ (C_6D_6)
As
Co
OC
P(OMe)3
110 100 90 80 70 60 50 40 30 20 10 0 -10 -20 -30 -40 -50 -60 -70 -80 -90 -100 -110 -120 -130 -140 -150 -160 -170 -180 -190 -200 -21
f1 (ppm)

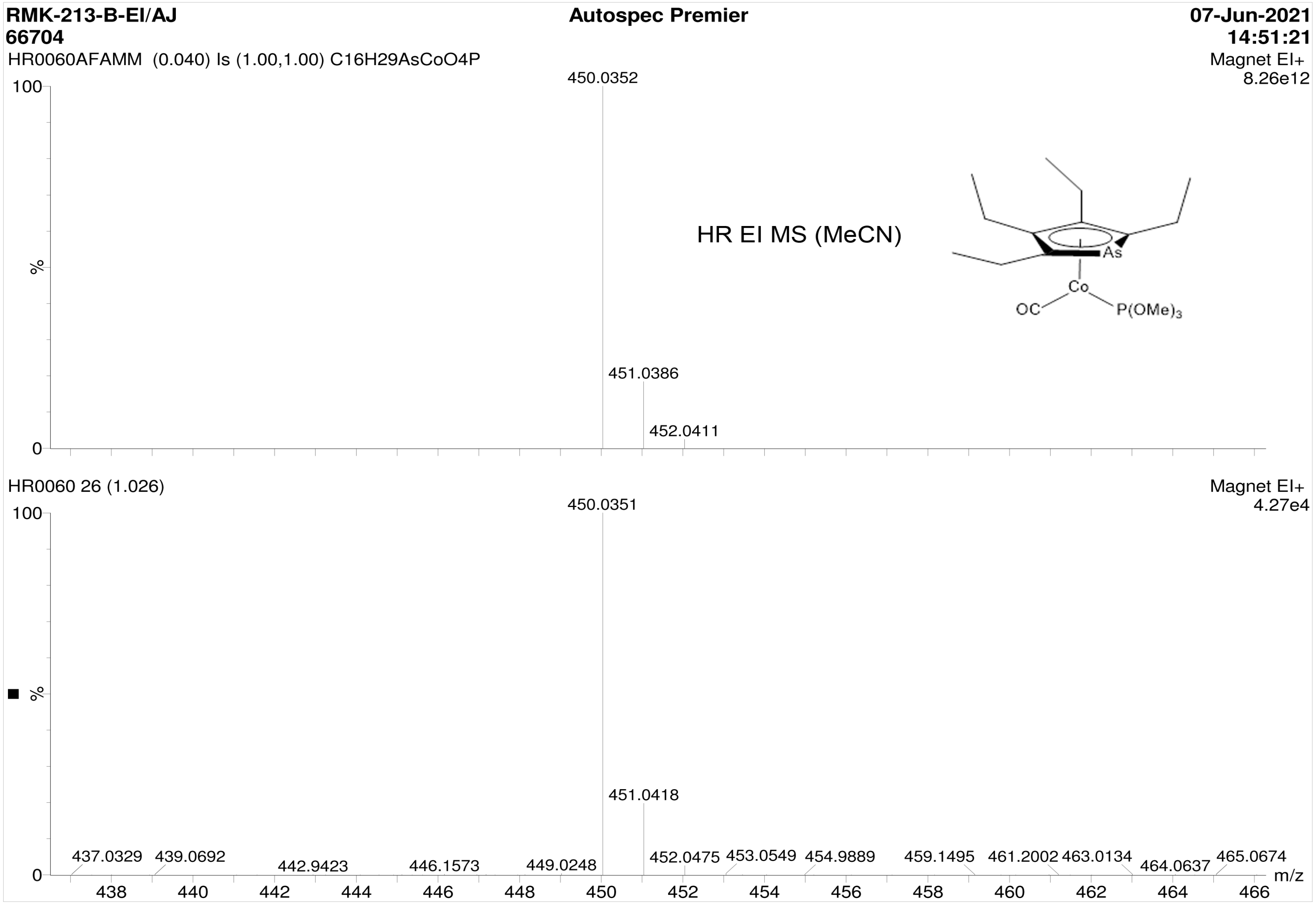
RMK-213-B-EI/AJ
66704
Autospec Premier
07-Jun-2021
14:51:21
HR0060AFAMM (0.040) Is (1.00,1.00) C16H29AsCoO4P
Magnet EI+
8.26e12
450.0352
451.0386
452.0411
HR EI MS (MeCN)
As
Co
OC
P(OMe)3
100
%
0
HR0060 26 (1.026)
Magnet EI+
4.27e4
450.0351
451.0418
437.0329
439.0692
442.9423
446.1573
449.0248
452.0475
453.0549
454.9889
459.1495
461.2002
463.0134
464.0637
465.0674
m/z
438
440
442
444
446
448
450
452
454
456
458
460
462
464
466

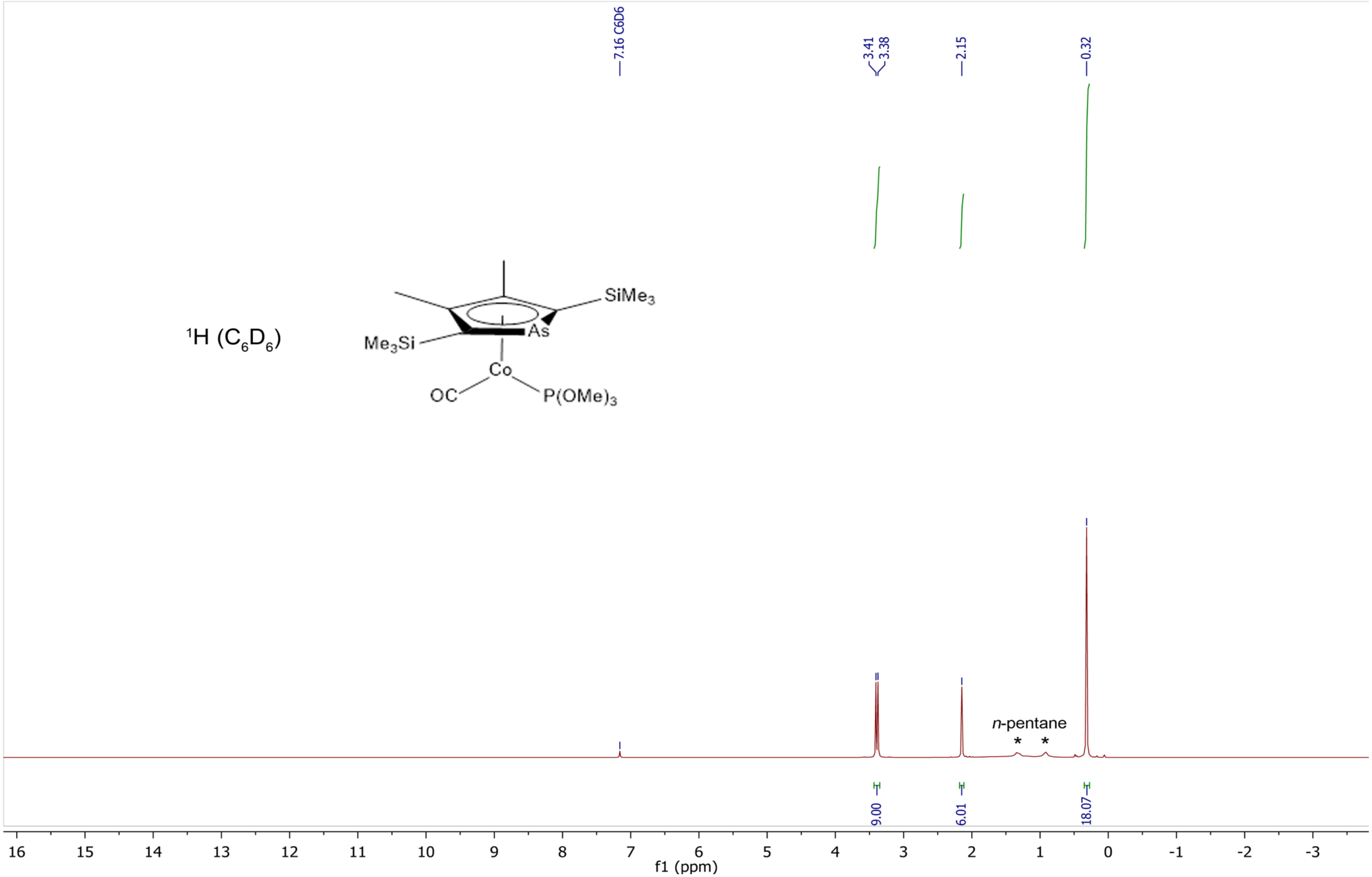
7.16 C6D6
3.41
3.38
2.15
0.32
¹H (C6D6)
SiMe3
Me3Si
As
Co
OC
P(OMe)3
n-pentane
9.00
6.01
18.07
16
15
14
13
12
11
10
9
8
7
6
5
4
3
2
1
0
-1
-2
-3
f1 (ppm)

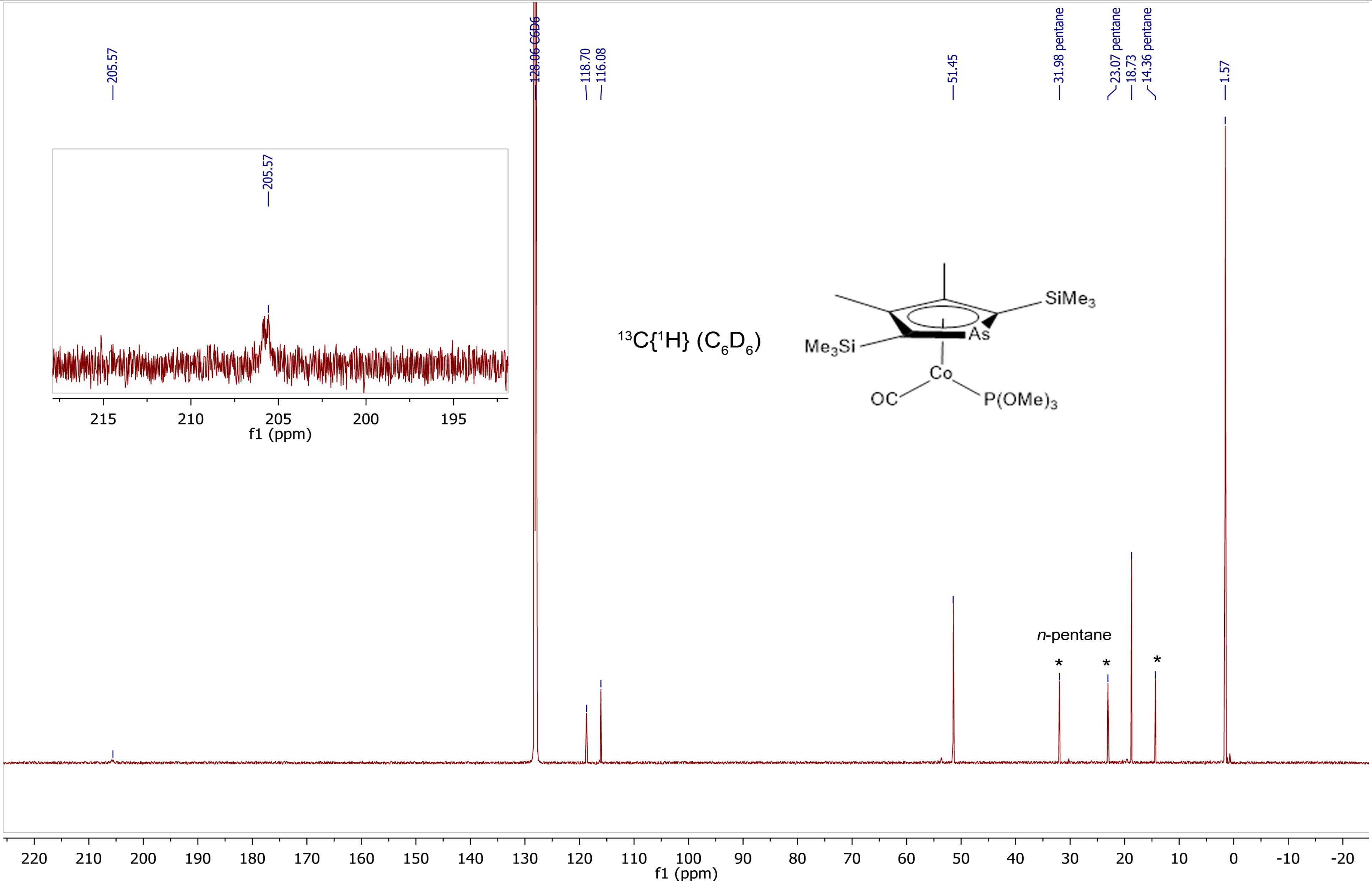
205.57
128.06 C6D6
118.70
116.08
51.45
31.98 pentane
23.07 pentane
18.73
14.36 pentane
1.57
205.57
215
210
205
200
195
f1 (ppm)
13C{1H} (C6D6)
SiMe3
As
Me3Si
Co
OC
P(OMe)3
n-pentane
*
*
*
220
210
200
190
180
170
160
150
140
130
120
110
100
90
80
70
60
50
40
30
20
10
0
-10
-20
f1 (ppm)

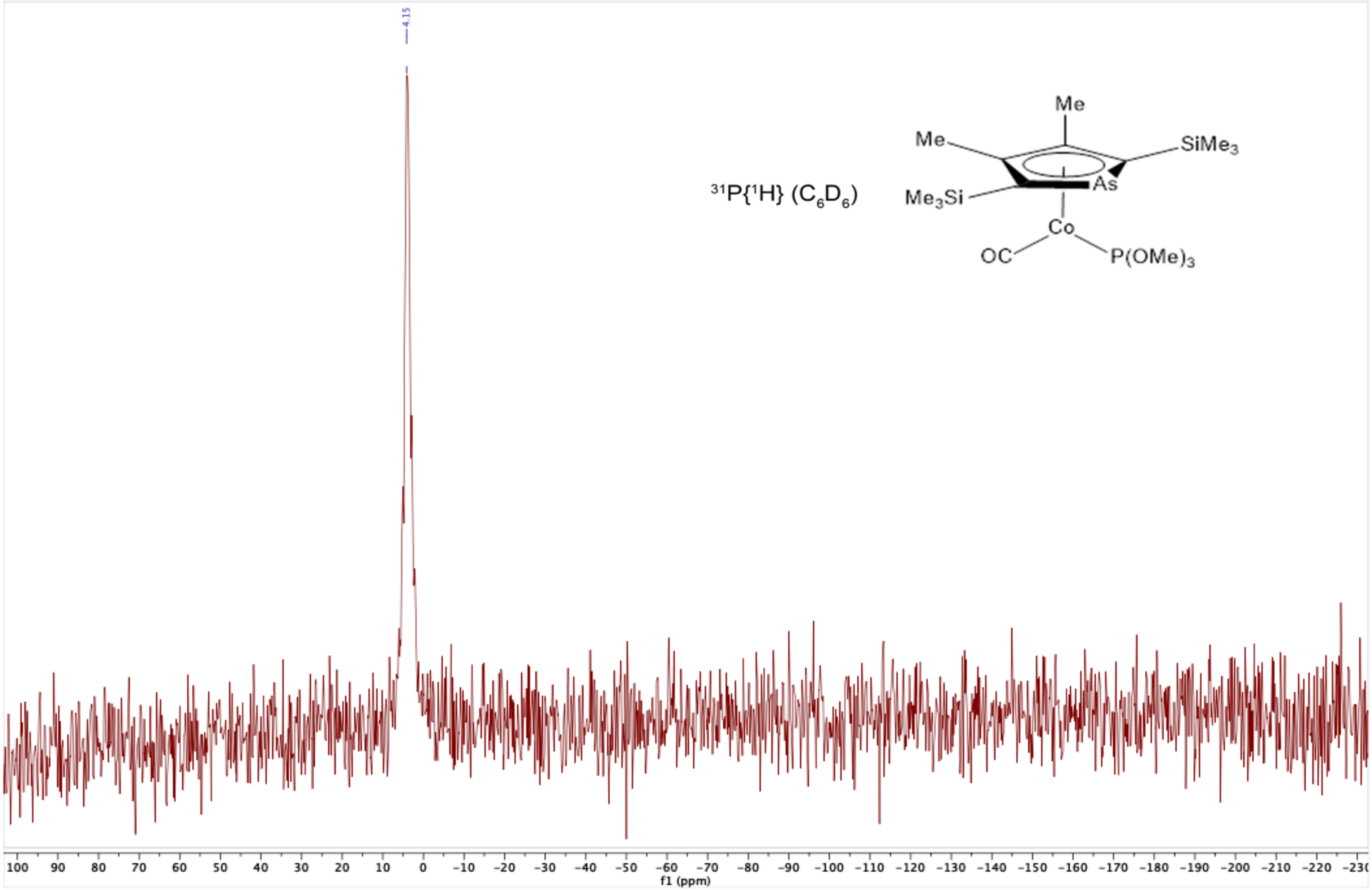
4.15
$^{31}P\{^1H\}$ (C_6D_6)
Me
Me
SiMe3
Me3Si
As
Co
OC
P(OMe)3
100 90 80 70 60 50 40 30 20 10 0 -10 -20 -30 -40 -50 -60 -70 -80 -90 -100 -110 -120 -130 -140 -150 -160 -170 -180 -190 -200 -210 -220
f1 (ppm)

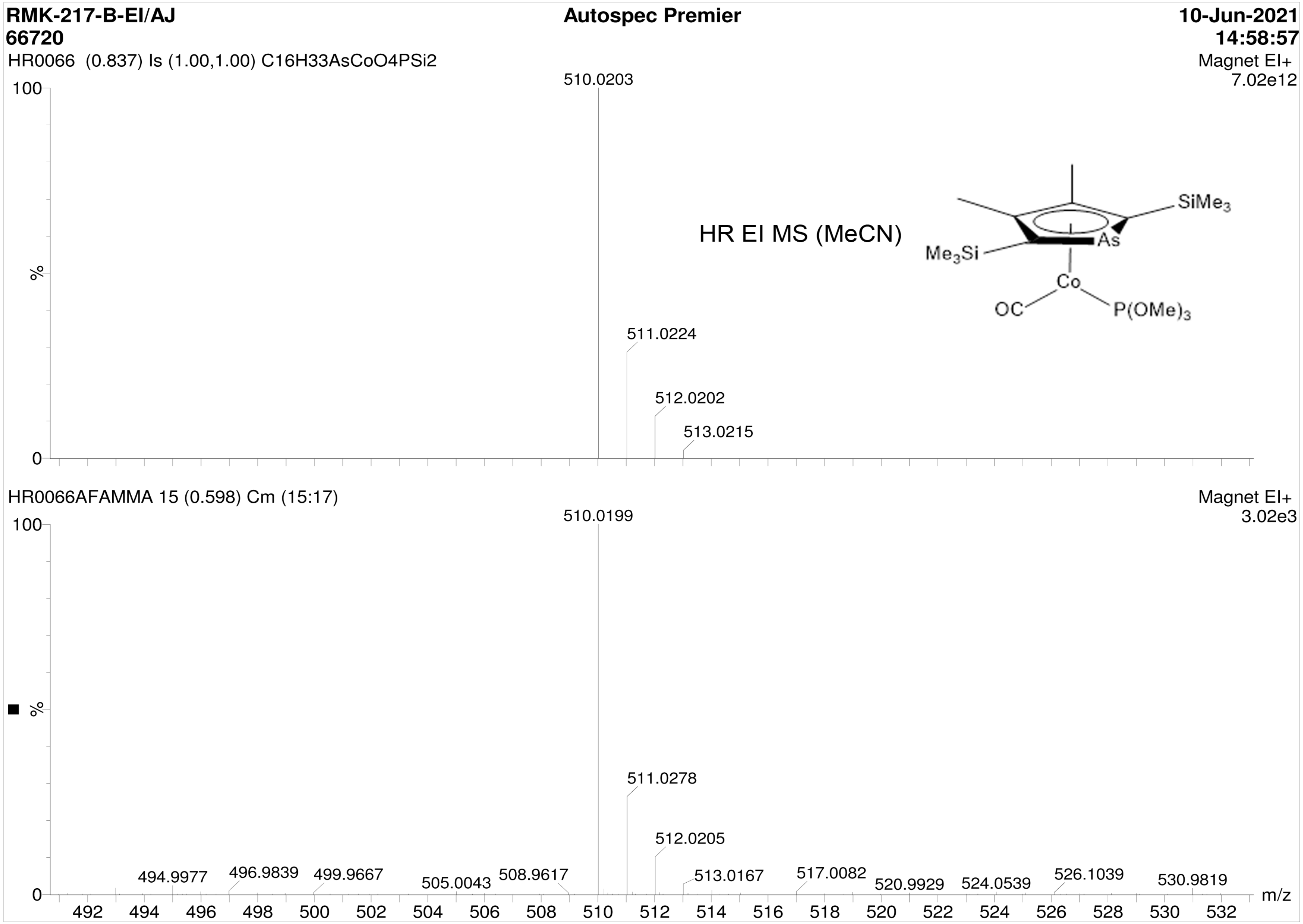
RMK-217-B-EI/AJ
66720
Autospec Premier
10-Jun-2021
14:58:57
HR0066 (0.837) Is (1.00,1.00) C16H33AsCoO4PSi2
Magnet EI+
7.02e12
510.0203
511.0224
512.0202
513.0215
HR EI MS (MeCN)
SiMe3
Me3Si
As
Co
OC
P(OMe)3
HR0066AFAMMA 15 (0.598) Cm (15:17)
Magnet EI+
3.02e3
510.0199
511.0278
512.0205
494.9977
496.9839
499.9667
505.0043
508.9617
513.0167
517.0082
520.9929
524.0539
526.1039
530.9819
100
%
0
m/z

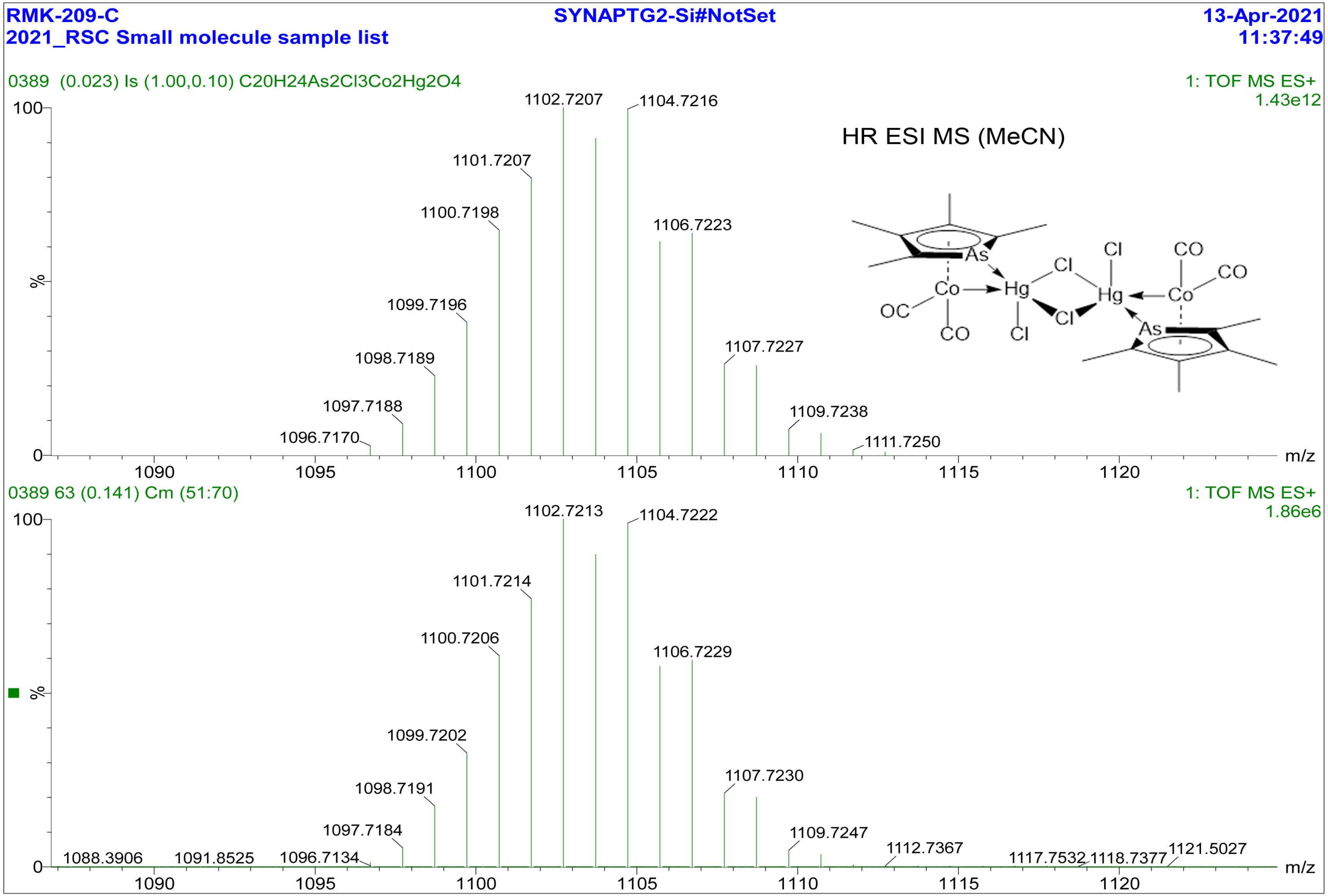
RMK-209-C
2021_RSC Small molecule sample list
SYNAPTG2-Si#NotSet
13-Apr-2021
11:37:49
0389 (0.023) Is (1.00,0.10) C20H24As2Cl3Co2Hg2O4
1: TOF MS ES+
1.43e12
HR ESI MS (MeCN)
1102.7207
1104.7216
1101.7207
1100.7198
1106.7223
1099.7196
1098.7189
1107.7227
1097.7188
1109.7238
1096.7170
1111.7250
m/z
0389 63 (0.141) Cm (51:70)
1: TOF MS ES+
1.86e6
1102.7213
1104.7222
1101.7214
1100.7206
1106.7229
1099.7202
1098.7191
1107.7230
1097.7184
1109.7247
1088.3906
1091.8525
1096.7134
1112.7367
1117.7532
1118.7377
1121.5027
%
m/z

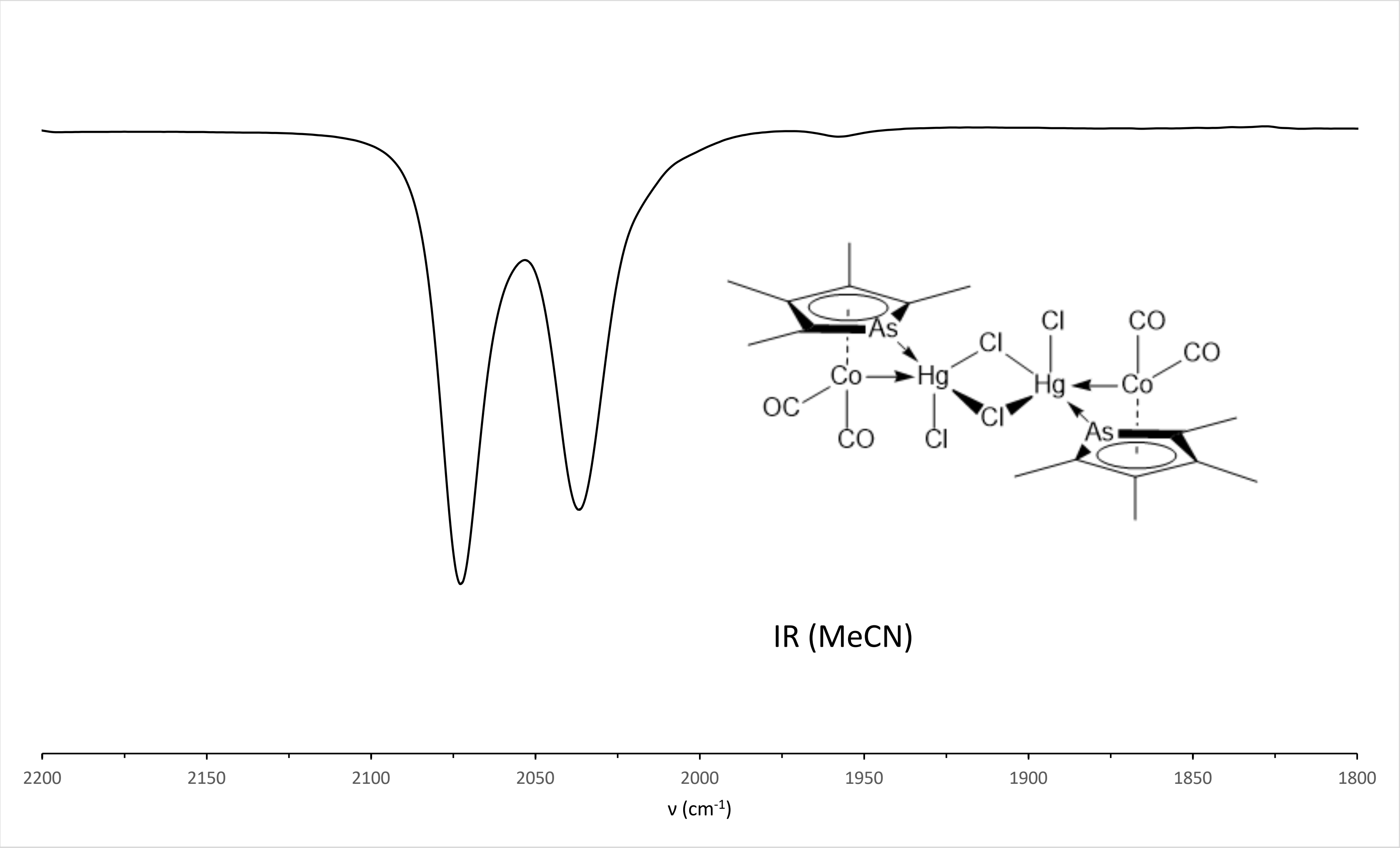

Cl
CO
As
Cl
CO
Co
Hg
Hg
Co
OC
Cl
As
CO
Cl
IR (MeCN)
2200
2150
2100
2050
2000
1950
1900
1850
1800
ν (cm-1)

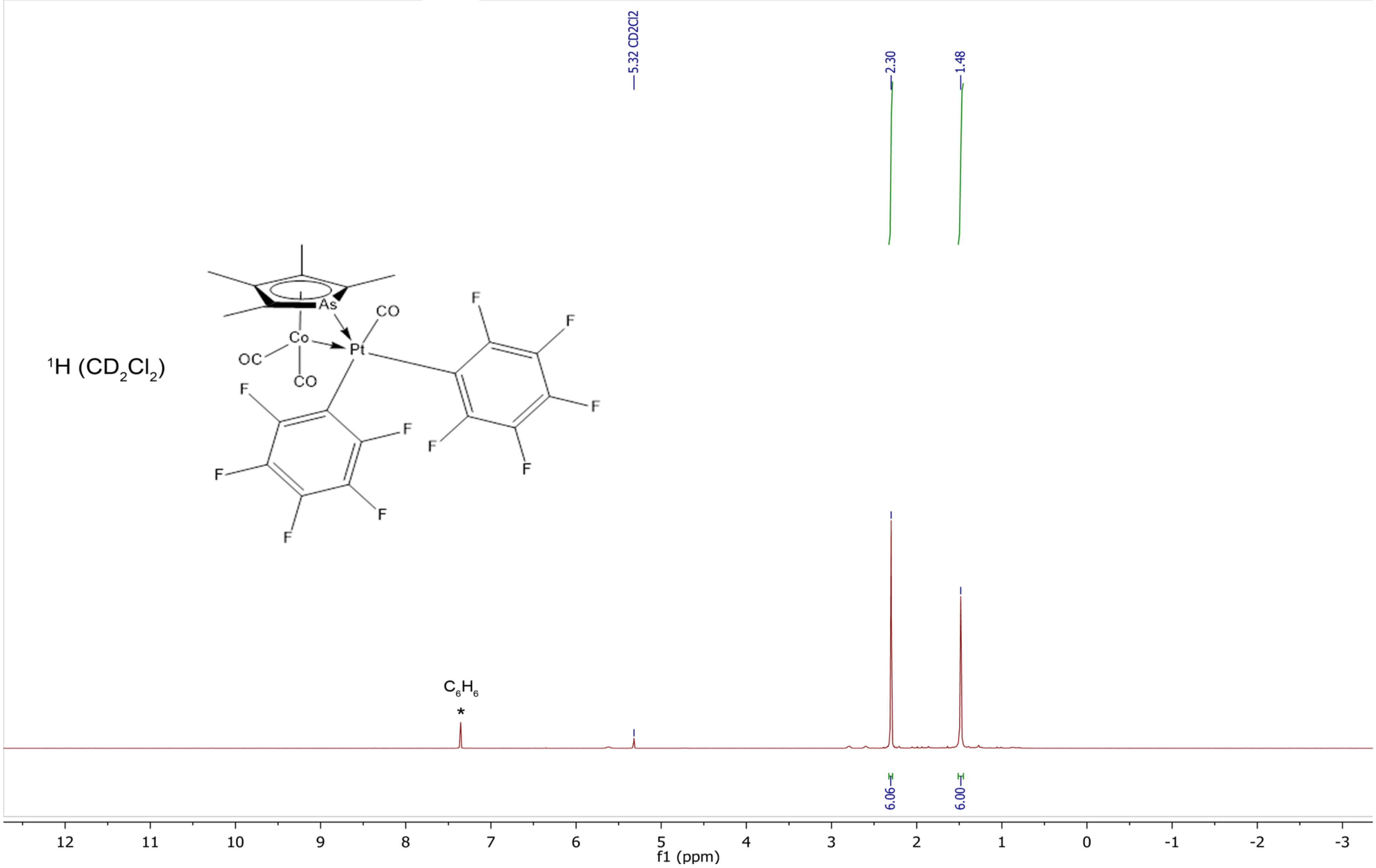

5.32 CD2Cl2
2.30
1.48
^{1}H (CD_2Cl_2)
As
Co
Pt
CO
OC
CO
F
C_6H_6
*
6.06
6.00
12
11
10
9
8
7
6
5
4
3
2
1
0
-1
-2
-3
f1 (ppm)

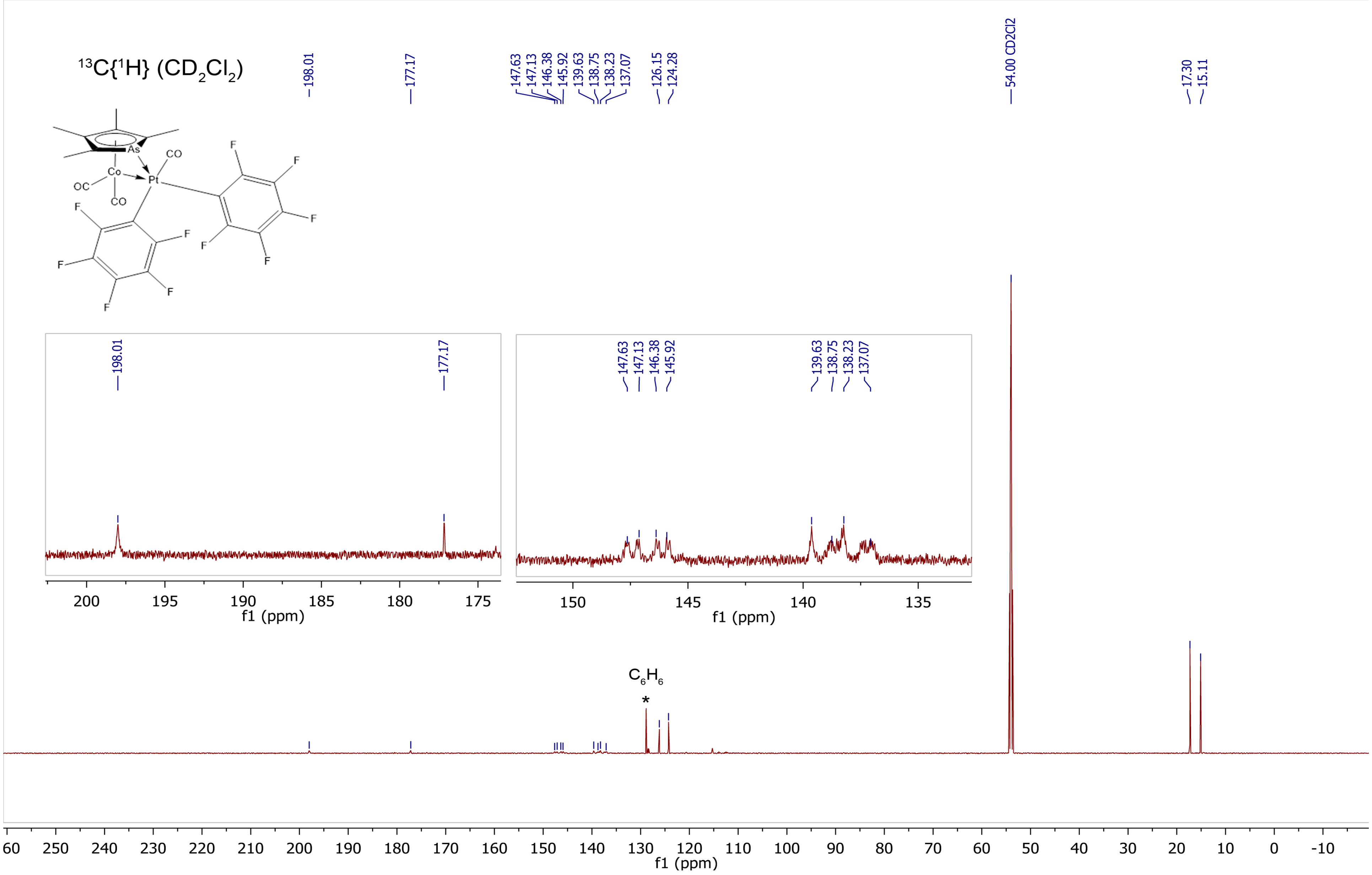

^{13}C{^{1}H} (CD$_2$Cl$_2$)
198.01
177.17
147.63
147.13
146.38
145.92
139.63
138.75
138.23
137.07
126.15
124.28
54.00 CD2Cl2
17.30
15.11
As
Co
Pt
CO
OC
F
C$_6$H$_6$
*
f1 (ppm)

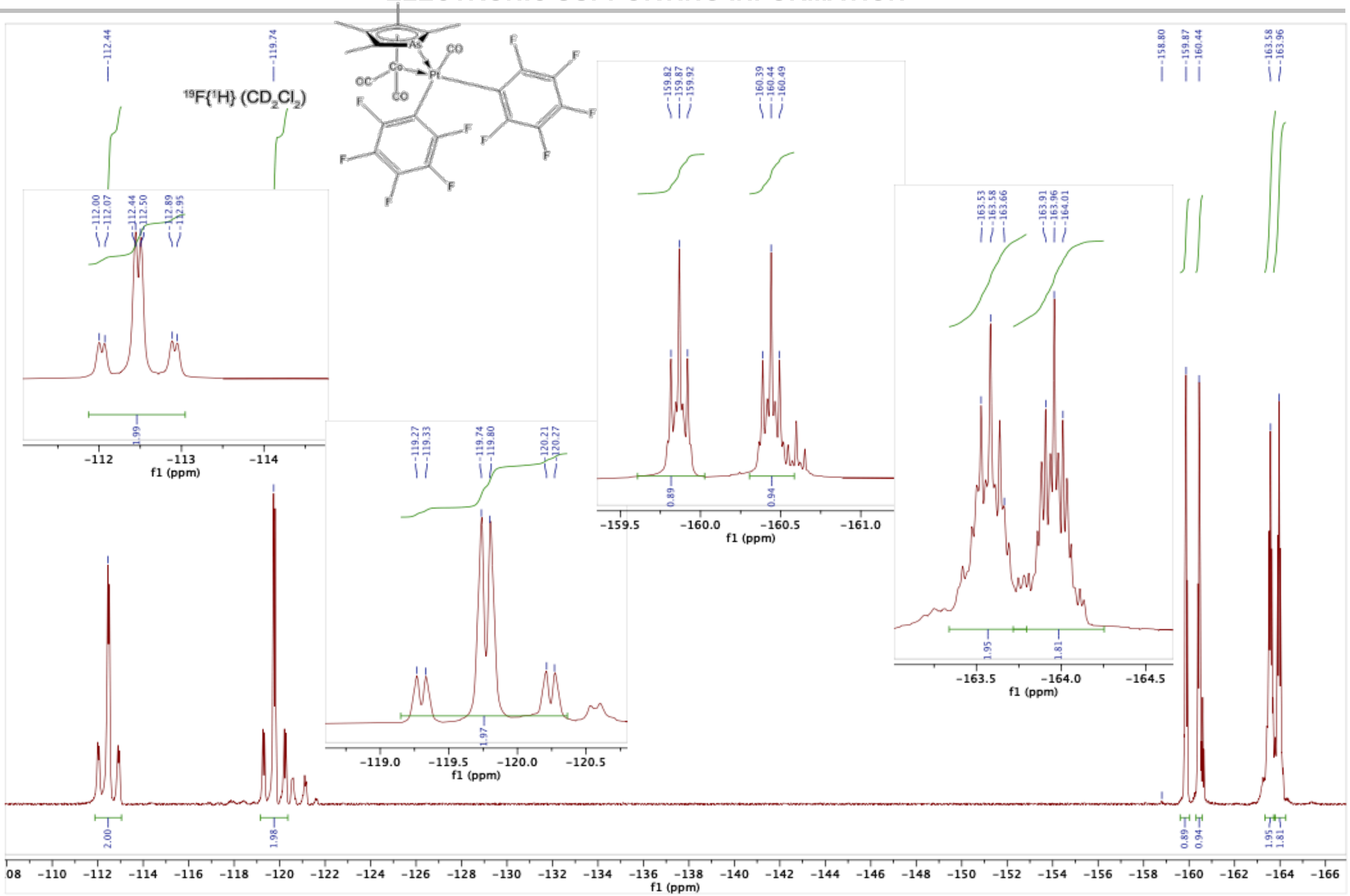

$^{19}F\{^1H\}$ (CD_2Cl_2)
f1 (ppm)

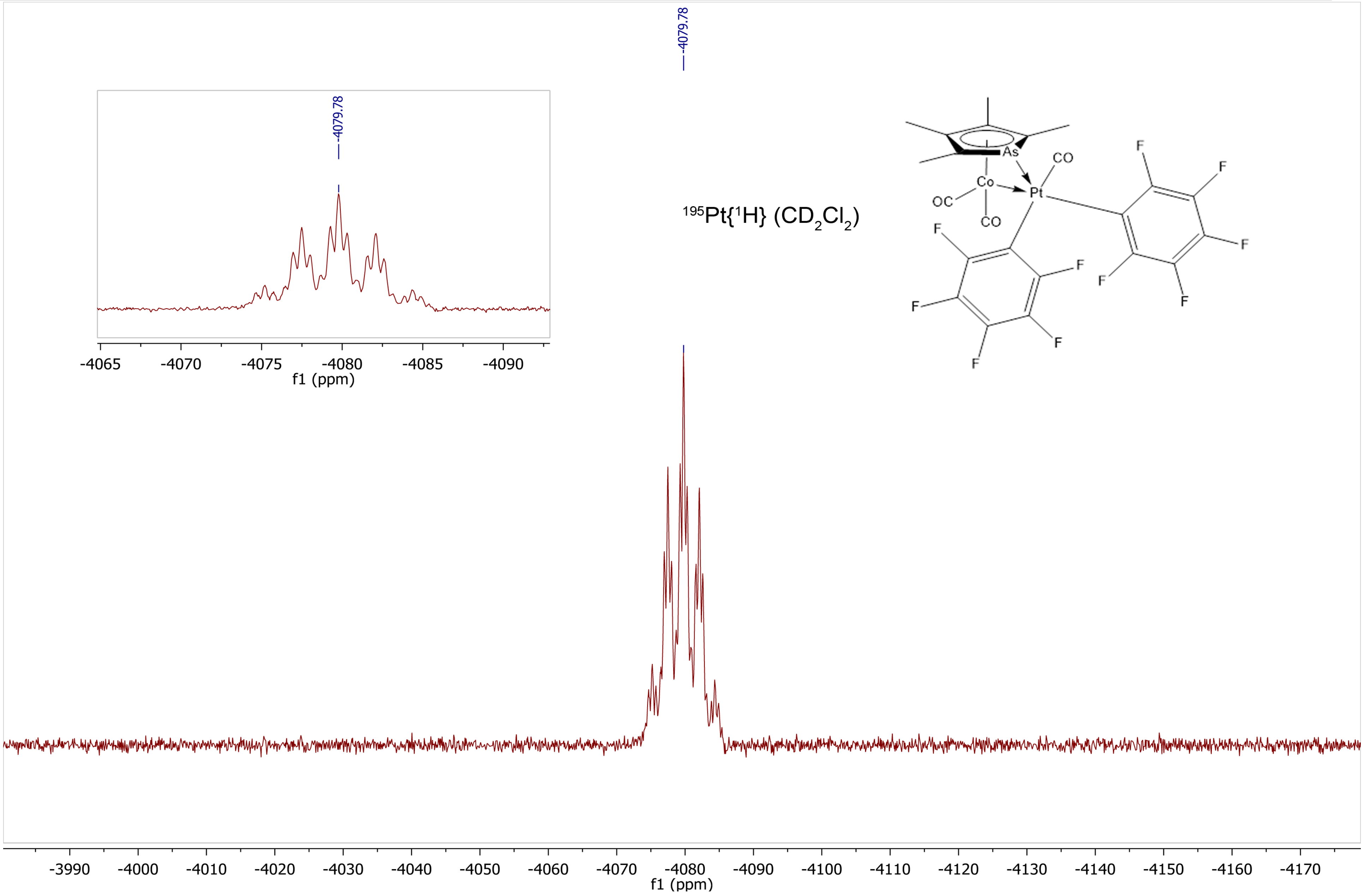

-4079.78
195Pt{1H} (CD2Cl2)
-4065 -4070 -4075 -4080 -4085 -4090
f1 (ppm)
As
Co
Pt
CO
OC
F
-3990 -4000 -4010 -4020 -4030 -4040 -4050 -4060 -4070 -4080 -4090 -4100 -4110 -4120 -4130 -4140 -4150 -4160 -4170
f1 (ppm)

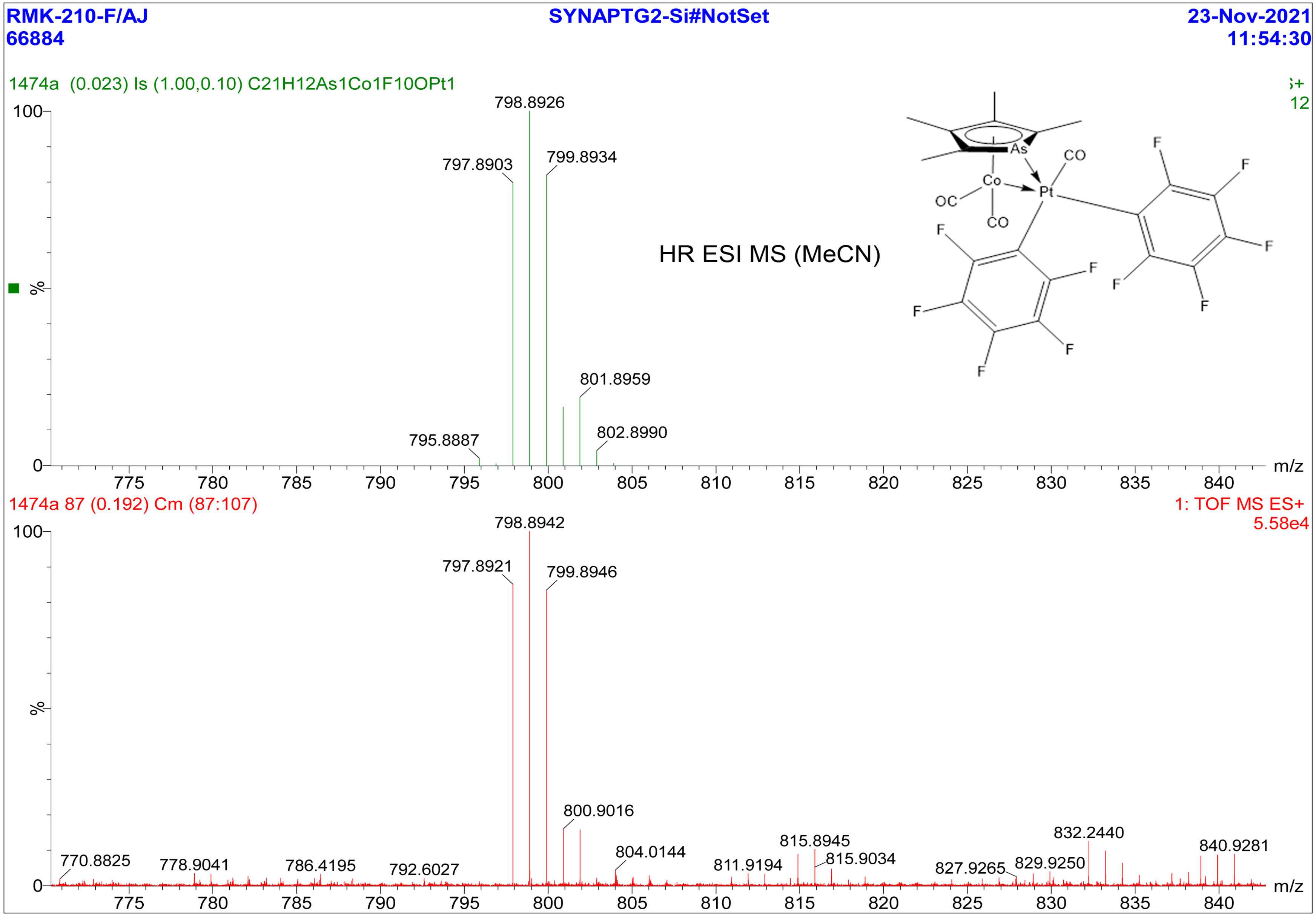

RMK-210-F/AJ
66884
SYNAPTG2-Si#NotSet
23-Nov-2021
11:54:30
1474a (0.023) Is (1.00,0.10) C21H12As1Co1F10OPt1
798.8926
797.8903
799.8934
801.8959
795.8887
802.8990
HR ESI MS (MeCN)
As
Co
Pt
CO
OC
F
100
%
0
m/z
775
780
785
790
795
800
805
810
815
820
825
830
835
840
1474a 87 (0.192) Cm (87:107)
1: TOF MS ES+
5.58e4
798.8942
797.8921
799.8946
800.9016
804.0144
815.8945
815.9034
832.2440
840.9281
770.8825
778.9041
786.4195
792.6027
811.9194
827.9265
829.9250

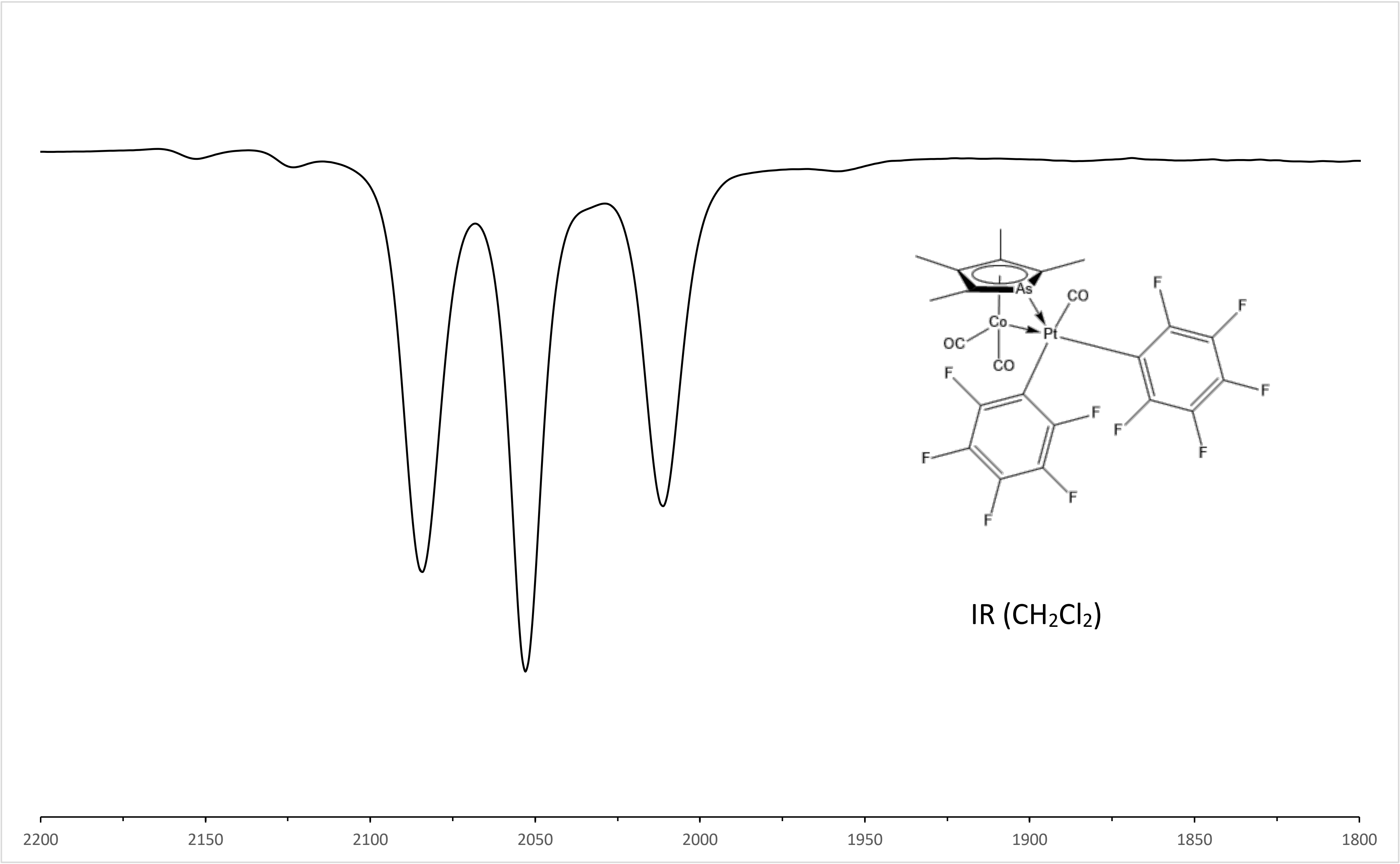
As
Co
Pt
CO
OC
CO
CO
F
F
F
F
F
F
F
F
F
F
IR (CH_2Cl_2)
2200
2150
2100
2050
2000
1950
1900
1850
1800

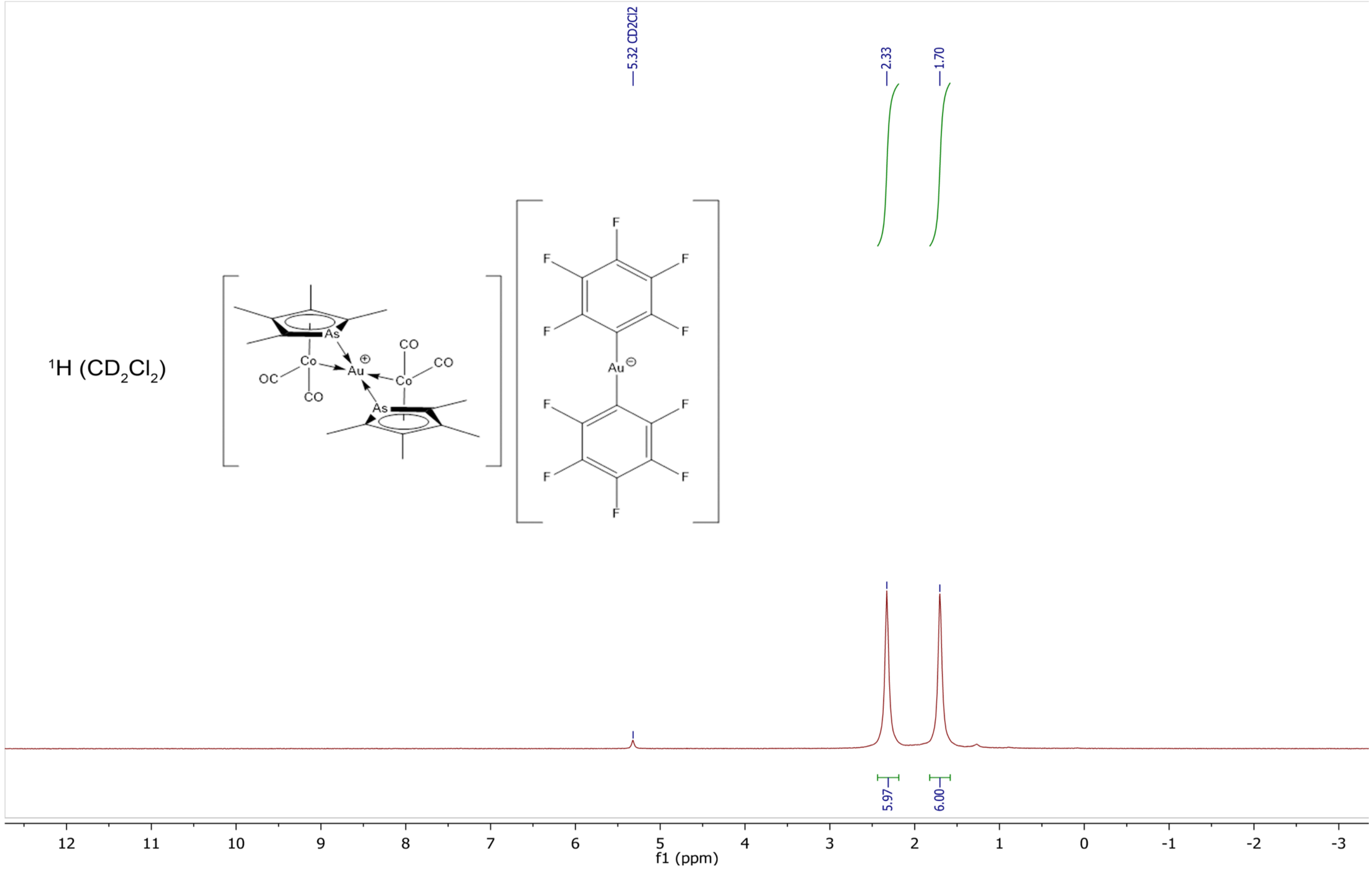

1H (CD2Cl2)
5.32 CD2Cl2
2.33
1.70
5.97
6.00
12 11 10 9 8 7 6 5 4 3 2 1 0 -1 -2 -3
f1 (ppm)
As
Co
Au
CO
OC
F

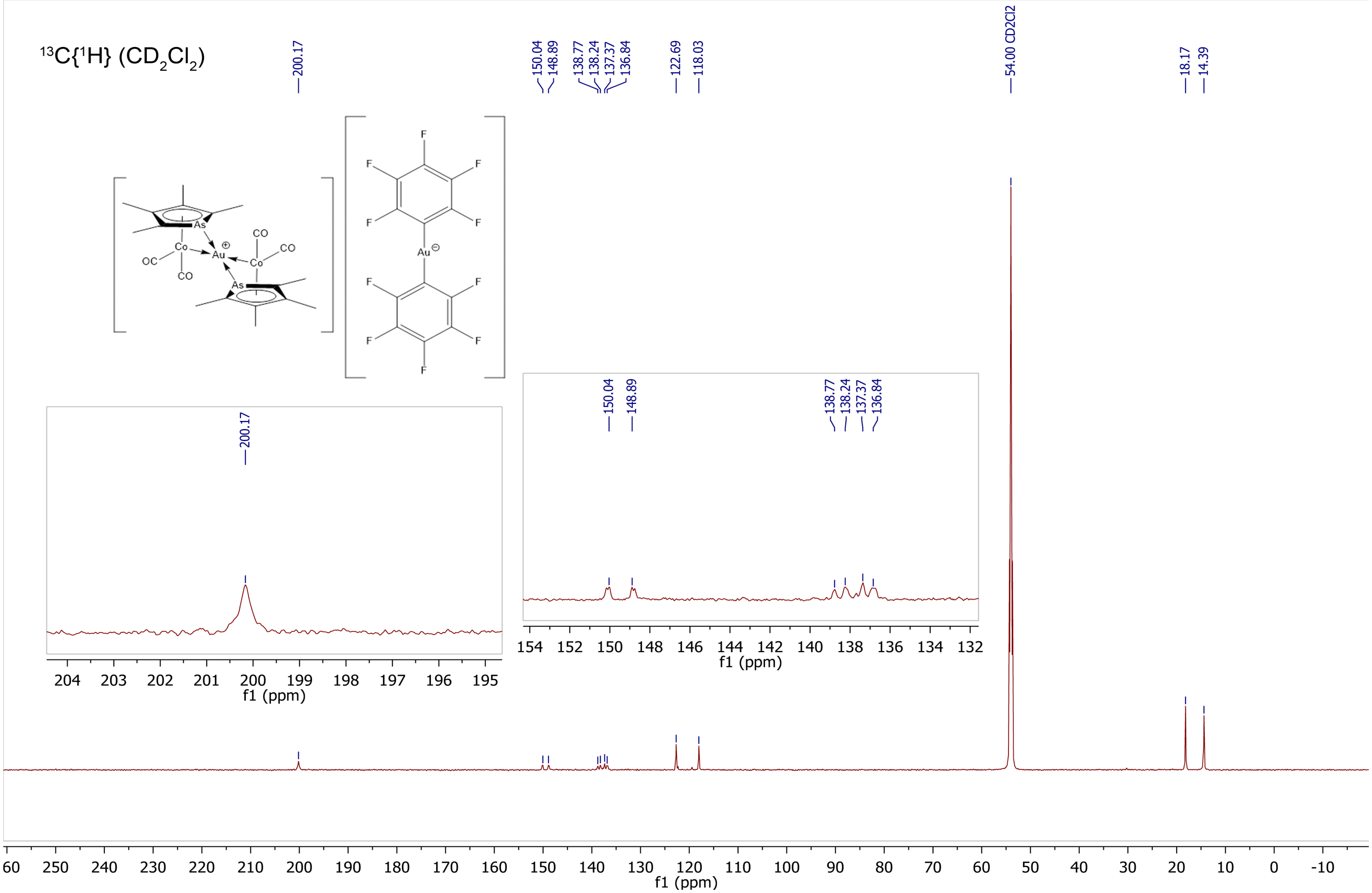
$^{13}C\{^{1}H\}$ (CD_2Cl_2)
200.17
150.04
148.89
138.77
138.24
137.37
136.84
122.69
118.03
54.00 CD2Cl2
18.17
14.39
f1 (ppm)

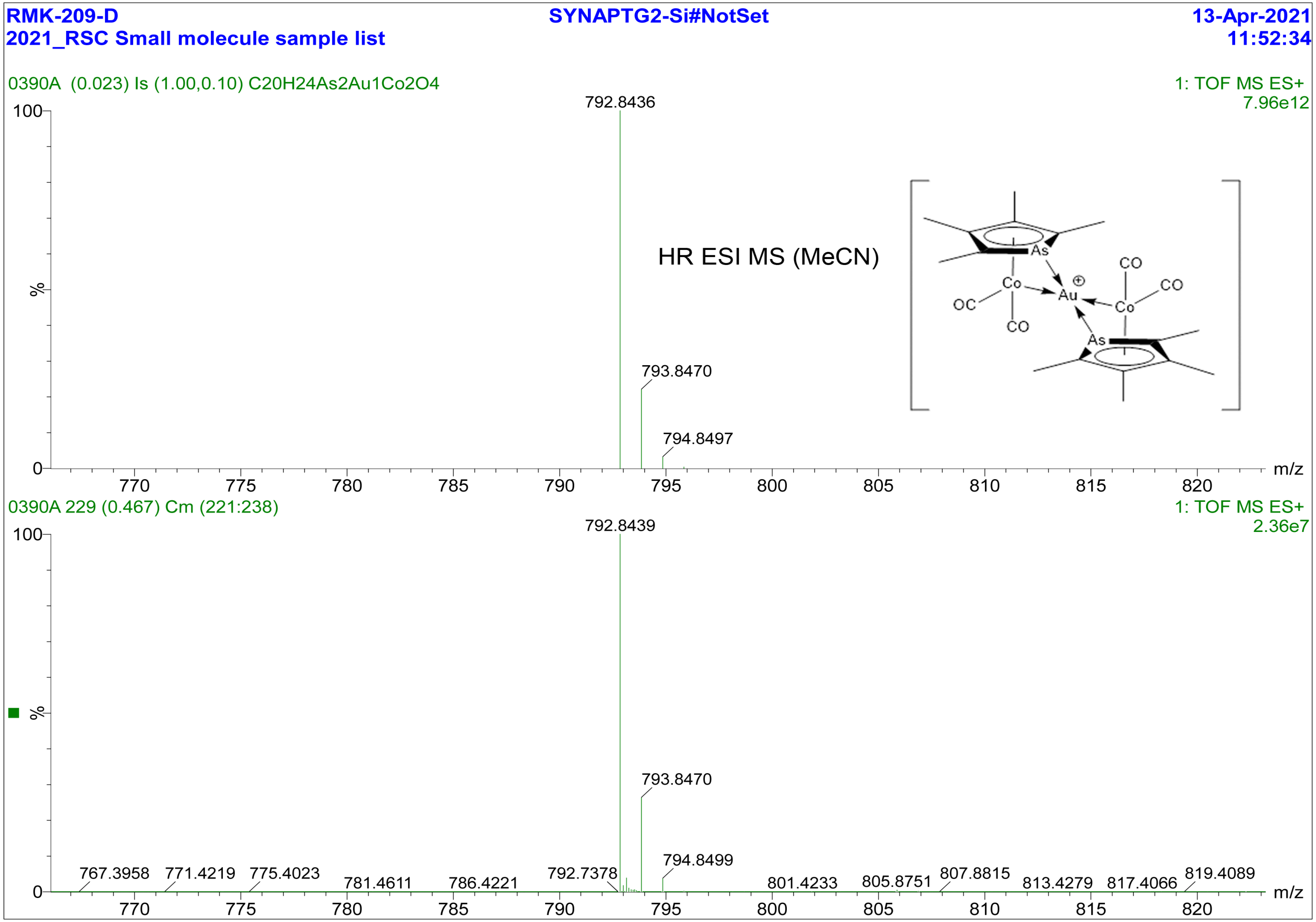

RMK-209-D
2021_RSC Small molecule sample list
SYNAPTG2-Si#NotSet
13-Apr-2021
11:52:34
0390A (0.023) Is (1.00,0.10) C20H24As2Au1Co2O4
1: TOF MS ES+
7.96e12
792.8436
793.8470
794.8497
HR ESI MS (MeCN)
As
Co
Au
CO
OC
m/z
%
0390A 229 (0.467) Cm (221:238)
1: TOF MS ES+
2.36e7
792.8439
793.8470
794.8499
767.3958
771.4219
775.4023
781.4611
786.4221
792.7378
801.4233
805.8751
807.8815
813.4279
817.4066
819.4089

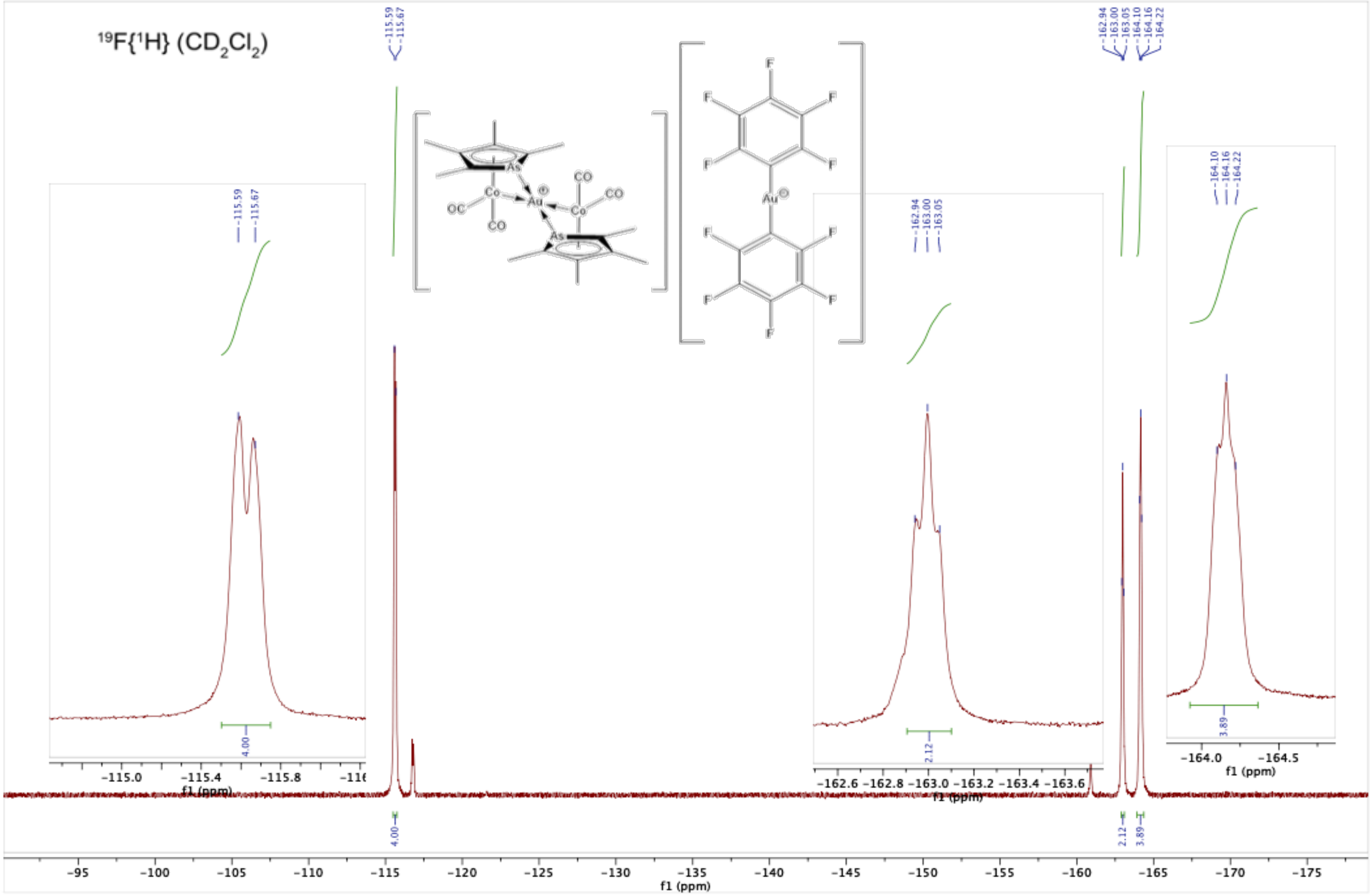

$^{19}F\{^1H\}$ (CD_2Cl_2)
-115.59
-115.67
-162.94
-163.00
-163.05
-164.10
-164.16
-164.22
4.00
2.12
3.89
f1 (ppm)

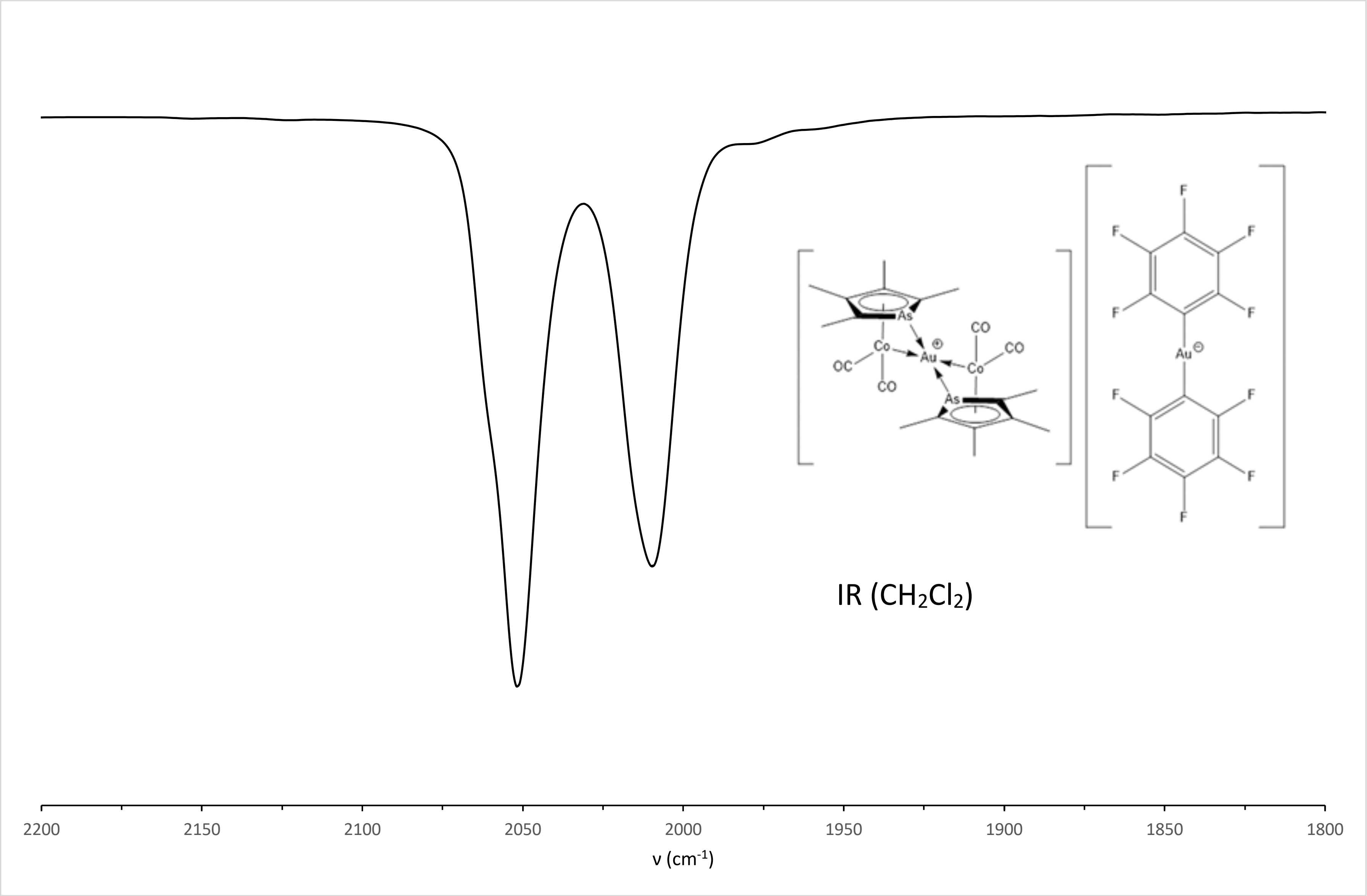
IR (CH_2Cl_2)
2200
2150
2100
2050
2000
1950
1900
1850
1800
ν (cm^{-1})
As
Co
Au
CO
OC
F
Au

6.

7. Author Contributions

RMK was responsible for the conceptualisation and execution of the experimental research, the acquisition and critical analysis of the characterisational data and compilation of the original draft. AFH was responsible for funding acquisition, project administration, validation and refinements to the manuscript.

8. References

1 a) H. Imoto, A. Urushizaki, I. Kawashima, K. Naka, *Chem. –Eur. J.,* **2018**, *24*, 8797-8803; b) P. J. Fagan, W. A. Nugent, J. C. Calabrese, *J. Am. Chem. Soc.,* **1994**, *116*, 1880-1889; c) M. Westerhausen, C. Gückel, H. Piotrowski, P. Mayer, M. Warchhold, H. Nöth, *Z. Anorg. Allg. Chem.,* **2001**, *627*, 1741-1750; d) M. Westerhausen, M. H. Digeser, C. Gückel, H. Nöth, J. Knizek, W. Ponikwar, *Organometallics* **1999**, *18*, 2491-2496.

2 a) E. H. Braye, W. Hübel, I. Caplier, *J. Am. Chem. Soc.,* **1961**, *83*, 4406-4413; b) F. C. Leavitt, T. A. Manuel, F. Johnson, L. U. Matternas, D. S. Lehman, *J. Am. Chem. Soc.,* **1960**, *82*, 5099-5102.

3 *Spartan 20*® (2020) Wavefunction, Inc., 18401 Von Karman Ave., Suite 370 Irvine, CA 92612 U.S.A

4 J.-D. Chai and M. Head-Gordon, *J. Chem. Phys.*, **2008**, *128*, 0841061-18410615; (*b*) J.-D. Chai and M. Head-Gordon, *Phys. Chem. Chem. Phys.*, **2008**, *10*, 6615- 6620.

5 a) P. J. Hay and W.R. Wadt, *J. Chem. Phys.*, **1985**, *82*, 270-283; b) W. R. Wadt and P. J. Hay, *J. Chem. Phys.* **1985**, *82*, 284-298; c) P. J. Hay, W. R. Wadt, *J. Chem. Phys.,* **1985**, *82*, 299-310.

6 W. J. Hehre, R. Ditchfeld and J. A. Pople, *J. Chem. Phys.*, 1972, **56**, 2257-2261

7 a) I. R. Lyatifov, G. M. Jafarov, V. N. Babin, P. V. Petrovskii, V. D. Zagorevskii, *J. Organomet. Chem.,* **1989**, *368*, 223-230; b) R. B. King, M. B. Bisnette, *J. Organomet. Chem.,* **1967**, *8*, 287-297.

8 J. W. Chambers, A. J. Baskar, S. G. Bott, J. L. Atwood, M. D. Rausch, *Organometallics* **1986**, *5*, 1635-1641.

9 C. Charrier, H. Bonnard, F. Mathey, D. Neibecker, *J. Organomet. Chem.,* **1982**, *231*, 361-367.

10 A. J. M. Caffyn, D. Carmichael, F. Mathey, L. Ricard, *Organometallics* **1997**, *16*, 2049-2054.

11 *CrysAlisPRO®,* Oxford Diffraction/Agilent Technologies UK Ltd., Yarton, England, **2010**.

12 O. V. Dolomanov, L. J. Bourhis, R. J. Gildea, J. A. K. Howard, H. Puschmann, *J. Appl. Crystallog.,* **2009**, *42*, 339-341.

13 G. Sheldrick, *Acta Crystallog. Sect. A* **2015**, *71*, 3-8.

14 G. Sheldrick, *Acta Crystallog. Sect. C* **2015**, *71*, 3-8.
